# Mapping Steroidogenic Perturbations Under Endocrine Disruptor Mixtures Across Demographic Subgroups: Structural and Metabolomic Insights

**DOI:** 10.1002/advs.76381

**Published:** 2026-07-09

**Authors:** Yanling Chen, Lei Huang, Yingtong Jiang, Guohong Zhao, Mengyuan Zhu, Xiulian Lu, Lang Lang, Akinpelumi Oluwatobiloba Esther, Longhao Fan, Yuqi Jiang, Chang Sun, Xiaoling Zhang, Kun Zhou, Xiaoming Ji, Minjian Chen

**Affiliations:** ^1^ State Key Laboratory of Reproductive Medicine and Offspring Health Center For Global Health School of Public Health Nanjing Medical University Nanjing China; ^2^ Key Laboratory of Modern Toxicology of Ministry of Education Nanjing Medical University Nanjing China; ^3^ Department of Hygienic Analysis and Detection School of Public Health Nanjing Medical University Nanjing China; ^4^ Department of Epidemiology Center For Global Health School of Public Health Nanjing Medical University Nanjing China; ^5^ Department of Occupational Medicine and Environmental Health School of Public Health Key Laboratory of Public Health Safety and Emergency Prevention and Control Technology of Higher Education Institutions in Jiangsu Province Nanjing Medical University Nanjing China

**Keywords:** EDCs, gonadal steroidogenesis, mechanism, metabolism, metabolomics, mixed exposure, structural modeling

## Abstract

Endocrine‐disrupting chemicals (EDCs) are pervasive environmental chemicals affecting hormone homeostasis, yet mapping mixture exposures to mechanisms at the population scale remains challenging. Here, analyses of 4255 U.S. participants are integrated with computational and experimental validation, linking population signals to molecular targets. Profiling 37 EDCs reveals consistent associations between phthalate metabolites and lower testosterone, strongest in adult males. Mono‐(3‐carboxypropyl) phthalate (MCPP) is identified as a key disruptor and is shown to inhibit CYP17A1, consistent with engagement of the enzyme's iron–protoporphyrin IX (heme) center. Docking/molecular dynamics prioritizes this interaction, which is supported by surface plasmon resonance, multi‐matrix metabolomics in vivo and in vitro, and functional assays in primary Leydig cells. An interactive web resource (https://chembio.njmu.edu.cn/edc‐hormone‐explorer.html) provides open access to the association landscape and supports risk prioritization.

## Graphical abstract

Population‐scale EDC–hormone association mapping among 4255 participants across demographic subgroups identifies phthalate metabolites as the most consistent signals associated with lower testosterone‐related markers in males. Integrative mechanistic validation prioritizes MCPP as a key phthalate metabolite linked to CYP17A1‐centered disruption of Leydig‐cell steroidogenic regulation and reduced testosterone synthesis.

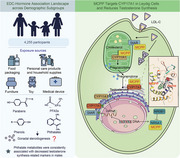

## 1 Introduction

Gonadal hormones are essential for reproductive health, development, and metabolic functions across the life course, affecting both physical states [1]. In children, they are crucial for proper growth and future reproductive abilities [2]. Dysregulation in these hormones can lead to sexual dysfunction [3] and developmental impairments [4]. Infertility, a growing global concern, is often linked to gonadal hormone abnormalities [5]. Testosterone (T) shapes libido and sexual behavior, with low levels observed in 20%–30% of male infertility cases [6]. Estradiol (E2) supports reproductive health [7]. Both T and E2 are crucial for sexual function and reproductive health in both sexes, positioning gonadal endocrinology alongside the major determinants of population well‐being that are routinely tracked in nationally representative cohorts.

Endocrine‐disrupting chemicals (EDCs), such as phenols, parabens, phthalate esters (PAEs), and per‐ and polyfluoroalkyl substances (PFASs), are commonly encountered [8]. EDCs exposure poses significant risks to reproductive and metabolic health at any life stage, with variability shaped by absorption, distribution, metabolism, and excretion (ADME) as well as age‐related biology [9]. Responses to EDCs can also differ by sex; males may suffer from reduced sperm quality or sexual dysfunction, while females might face irregular menstruation or breast disorders [10, 11]. Beyond individual chemicals, global assessments have flagged synthetic chemical mixtures as an emergent environmental challenge with broad health relevance—placing EDCs among high‐priority risks that warrant population‐scale evaluation, analogous to how lead [12], obesity, and mortality [13] have been interrogated using U.S. nationally representative data. Yet most studies examine single EDCs or narrow classes in specific populations. Given that gonadal hormone levels arise from complex steroid biosynthesis, the landscape‐level impact of real‐world mixtures on human steroidogenesis remains insufficiently resolved across diverse groups.

Against this backdrop, our study is designed to provide population‐to‐mechanism insight into mixed EDC effects on gonadal steroidogenesis, while preserving direct relevance to U.S. public health. We examined associations between mixed exposure to 37 common EDCs (phenols, parabens, PAEs, and PFASs) and molecules in the gonadal steroidogenic metabolic pathway across sexes and ages. The selection of EDCs was based on their widespread use in daily life, potential impact, and recently reported endocrine‐disrupting mixtures [14]. Gonadal steroidogenesis utilizes cholesterol from various sources, including low‐density lipoprotein cholesterol (LDL‐C) and high‐density lipoprotein cholesterol (HDL‐C). In the context of the Friedewald equation, very low‐density lipoprotein cholesterol (VLDL‐C) is estimated as triglycerides (TG) divided by 5 (in mg/dL) [15]. Therefore, total Cholesterol (TC) represents the sum of LDL‐C, HDL‐C, and VLDL‐C (calculated as TG/5).

Therefore, the molecules involved in this study included T, E2, sex hormone binding globulin (SHBG), LDL‐C, HDL‐C, TG, and TC, covering the process from cholesterol use to gonadal hormone production and transportation by SHBG. The metabolic relationships among these molecules are detailed in Figure S1. Mechanistic interrogation was conducted through an integrated multi‐platform framework that combined computational modeling, including the adverse outcome pathway (AOP) framework, molecular docking, molecular dynamics, molecular descriptors, pharmacophore modeling, biophysical validation‐surface plasmon resonance (SPR), and experimental metabolomics in vitro and in vivo, and functional assays in primary Leydig cells. This human‐to‐mechanism approach establishes a framework for linking real‐world EDC mixtures to disruptions in gonadal steroidogenesis. To enable exploration of chemical‐hormone interactions, we developed the EDC‐Hormone Explorer (https://chembio.njmu.edu.cn/edc‐hormone‐explorer.html), an interactive web resource. The overall experimental design is summarized in Figure S2.

## 2 Results

### 2.1 Basic Demographic Characteristics

The characteristics of the study population by age and sex categories are presented in Table S1. The study included 4255 individuals. EDC concentrations and detection frequencies are detailed in Tables S2–S4.

### 2.2 No Consistent Chemical Effects on Hormones or SHBG in Children

For male children, integrating linear regression with partial least squares discriminant analysis (PLS‐DA) revealed no significant associations between EDCs and gonadal hormones or SHBG in either male or female children (Table S5 and Figure S3). Meanwhile, within the association set with *p*< 0.1, a binomial test found no chemical classes with a consistent overall effect on gonadal hormones or SHBG (Figure S3A,B).

### 2.3 PAEs Negatively Regulate T and E2 in Male Adolescents

For male adolescents, the association between PAEs and T was strikingly consistent. Linear regression and PLS‐DA analyses revealed that seven PAEs, mono‐n‐butyl phthalate (MBP), mono‐benzyl phthalate (MBzP), mono‐2‐ethyl‐5‐carboxypentyl phthalate (MECPP), mono‐(2‐ethyl‐5‐hydroxyhexyl) phthalate (MEHHP), mono‐(2‐ethyl‐5‐oxohexyl) phthalate (MEOHP), mono‐2‐hydroxy‐iso‐butyl phthalate (MHiBP), and mono‐isobutyl phthalate (MiBP) were significantly negatively associated with T levels. Additionally, four PAEs (MBzP, MECPP, MEOHP, and MHiBP) were negatively associated with E2, while mono‐ethyl phthalate (MEP) was positively associated with E2. Furthermore, MEP was negatively associated with SHBG, whereas mono‐3‐hydroxy‐n‐butyl phthalate (MHBP) was positively associated with SHBG (Table S6 and Figure S4A). Binomial test results within the association set with *p*< 0.1 indicated that the *β* coefficients for T related to PAEs (100%, 9/9) were consistently negative (*p*< 0.01), and the *β* coefficients for E2 related to PAEs (90%, 9/10) were consistently negative (*p* = 0.02) (Figure S4A), indicating PAEs generally negatively regulate both T and E2 in male adolescents, especially T.

For female adolescents, linear regression and PLS‐DA analyses revealed no significant associations between EDCs and T or E2. Only MHBP and propyl paraben (PP) were positively associated with SHBG (Table S6 and Figure S4B). Additionally, binomial test results within the association set with *p*< 0.1 found no chemical classes with an overall consistent effect on gonadal hormones or SHBG (Figure S4B).

### 2.4 PAEs Impact T Biosynthesis via Cholesterol Provision in Male Adults

For male adults, the association between PAEs and T was strikingly consistent. Linear regression and PLS‐DA analyses revealed that nine PAEs were significantly and negatively associated with T levels: mono‐(2‐ethyl)‐hexyl phthalate (MEHP), mono‐(carboxyisononyl) phthalate (MCNP), mono‐(carboxyisoctyl) phthalate (MCOP), MBP, mono‐(3‐carboxypropyl) phthalate (MCPP), MiBP, MECPP, MEHHP, and MEOHP. Among these EDCs, MECPP, MCPP, MCNP, and MCOP showed significant negative associations with SHBG levels. MEHP, MBP, MiBP, and MEOHP were negatively associated with both T and TC levels, while MECPP, MCPP, MEHHP, and MEOHP were negatively associated with HDL‐C levels in addition to T levels. Furthermore, six of these nine EDCs (MEHP, MCNP, MCOP, MBP, MCPP, and MiBP) negatively associated with T were also negatively associated with LDL‐C levels (Table S7 and Figure S5A). The consistency of the association between PAEs and T, as well as various cholesterol‐containing variants (LDL‐C, HDL‐C, and TC), indicates the effects of those PAEs on T production through decreasing upstream cholesterol provision. Additionally, perfluorohexanoic acid (PFHxA) and triclocarban (TCC) exhibited positive associations with E2 levels, while benzophenone‐3 (BP‐3) and MEHP showed negative associations with E2 levels (Table S7 and Figure S5A).

Within the association set with *p*< 0.1, we performed a binomial test and observed consistent associations between EDCs and various biomarkers. The binomial test analysis revealed that the *β* coefficients for T related to PAEs (100%, 12/12) were consistently negative (*p*< 0.01). Additionally, the *β* coefficients for SHBG (100%, 10/10), HDL‐C (100%, 8/8), LDL‐C (100%, 13/13), and TC (100%, 12/12), all involved in the gonadal steroidogenic metabolic pathway, were consistently negative. This underscores the significant impact of PAEs exposure on T and its biosynthesis (Figure S5A).

### 2.5 EDCs Show Complex Steroidogenic Pathway Associations in Female Adults

Using linear regression and PLS‐DA models, we observed that in premenopausal women, two PFASs, perfluorohexane sulfonic acid (PFHxS), and n‐perfluorooctanoic acid (PFOA) and Bisphenol A (BPA) were positively correlated with T. Two PAEs (MCOP, and MCPP), perfluoromethylheptane sulfonic acid isomers (Sm‐PFOS) and bisphenol S (BPS) were negatively correlated with SHBG. No chemicals were correlated with E2. Additional associations can be found in Figure S6A and Table S8. For premenopausal women, within the association set with *p*< 0.1 for linear regression, our binomial test revealed no chemical classes with an overall consistent effect with gonadal hormones (Figure S6A).

In postmenopausal women, no correlations were found between chemicals and T. Four PAEs, including MCNP, MCOP, cyclohexane 1,2‐dicarboxylic acid monohydroxy isononyl ester (MHINCH), and MiBP, were negatively correlated with SHBG. Two PAEs (MCNP and MBzP) were positively associated with E2. Additional associations can be found in Figure S6B and Table S8. In postmenopausal women, the *β* coefficients for E2 were consistently negative for PFASs (100%, 6/6) using the binomial test, indicating a generally negative effect of PFASs on E2 after menopause (*p* = 0.03) (Figure S6B).

### 2.6 PAEs are Linked to Increased T Deficiency Risk in Male Adults

Considering that hormone levels in adult males are generally more stable compared to females and adolescents, we conducted logistic regression analyses on T and E2 abnormalities in adult males.

Integrating PLS‐DA analysis and logistic regression results, we identified several PAEs that significantly increased the risk of T deficiency: MEHP, MBP, MCPP, MiBP, MECPP, MEOHP, MEHHP, and MEP. Interestingly, MEHHP increased the risk of both abnormally high T and T deficiency. Furthermore, PFHxA significantly increased the risk of abnormally high E2 levels. TCC and BPA were associated with an increased risk of both abnormally high E2 and T levels (Table S9 and Figure S5B). The binomial test revealed a consistent pattern of associations between EDCs and gonadal hormone abnormalities (Figure S5B), indicating a significantly increased risk of T deficiency due to PAEs exposure.

### 2.7 Common EDCs Alter Male T Metabolism With Age and PAE Patterns

Overall, the effects of EDCs on gonadal hormones vary by EDC type, hormone type, and population age and sex.

**FIGURE 1:**
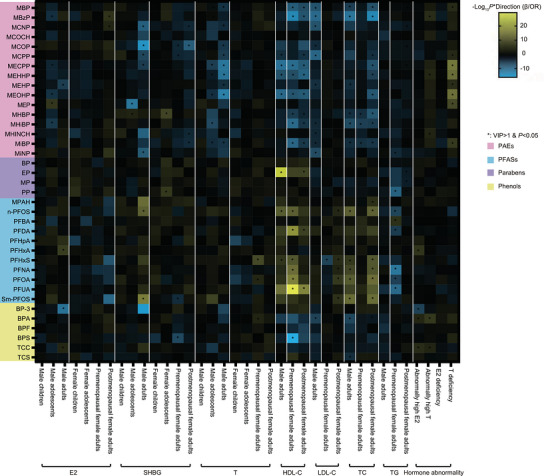
Heatmaps of linear and logistic regressions and PLS‐DA showing the associations between common EDCs and the gonadal steroidogenic metabolic pathway across male and female populations from children to adults. The gradient blue in the heat map represents *β*< 0 or OR< 1, while the gradient yellow represents *β* >0 or OR >1.“^*^” means *p*< 0.05 and variable importance in projection (VIP) >1.

First, throughout childhood to adulthood, the effect of these EDCs appears to increase in both sexes (Figure 1).

Second, PAEs consistently decreased T levels in both male adolescents and male adults, as supported by binomial test analysis (100%, 9/9, 12/12, *p*< 0.05). Notably, consistent negative associations between PAEs and various cholesterol‐containing variants (LDL‐C, HDL‐C, and TC) were also found, supported by binomial test analysis (100%, 13/13, 8/8, 12/12, *p*< 0.05), highlighting the effects of those PAEs on T production through decreased upstream cholesterol provision. Importantly, combined results from linear and logistic regression analyses suggest that MBP, MCPP, MEHP, and MiBP may collectively contribute to a simultaneous decrease in T and LDL‐C levels, potentially leading to T deficiency, also supported by binomial test analysis (*p*< 0.05) for adult males (Figure S5C). Sensitivity analysis confirms that the associations between these key chemicals with T, LDL‐C level, and T deficiency remained robust (Tables S10 and S11). The metabolic process and statistical results of PAEs in the human body are summarized in Figure S7. These findings support the hypothesis that key PAEs generally reduce T levels and contribute to T deficiency by influencing LDL‐C levels, providing a basis for mechanistic investigation. Given the consistency of these findings, our following research focused on adult males to further elucidate the specific effects and mechanisms of PAEs.

During childhood, no chemicals affecting gonadal hormones were found. In adolescence, eight PAEs were associated with T or E2 levels in males, with seven PAEs decreasing T levels; no significant associations were found in females. In adulthood, twelve EDCs (PFHxA, BP‐3, TCC, nine PAEs) affected T or E2 levels in males, with all nine PAEs decreasing T levels; in females, three EDCs (BPA, PFHxS, PFOA) increased T levels before menopause, and two EDCs (MCNP, MBzP) affected E2 levels after menopause.

### 2.8 MBP, MCPP, MEHP, MiBP Effects on Steroidogenesis: Identify Low‐T Genes

In the population studies, we identified four key chemicals (MBP, MCPP, MEHP, and MiBP) that consistently lower T and LDL‐C levels, which are the direct providers of cholesterol to Leydig cells, leading to T deficiency in male adults. To uncover the deeper mechanisms behind these effects, we developed the AOP framework (Figure S8). Notably, the phenotypes were closely tied to steroid hormone biosynthesis (Figure S9), which is crucial for adverse reproductive outcomes. These findings corroborate the impact of these PAEs on human gonadal hormones and metabolism. Therefore, our next objective was to establish an AOP framework focusing on gonadal steroidogenesis.

### 2.9 AOP Links PAEs to the NR5A1‐StAR‐CYP11A1‐CYP17A1 Testosterone Pathway

**FIGURE 2:**
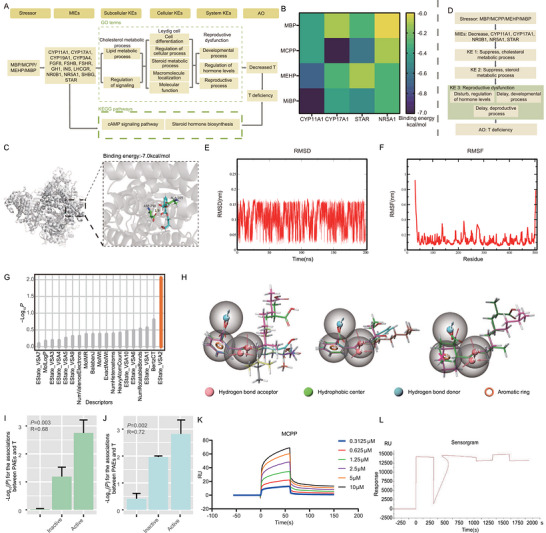
Molecular interaction and structure analysis of CYP17A1 and MCPP. (A) AOP framework from MBP, MCPP, MEHP, and MiBP to T deficiency. (B) Heatmaps of binding energies of MBP, MCPP, MEHP, and MiBP with CYP11A1, CYP17A1, StAR, and NR5A1. (C) Molecular docking results of MCPP with CYP17A1. (D) CYP11A1, CYP17A1, NR0B1, NR5A1, and StAR‐mediated AOP of T deficiency. (E) Root‐mean‐square deviation (RMSD) from molecular dynamics simulations of CYP17A1 and MCPP within 200 ns. (F) Root‐mean‐square fluctuation (RMSF) from molecular dynamics simulations of CYP17A1 with MCPP. RMSD and RMSF values indicate stable binding of CYP17A1 to MCPP in molecular dynamics simulations. (G) Correlation results of molecular descriptors with T disruption. (H) From left side to right side, the figures show nine active molecules possessing the AADR pharmacophore features (*p*< 0.05, VIP >1), five inactive molecules also possessing the AADR pharmacophore features but with suggestive effect (*p*< 0.50), and two inactive molecules partially exhibiting the AADR pharmacophore features, namely AAD, with nearly no effect (*p* >0.90), respectively. (I) Spearman correlation analysis results based on AADR feature grouping. (J) Spearman correlation analysis results based on AAADHR feature grouping. (K) Binding sensorgram between CYP17A1 and MCPP in SPR. (L) Sensorgram of CYP17A1 and the small molecule MCPP. SPR experiment demonstrates moderate affinity between CYP17A1 and MCPP.

Figure 2A illustrates the construction of an AOP framework based on hierarchical and biological relationships.

We filtered 14 Molecular Initiating Events (MIEs) and categorized these MIEs into metabolic pathways and organized Gene Ontology (GO) terms into three levels (Tables S12 and S13).

Among the 14 potential MIEs, we observed that CYP11A1, CYP17A1, NR0B1, NR5A1, and StAR play crucial roles in cholesterol metabolism, steroid metabolism, and reproductive dysfunction, ultimately leading to decreased T levels. CYP11A1 and CYP17A1 are directly engaged in steroidogenesis [16], while StAR is responsible for cholesterol transport [17], and NR0B1 and NR5A1 act as transcriptional regulators of enzymes [18]. StAR facilitates the transfer of cholesterol from the outer to the inner mitochondrial membrane [19], where CYP11A1 converts cholesterol into pregnenolone, the precursor for all steroid hormones [20]. CYP17A1 next converts pregnenolone, which is also the rate‐limiting enzyme for the formation of androgens [21]. These findings underscore the pivotal roles of CYP11A1, CYP17A1, NR0B1, NR5A1, and StAR in initiating the adverse events.

### 2.10 Molecular Docking Shows PAEs Bind NR5A1, StAR, CYP11A1, and CYP17A1

Interestingly, StAR as a transporter, CYP11A1 and CYP17A1 as metabolic enzymes, and NR5A1 as a transcription factor all have the ability to bind small molecules. Molecular docking simulations were conducted to explore the structural interactions between MBP, MCPP, MEHP, and MiBP with these proteins. Considering that NR0B1 functions as a co‐regulatory protein through heterodimerization and cannot directly bind small molecules, molecular docking studies with NR0B1 were not conducted. Figure 2B and Figures S10–S13 show that all docking binding energies are below −5 kcal/mol, indicating that these stressors exhibit good binding activity with the proteins [22]. For instance, in the analysis of CYP17A1, MCPP formed hydrogen bonds with ASP‐298, ALA‐105(*E* = −7.0 kcal/mol) (Figure 2C). MBP formed hydrogen bonds with ARG239, ASP298, and ALA302 (*E* = −6.1 kcal/mol); MEHP bound with ARG239 (*E* = −6.3 kcal/mol), and MiBP formed hydrogen bonds with GLY297, ARG239 (*E* = −6.4 kcal/mol). In the analysis of CYP11A1, hydrogen bonds were observed between VAL353 and THR354 of CYP11A1 and MBP (*E* = −6.4 kcal/mol); ARG112, VAL100, TRP108, ARG421, and ARG81 interacted with MCPP (*E* = −6.4 kcal/mol). MEHP and MiBP formed hydrogen bonds with GLN377, and TYR61, and with GLN377, LEU209, and ASN210, respectively (MEHP: *E* = −6.7 kcal/mol, MiBP: *E* = −7.0 kcal/mol). Regarding NR5A1, MBP formed a hydrogen bond with GLN339 (*E* = −6.0 kcal/mol); MCPP interacted with GLN42, ARG84, TYR99, and TYR25 (*E* = −6.1 kcal/mol); MEHP interacted with ARG84 and ALA82 (*E* = −6.3 kcal/mol), and MiBP interacted with ALA433 (*E* = −6.3 kcal/mol). Lastly, for StAR, MBP formed a hydrogen bond with ALA‐172 (*E* = −6.3 kcal/mol); MCPP interacted with ALA‐172 and ARG‐182 (*E* = −6.6 kcal/mol); MEHP interacted with ALA‐172 (*E* = −6.1 kcal/mol), and MiBP interacted with ASN‐150, ALA‐172 (*E* = −6.5 kcal/mol).

Based on this work, a preliminary AOP framework was ultimately constructed (Figure 2D). The chemicals MBP, MCPP, MEHP, and MiBP (stressors) were found to affect the expression of CYP11A1, CYP17A1, NR0B1, NR5A1, and StAR (MIE), leading to disruptions of cholesterol metabolism named key event (KE 1) and steroid metabolism (KE 2). Consequently, these disruptions resulted in reproductive dysfunction (KE 3) and ultimately decreased T production, named an adverse outcome (AO).

### 2.11 Molecular Dynamics Simulations Confirm That MCPP Targets CYP17A1

We chose MCPP and CYP17A1 for molecular dynamics simulations because CYP17A1 is the rate‐limiting enzyme for the formation of androgens with the lowest binding energy from docking. Interestingly, MCPP also showed the strongest effect with T among the four PAEs, indicating consistency between molecular docking and observed effects. After 200 ns of simulations, the RMSD data showed that the MCPP‐CYP17A1 complex stabilized (Figure 2E). RMSF analysis revealed low flexibility of the protein structure, with values mostly below 0.2 nm, indicating high stability, including near the ligand‐binding site (Figure 2F). Hydrogen bonds are crucial in protein‐ligand interactions, impacting binding strength and providing insights into biological processes, disease mechanisms, and mutation effects [23]. Hydrogen bond quantification demonstrated stable binding with MCPP, maintaining two or more hydrogen bonds with CYP17A1 (Figure S14). The motion trajectory of MCPP and CYP17A1 was shown in Movie S1.

We performed a comprehensive thermodynamic analysis of the MCPP‐CYP17A1 complex using the molecular mechanics/Poisson‐Boltzmann surface area (MM/PBSA) method, revealing a binding free energy (ΔG) of −89.276 kJ/mol, indicating stable, tight binding. The enthalpy change (ΔH) was −97.725 kJ/mol, while the entropy change (−TΔS) was 8.450 kJ/mol, showing that binding is mainly driven by favorable enthalpy. Van der Waals interactions (VDW) contributed −139.269 kJ/mol, dominating the binding energy, while coulombic interactions (COU) contributed −41.969 kJ/mol (Table S14).

### 2.12 Weight of Evidence (WoE) for the AOP Exhibits a Relatively High Confidence Level

The essentiality of the potential Key Events (KEs) was rated as “moderate” to “high” based on both experimental and epidemiological evidence. For the Key Event Relationships (KERs), biological plausibility received a “high” rating due to its consistency with established biological knowledge, while empirical support for the KERs was also rated “high”, given evidence of dose‐ and time‐dependent relationships between adjacent KEs (Table S15). The applicability of this AOP spans various biological contexts, including sex, life stage, etc. In summary, this AOP, mediated by CYP11A1, CYP17A1, NR0B1, NR5A1, and StAR, exhibits a relatively high confidence level and can be utilized to assess the impact of chemical exposures (MBP, MCPP, MEHP, and MiBP) on T reduction. We then assessed the WoE for the AOP according to the OECD handbook. The WoE exhibits a relatively high confidence level and is detailed in the Tables S15 and S16.

### 2.13 Modeling Identifies EState_VSA2's Key Role in PAE‐Related Male T Effects

To identify the key structural determinants for deeper insights into the mechanisms, we calculated molecular descriptors for PAEs, including Mass/Quantitative Descriptors, Structural Feature Descriptors, Physical‐Chemical Property Descriptors, and Electronic State Surface Area Descriptors, to evaluate their relationships with effects on T levels. As shown in Table S17 and Figure 2G, EState_VSA2 may play a major role in the effect of PAEs on T (*β* = 0.002, *p* = 0.01). Notably, among the 16 PAEs analyzed, MCPP exhibited the highest EState_VSA2 value, indicating consistency between molecular docking, molecular descriptor modeling, and observed effects.

### 2.14 Pharmacophore Modeling Links AADR, AAADHR to PAE Effects on T

As complementary tools for molecular descriptor modeling, providing the spatial arrangement of crucial functional groups, we characterized the association of T with PAEs in adult males through pharmacophore modeling and clustering (Figure 2H and Figure S15). AADR features were first identified as key for the dose (structural matching level) ‐activity (association intensity with T) dependent relationship. Cluster analysis showed that nine active molecules (*p*< 0.05, VIP >1) shared the four AADR features, while five inactive molecules met these features but with a suggestive effect (Figure 2H). Notably, the remaining two inactive molecules, cyclohexane‐1,2‐dicarboxylic acid‐mono(carboxyoctyl) ester phthalate (MCOCH) and MHINCH, did not fully fit these features with nearly no effect (Figure 2H). Subsequent Spearman analysis revealed a significant positive correlation between the AADR characteristics matching level and the intensity of EDCs' association with T (Figure 2I).

Second, AAADHR features were identified as another key for the dose‐activity dependent relationship. AAADHR showed that eight active molecules shared these features (*p*< 0.05, VIP >1) (Figure S15A), with two inactive molecules also matching but with a suggestive effect (*p*< 0.05, VIP< 1) (Figure S15B), and five not fully matching lacked effect (*p* >0.09) (Figure S15C,D). Spearman analysis also revealed a significant positive correlation between AAADHR characteristics matching level and the intensity of EDCs' association with T (Figure 2J). Interestingly, as shown in Figure S15E, MCPP, excluded from the active molecules clustering results, likely has unique structural features, including one fewer hydrophobic center compared to other significant PAEs. The ester bond and carboxyl group near the H6 position on the MCPP molecule are hydrophilic, and a few carbon atoms in the middle make MCPP less hydrophobic than other molecules.

### 2.15 SPR Experiments Validate the Binding Between MCPP and CYP17A1

To further validate and assess the binding affinity between MCPP and CYP17A1, we conducted a SPR measurement in this study. The total amount of CYP17A1 protein coupled was 12 967 RU, with the coupling sensorgram shown in Figure 2K. Figure 2L presents the steady‐state analysis results, illustrating the relationship between the RU at equilibrium and the MCPP concentration. By fitting this curve, we determined a dissociation constant (KD) of 1.65 × 10^−^^6^ M for the interaction between MCPP and CYP17A1. This KD value, falling within the range of 10^−^^6^ M, indicates a moderate binding affinity between MCPP and CYP17A1. The results from the SPR experiment provide robust evidence for the direct interaction between MCPP and CYP17A1.

### 2.16 Cell and Supernatant Metabolomics Reveal MCPP Primarily Disrupts In Vitro T Biosynthesis

**FIGURE 3:**
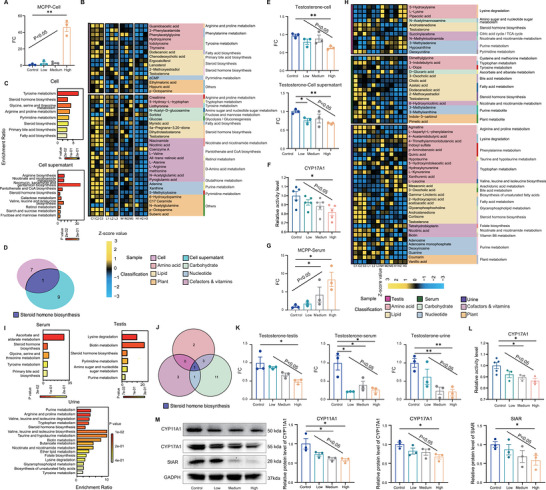
Results of in vitro and in vivo experiments. (A) MCPP showed a dose‐response relationship in each group (*p*< 0.05), indicating successful modeling in vitro. (B) Heatmap illustrating differential metabolites between the control and MCPP‐treated groups. (C) Enrichment analysis of differential metabolites between the control and MCPP‐treated groups in cells and supernatants, respectively. (D) The intersection of enriched pathways in cells and supernatants in vitro. The results showed that the overlapping pathway was steroid hormone biosynthesis. (E) Levels of T in different groups of cell and supernatant samples. With increasing MCPP, T levels showed a negative dose‐response relationship. (F) The activity of CYP17A1 dose‐dependently decreased with increasing concentrations in vitro. (G) MCPP showed a dose‐response relationship in each group (*p*< 0.05), indicating successful modeling in vivo. (H) Heatmap illustrating differential metabolites between the control and MCPP‐treated groups. (I) Enrichment analysis of differential metabolites between the control and MCPP‐treated groups in testis, serum, and urine, respectively. (J) The intersection of enriched pathways in testicular, serum, and urine enrichment pathways in vivo. The results showed that the overlapping pathway was steroid hormone biosynthesis. (K) Levels of T in different groups of testis, serum, and urine samples. With increasing MCPP, T levels showed a negative dose‐response relationship (*p*< 0.05). (L) The activity of CYP17A1 decreased with increasing concentrations in vivo. (M) Relative protein levels of CYP11A1, CYP17A1, and StAR decreased with increasing concentrations. In vitro: low, medium, and high: 0.01, 1, and 100 µmol/L, respectively. In vivo: low, medium, and high: 0.01, 1, and 100 mg/kg, respectively. Data are shown as mean ± SD. “^*^” indicates *p*< 0.05, “^**^” indicates *p*< 0.01.

MCPP was identified as the most potent phthalate by human association studies and in silico analysis. Subsequent experiments, therefore focused on validating its effects in living systems. To determine whether MCPP suppresses testosterone biosynthesis, we assessed its impact on TM‐3 mouse Leydig cells, global metabolism, and key enzymes, including CYP11A1 and CYP17A1. CCK‐8 assays showed that MCPP at concentrations ranging from 0.01 to 100 µmol/L caused no significant damage to cell viability (Figure S16A), and MCPP concentration increased in a dose‐dependent manner in vitro (*p*< 0.05) (Figure 3A), indicating successful cell modeling.

To assess the major metabolic pathways affected, we performed metabolomic analysis of cells and supernatants. Orthogonal partial least squares discriminant analysis (OPLS‐DA) demonstrated an evident separation between control and MCPP‐treated groups, with Q2 (cum) values exceeding 0.4, indicating the validity of the models (Figure S16B). A total of 16 and 28 differential metabolites were identified in cells and supernatants, respectively (Figure 3B). Importantly, pathway enrichment analysis revealed that metabolite differences between the control and MCPP‐treated groups in both cells and supernatants were enriched in the steroid hormone biosynthesis pathway, indicating that steroid hormone disruption is the major metabolic perturbation caused by MCPP from a global metabolic perspective (Figure 3C,D). T levels were significantly reduced in a dose‐dependent manner following MCPP exposure in both cells and supernatants (*p*< 0.05) (Figure 3E), verifying the suppressive effect of MCPP on T. Enzymatic assays further revealed marked dose‐dependent inhibition of CYP11A1 and CYP17A1 activity by MCPP (Figure 3F and Figure S16D), consistent with the direct binding observed in SPR (Figure 2K,L).

### 2.17 In Vivo Metabolomics Show MCPP Inhibits T via NR5A1‐StAR‐CYP11A1‐CYP17A1 Pathway

To corroborate the in vitro findings, an MCPP exposure model as an internal exposure biomarker was established. Serum testing revealed a dose‐dependent increase in MCPP levels, confirming successful internal exposure to MCPP in the animal model (Figure 3G). To assess the major metabolic pathways affected, we performed metabolomic analysis of testis, serum, and urine. The OPLS‐DA models showed distinct metabolic profiles in testis, serum, and urine with Q2 (cum) >0.4, indicating the validity of the models (Figure S16C). In the mouse testis, 11 metabolites were altered in the MCPP‐treated group compared with controls. There were 15 altered metabolites in serum and 31 in urine (Figure 3H). Notably, similar to the in vitro study, pathway enrichment analysis revealed that metabolite differences in testis, serum, and urine were enriched in the steroid hormone biosynthesis pathway, indicating the pivotal role of steroid hormone disruption by MCPP (Figure 3I,J). T levels in testis, serum, and urine were significantly reduced in a dose‐dependent manner following MCPP exposure (Figure 3K). Enzymatic assays further revealed marked inhibition of CYP11A1 and CYP17A1 activity (Figure 3L and Figure S16E). To further examine NR5A1 involvement and its downstream pathway alterations, we measured the mRNA expression levels of Star, Cyp11a1, and Cyp17a1 in the mouse testis. The results showed significant transcriptional suppression of these genes, indicating reduced NR5A1 activity (Figure S16F). Western blot analysis also confirmed that CYP11A1, CYP17A1, and StAR protein levels were dose‐dependently reduced (Figure 3M), indicating that transcriptional and protein‐level inhibition synergistically impair testosterone biosynthesis.

### 2.18 Leydig Model Confirms MCPP Inhibits T by Binding near Iron‐Protoporphyrin IX

**FIGURE 4:**
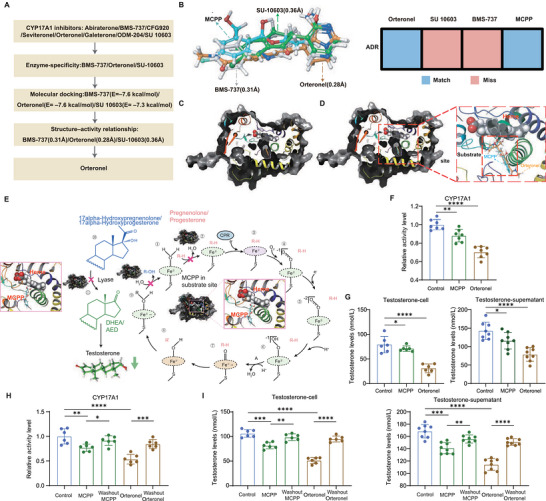
Inhibition of T synthesis by MCPP binding to the active site. (A) Screening process for positive inhibitors of CYP17A1. (B) Structure–activity relationship (SAR) analysis of inhibitors. White: BMS‐737; orange: Orteronel; pink: SU‐10603; green: MCPP. (C) Molecular docking analysis of Orteronel with CYP17A1. (D) Molecular docking analysis of MCPP and Orteronel with CYP17A1. (E) Schematic diagram of the involvement of CYP17A1 in the catalytic action of the testosterone synthesis pathway. (F) Decreased CYP17A1 activity upon exposure to MCPP and Orteronel. (G) T levels in cells and supernatants from different treatment groups exposed to MCPP and Orteronel. (H) CYP17A1 activity in Leydig cells after exposure and washout treatment with MCPP or Orteronel, showing significant inhibition by both compounds and recovery after washout. (I) Intracellular and secreted T levels in Leydig cells across control, exposure, and washout groups, indicating marked reduction upon exposure and substantial restoration following washout. Data shown as mean ± SD. “^*^” means *p*< 0.05, “^**^” means *p*< 0.01, “^***^” means *p*< 0.001, “^****^” means *p*< 0.0001.

To precisely elucidate the CYP17A1‐MCPP interaction mechanism in living organisms, we referenced and selected the representative dose from our preceding in vitro multi‐dose investigation, along with known CYP17A1 inhibitors according to previous studies [24], for further exploration of the specific molecular target. We screened a set of known CYP17A1 inhibitors from the PubChem database, including Abiraterone, BMS‐737, CFG920, Seviteronel, Orteronel, Galeterone, ODM‐204, and SU‐10603. To minimize potential confounding effects arising from cortisol suppression and subsequent hormonal compensation, broad‐spectrum enzyme inhibitors and compounds with poor enzymatic selectivity were excluded. Consequently, three relatively selective candidates—BMS‐737, Orteronel, and SU‐10603 were retained for further analysis (Figure 4A and Table S18).

Molecular docking revealed that all three inhibitors could stably bind to the catalytic pocket of CYP17A1, with comparable binding affinities (BMS‐737: −7.6 kcal/mol, Orteronel: −7.6 kcal/mol, SU‐10603: −7.3 kcal/mol). To identify the inhibitor with the most similar binding mode to MCPP, structure‐activity relationship (SAR) analysis was conducted. Comparative analyses indicated that Orteronel exhibits a highly similar molecular profile to MCPP in terms of the key pharmacophore structural feature (ADR), whereas SU‐10603 and BMS‐737 showed lower structural similarity in these aspects (Figure 4B). The degree of structural superposition between each inhibitor and MCPP was further evaluated based on RMSD (BMS‐737: 0.31 Å, Orteronel: 0.28 Å, SU‐10603: 0.36 Å). These findings indicate that Orteronel exhibits the highest structural similarity to MCPP. Therefore, Orteronel was selected as the positive control inhibitor.

As illustrated in Figure 2C, MCPP tightly binds within the catalytic pocket of CYP17A1 through hydrogen bonds, specifically at the substrate‐binding site adjacent to the iron‐containing Protoporphyrin IX (heme). Notably, Orteronel was also found to bind at the same active site (Figure 4C), stably occupying the substrate‐binding site through coordination bonds with the heme, exhibiting a binding conformation highly similar to that of MCPP (Figure 4D). Interestingly, all substrates (Pregnenolone, Progesterone, 17alpha‐Hydroxypregnenolone, and 17alpha‐Hydroxyprogesterone) required for the catalytic involvement of CYP17A1 in T production require binding to this site for the next reaction. This site plays a dual catalytic role in CYP17A1 function, mediating electron transfer and oxygen activation. During testosterone biosynthesis, the heme iron is first reduced through interactions with electron donors, enabling molecular oxygen binding and subsequent formation of a highly reactive iron–oxo intermediate. This intermediate sequentially catalyzes the C17 hydroxylation and C17–C20 bond cleavage of steroid substrates, yielding androstenedione and dehydroepiandrosterone, key precursors of T. Both MCPP and Orteronel compete with endogenous substrates for this active site, thereby blocking the catalytic reaction and ultimately suppressing T production (Figure 4E).

To further investigate the cellular targets of MCPP in murine steroidogenesis, we isolated and cultured mouse testicular Leydig cells and examined the shared mechanism of MCPP and Orteronel at the primary cell level. The results demonstrated that both compounds significantly reduced CYP17A1 enzymatic activity (Figure 4F), accompanied by a marked decrease in both intracellular and secreted T levels (Figure 4G). Notably, enzymatic activity and T production were substantially restored following compound washout, indicating that MCPP's inhibitory effect is reversible and not attributable to overt cytotoxicity. Specifically, CYP17A1 activity returned to near‐baseline levels (Figure 4H), and T concentrations markedly increased during the recovery phase (Figure 4I), confirming the functional reversibility of MCPP‐induced inhibition. Importantly, the shared inhibitory pattern with Orteronel and the recovery upon washout collectively demonstrate that MCPP does not act through general cellular toxicity but by transiently occupying the CYP17A1 catalytic pocket. This target‐specific and reversible interaction provides the identification of precise molecular initiating events linking MCPP chemical structure to functional disruption. To enable systematic exploration of the interaction landscape, we provide an interactive web resource (EDC‐Hormone Explorer; https://chembio.njmu.edu.cn/edc‐hormone‐explorer.html), with details in the Supplementary Materials (Figures S17 and S18).

## 3 Discussion

Evidence from nationally representative cohorts, including NHANES, indicates that EDCs impact gonadal hormones, yet estimates vary across populations and study designs, and a hormone‐centric focus has left upstream metabolic regulation and mechanisms insufficiently resolved [25]. Addressing this gap at a population scale is essential given the ubiquity of synthetic chemical mixtures and their relevance to reproductive and metabolic health. By providing a landscape‐level view that spans mixed exposure to common EDCs across age and sex strata and key components of the gonadal steroidogenic pathway, our study moves beyond single‐chemical associations to a framework that prioritizes generalizability (U.S. nationally representative data) and biological interpretability (linkage to molecular initiating events). This approach aligns the endocrine disruption question with other major population health drivers routinely interrogated in national cohorts (e.g., obesity and mortality [13]), while directly informing risk prioritization for emergent chemical mixtures.

**FIGURE 5:**
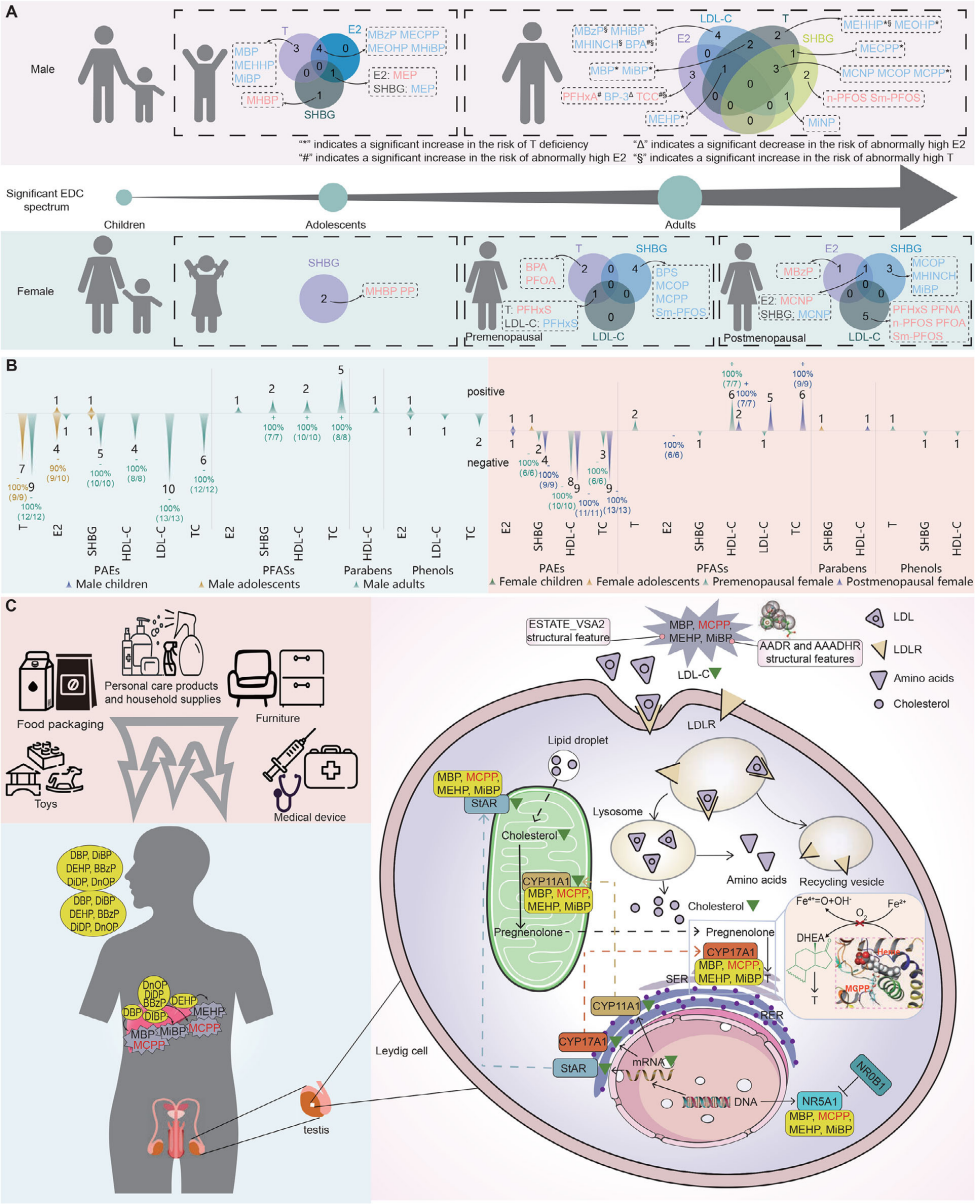
The summary of this study. (A) The landscape for associations between mixed exposure to EDCs and molecules in the gonadal steroidogenic metabolic pathway across ages and sexes. Blue indicates negative correlations, and red indicates positive correlations (*p*< 0.05 and VIP >1). (B) The summary of the association landscape based on types of EDCs. The bar chart illustrates the number of significant associations (*p*< 0.05 and VIP >1) based on types of EDCs. The upper part of each bar represents the number of positive associations, while the lower part represents the number of negative associations. The data in brackets are significant results from binomial tests (*p*< 0.05), where linear regression results with *p*< 0.1 were selected. (C) The mechanisms of PAEs‐induced T decrease in adult males through decreasing upstream cholesterol provision by LDL‐C and binding proteins in the NR5A1‐StAR‐CYP11A1‐CYP17A1 T biosynthesis pathway, depending on their EState_VSA2, AADR, and AAADHR structural features. MCPP, highlighted as a new concern, was further validated by SPR and in vitro/in vivo metabolomics, demonstrating that it inhibits testosterone synthesis by binding to the iron‐containing protoporphyrin IX. The green arrow indicates a decrease in activities and levels. RER: Rough Endoplasmic Reticulum; SER: Smooth Endoplasmic Reticulum.

Our study provides evidence that EDC effects increase from childhood to adulthood in both sexes (Figure 5A).

For intrinsic factors, there are age‐related changes in metabolism and hormonal regulation, which affect individual susceptibility to environmental stressors [26]. We also note that the prevalence of chronic diseases among adults is 21.43%, and it increases with age [27]. For external factors, adults face higher cumulative risks due to prolonged EDC exposure, leading to more significant adverse effects on endocrine and reproductive health, consistent with previous research [28]. Moreover, lifestyle‐related risk factors like smoking, alcohol consumption, and obesity can interact with EDCs and exacerbate these effects in adults [29, 30]. These findings emphasize that adult populations may face greater reproductive risks from real‐world EDC mixtures.

Previous studies mostly focused on DEHP and its metabolites’ effects on gonadal hormone levels in humans [31, 32], and their results echo our findings. Regarding the gonadal steroidogenic metabolic pathway, effective cholesterol delivery from LDL‐C to Leydig cells is therefore crucial for maintaining optimal levels of T. Moreover, our study identified a negative correlation between MEHP, MCPP, MiBP, and MBP and LDL‐C levels as well as T, aligning with prior studies [32, 33, 34], indicating that PAEs may disrupt cholesterol metabolism and impact T synthesis. Therefore, our findings indicate that, among common EDCs under mixed exposure, various PAEs are robustly negatively correlated with T levels in adolescents and T levels as well as cholesterol transport supporting T biosynthesis in adults from a landscape view (Figure 5B), advancing our knowledge in this area.

Notably, among PAEs, we highlighted the negative association between MCPP and both T and LDL‐C levels, which shows the strongest associations and is consistent with the following unbiased molecular docking and molecular descriptor modeling results with structure‐based mechanisms. MCPP can arise as a secondary metabolite of multiple phthalates such as di‐isodecylphthalate (DiDP), di‐butyl phthalate (DBP), benzyl butyl phthalate (BBzP), and di‐n‐octyl phthalate (DnOP), (Figure S7). Current research on MCPP primarily focuses on prenatal exposure and neonatal metabolic dysfunction [35]. Its impact on human health is less studied compared to other PAEs, including endocrine disruption and developmental effects. Our study arises concern about MCPP's broader impact on adult male gonadal function.

EState_VSA2 is defined as the sum of the van der Waals surface area (VSA) of atoms whose property *X* lies in the range *Y* [36]. In particular, EState_VSA2, which relates to the distribution of electrons, is calculated using the electrotopological state index (EState) method [37]. Notably, MCPP has the biggest value of EState_VSA2, which is consistent with its strongest effects observed in humans and the lowest docking binding energy with target proteins. In pharmacophore modeling, key features such as hydrogen bond acceptors (A), hydrophobic centers (H), aromatic rings (R), and hydrogen bond donors (D) play significant roles in understanding how chemicals interact with biological targets. Furthermore, our 3D pharmacophore analysis highlighted that the AADR and AAADHR features dose‐dependently affect the activity disrupting gonadal steroidogenesis. Moreover, the absence of a hydrophobic group in MCPP is significant for its increased disruptive potential, potentially due to enhanced hydrophilicity. Therefore, these descriptors are useful tools for predicting the endocrine‐disrupting effects of PAEs and designing safer alternatives.

The PAEs included MBP, MCPP, MEHP, and MiBP, and their target genes are linked to the StAR‐CYP11A1‐CYP17A1‐T biosynthesis pathway. DEHP, DBP, and their metabolites reduced these genes’ expression in vitro [38, 39]. Interestingly, DBP also decreased the activity of CYP11A1 and CYP17A1 in vitro [40]. Therefore, although these proteins’ involvement in some PAEs‐induced gonadal steroidogenesis disruption has been noticed, actual interactions between these proteins and PAEs are largely unknown. Interestingly, StAR, CYP11A1, CYP17A1, and NR5A1 all have the ability to bind small molecules with structure similarity to PAEs, driving us to conduct chemical‐protein binding tests, which can also explain the reported activity reduction caused by DBP [40]. Our molecular docking and subsequent molecular dynamics simulations confirmed that these PAEs may disrupt T synthesis by binding to NR5A1, StAR, CYP11A1, and CYP17A1 proteins, affecting these key proteins’ function and leading to T deficiency in male adults (Figure 5C).

MCPP was further validated by SPR and in vitro/in vivo metabolomics, demonstrating that it inhibits testosterone synthesis by binding to the iron‐containing protoporphyrin IX, a critical catalytic center. Our docking and validation results reveal that MCPP and structurally related phthalates engage the CYP17A1 active site in a manner reminiscent of pharmacological inhibitors. Recent structure‐based inhibitor studies [41, 42] highlight the druggability and structural plasticity of this catalytic pocket. Our findings extend this paradigm by demonstrating that environmental chemicals can exploit similar structural vulnerabilities, raising opportunities for predictive screening. These insights offer a structure–metabolome–informed screening strategy for endocrine disruptors and support regulatory prioritization of high‐risk phthalate metabolites like MCPP. The collective mechanisms can be found in Figure 5C.

In our study, PFHxA was positively associated with E2 levels in male adults, while PFHxS and PFOA correlated with increased T in premenopausal women. Prenatal exposure to PFAS mixtures was positively related to E2 in male infants [43], while serum T was positively associated with PFOA, PFHxS, perfluorononanoic acid (PFNA), and the sum of PFASs among control adult females [44]. In mice, PFHxS exposure has been linked to a prolonged estrus cycle and reduced ovulation rates [45], inhibiting kisspeptin expression and affecting reproductive functions [46]. Our study provides new information on PFASs' effects on gonadal hormones in the specific population. However, the limited data on PFASs' effects on reproductive health highlight the need for further research.

In our study, exposure to TCC and BP‐3 was found to have positive and negative associations with E2 levels in male adults, respectively. BPA was associated with increased T levels in premenopausal women. A Polish study also found that higher BPA levels were associated with increased T in women with PCOS [47]. Our study adds new insights into the effects of phenols on gonadal hormones in these specific populations and underscores the need for further research.

SHBG plays a vital role in the transport of gonadal hormones [48], which is crucial for hormone balance. The relationship between SHBG and various chemicals varies by demographic factors, as shown in our landscape (Figure 5A). Given the liver's pivotal role in SHBG synthesis [49], these findings suggest that chemicals affecting SHBG might lead to liver dysfunction and damage, impacting liver metabolism and thereby affecting gonadal hormone transportation.

Several limitations should be noted. The population‐based analysis was cross‐sectional; therefore, temporality and causality cannot be established. In addition, EDC concentrations and endogenous biomarkers were measured in single‐time‐point biospecimens, which may not fully reflect long‐term exposure or temporal variability. Residual confounding also cannot be fully excluded. To partially address these issues, we adjusted for major demographic and lifestyle covariates and performed stratified analyses by age, sex, and menopausal status. We also integrated AOP, molecular docking, molecular dynamics, SPR, in vivo and in vitro metabolomics, and Leydig‐cell functional experiments to provide biological plausibility for the prioritized associations. Future prospective studies with repeated exposure measurements are still needed to better assess causal relationships. Meanwhile, although the mechanistic validation in this study focused on male‐related testosterone biosynthesis because the most consistent population signal was observed for PAEs and lower T in adult males, the epidemiological analyses also suggested female‐specific and menopause‐dependent association patterns (Figure 5A,B). These heterogeneous findings may reflect differences in ovarian steroidogenic activity, menopausal endocrine milieu, and SHBG‐mediated hormone transport. Previous studies have reported that EDCs can affect ovarian folliculogenesis and steroidogenesis, partly through changes in steroidogenic enzymes and ovarian hormone production [50, 51]. Future studies using female animal models are needed to examine these female‐specific mechanisms.

This study establishes a population‐to‐mechanism framework for mapping the mixed effects of EDCs on gonadal steroidogenesis using one of the largest U.S. nationally representative samples. By integrating large‐scale human data with in silico structural modeling, biophysical validation (SPR), in vitro/in vivo metabolomics, and functional validation, we establish a multi‐level evidence chain that links real‐world exposures to molecular initiating events. We identify MCPP as a previously under‐recognized phthalate metabolite of concern, showing the strongest disruption of the NR5A1–StAR–CYP11A1–CYP17A1 pathway via direct binding to the iron‐containing protoporphyrin IX catalytic center of CYP17A1. Beyond this structural mechanism, we show that EDC mixtures consistently impair testosterone biosynthesis, with effects strengthened with age and differing by sex—features that elevate mixture exposures to a population‐relevant, policy‐salient concern.

## 4 Methods

### 4.1 Study Design and Population

This study used the National Health and Nutrition Examination Survey (NHANES) 2013–2014 and 2015–2016 data (20,146 participants), focusing on male and female children (6–11years), adolescents (12–19 years), and adults (≥20 years, including pre‐ and postmenopausal women). Of these, 4255 met the inclusion criteria for analysis (Figure S19). The survey is approved by the National Center for Health Statistics Ethics Review Board, with informed consent from all participants.

### 4.2 Measurements of Serum and Urine Samples for EDCs and Endogenous Chemicals

The study measured EDCs such as PAEs, PFASs, phenols, and parabens (full names and structures in Table S19) and endogenous chemicals including T, E2, SHBG, TC, TG, LDL‐C, and HDL‐C. The 37 chemicals included in this study were selected based on the data availability, their relevance as commonly encountered endocrine‐disrupting chemicals, and their coverage of major chemical classes of public health concern, including phenols, parabens, PAEs, and PFASs. Urine and serum samples were collected at the examination center per guidelines, with analytical methods detailed in the Supporting Information.

### 4.3 Construction of the AOP Framework for Studying Mechanisms

#### 4.3.1 Identification of Potential MIEs

Comparative Toxicogenomics Database (CTD, https://ctdbase.org/) and GeneCards (https://www.genecards.org/) were used. The methods are detailed in the Supporting Information.

#### 4.3.2 Identification of KEs

Phenotypes related to exogenous chemicals were gathered from the CTD's “GO” and “Pathways” tabs using keywords such as “mono‐(2‐ethylhexyl) phthalate”, “mono‐(3‐carboxypropyl) phthalate”, “mono‐isobutyl phthalate”, and “monobutyl phthalate”. Concurrently, Gene Ontology (GO) enrichment and Kyoto Encyclopedia of Genes and Genomes (KEGG) pathway annotations for potential MIEs were conducted with the R package “clusterProfiler” to link exogenous chemicals to decreased T levels. Intersections of CTD‐retrieved and R‐annotated phenotypes were filtered through empirical evidence and literature, identifying KEs closely associated with T levels.

#### 4.3.3 Construction of the AOP Framework

GO terms associated with potential KEs were manually searched in the AmiGO 2 database (https://amigo.geneontology.org/amigo) to examine the ancestor chart and categorize them into different levels of KEs. Simultaneously, genes defined as MIEs were queried in KEGG to elucidate the involved metabolic pathways. An AOP framework was then constructed based on the hierarchical and biological relationships among these elements [52].

### 4.4 Molecular Docking

In silico molecular docking was conducted to examine interactions between exogenous chemicals and MIEs. AutoDock Vina 1.1.2. software (https://vina.scripps.edu/) was used [53]. The methods are detailed in the Supporting Information.

### 4.5 Molecular Dynamics Simulation

Acpype computed small molecule charges and topology. Using Gromacs 2023, the complex was placed in a cubic box with TIP3P water and Na^+^ and Cl^−^ ions for electroneutrality [54]. Energy minimization with steepest descent and a 1000 kJ·mol^−1^·nm^−1^ threshold was performed to achieve a reasonable level of system energy and atomic forces. Equilibrium simulations under the NVT ensemble used a 2‐fs step size for 100 ps, with V‐rescale temperature coupling at 300 K [55]. LINCS constrained bond lengths, and a 1000 kJ·mol^−1^·nm^−2^ restraint was applied to protein heavy atoms. Energy and conformations were saved every 500 steps. Molecular dynamics simulations of the protein‐ligand complex were run for 200 ns with a 2‐fs step size, maintaining 300 K and 1 atm. Consistent interaction handling and parameters from earlier stages were used. Energy data and trajectory files were recorded and compressed every 5000 steps.

We evaluated the conformational stability of the protein‐ligand binding mode by calculating the RMSD of protein backbone atoms and ligand molecules. We assessed the protein structure's rigidity and flexibility by computing the RMSF of protein residues. Additionally, we quantified hydrogen bonds between proteins and ligands and analyzed binding frequency fluctuations to study key interaction dynamics.

### 4.6 Estimation of Binding Free Energy Using the MM/PBSA Method

MM/PBSA calculations were performed using the gmx_mmpbsa tool to estimate the protein‐ligand binding free energy. We extracted 100 frames from the last 10 ns of the simulation, spaced 100 ps apart, for single‐point energy and solvation analysis. Gas‐phase internal energy was computed with the AMBER force field, while van der Waals and electrostatic interactions were determined using LJ potentials and Coulomb's law. Solvation free energy was split into polar (via the PB equation with specific dielectric constants) and non‐polar components (using the SASA model and a surface tension of γ = 0.0072 kcal·mol^−1^·Å^−2^). Binding free energy (ΔGbind) was calculated using the following formula:

(1) ΔGbind=ΔGcomplex−ΔGreceptor−ΔGligand

(2) $$\Delta GX=\Delta EMM+\Delta GPB+\Delta GSA+\left(-\;T\Delta S\right)$$

Here, ΔEMM includes gas‐phase molecular mechanics energy; ΔGPB and ΔGSA are polar and non‐polar solvation free energies, respectively; and −TΔS accounts for conformational entropy changes. The statistical average of 100 frames was used to correct for conformational entropy effects. Finally, we performed statistical analysis of the binding free energy, and the inhibition constant (Ki) was estimated from ΔGbind, using the relation Ki = exp(ΔGbind/RT), to assess the relative inhibitory potential of the ligand rather than for absolute quantitative prediction.

### 4.7 Assessment of the AOP Framework

WoE for the overall AOP served as the basis for its application in risk assessment, priority settings, or testing strategies. The methods are detailed in the Supporting Information.

### 4.8 Molecular Descriptor Modeling

In this study, for key findings in adult males, 2D structure SDF files for 16 PAEs were obtained from the PubChem (https://pubchem.ncbi.nlm.nih.gov/), and analyzed using the RDKit Descriptor Calculator in ChemDes (https://www.scbdd.com/chemdes/). Descriptors with more than 80% semi‐constant values were excluded to improve statistical power [56]. We then conducted a correlation analysis between these descriptors and their effects on T, providing insights into how PAEs’ structural characteristics relate to effects. Effects on T were defined based on significant associations, with non‐significant associations assigned a value of 0 [57].

### 4.9 Pharmacophore Modeling and Cluster Analysis

Small molecules were divided into active (*p*< 0.05, VIP >1) and inactive sets. The active molecule set was initially clustered and analyzed for common pharmacophore features, such as hydrogen bond donors, hydrophobic regions, and aromatic rings. Then, the pharmacophore models of the inactive and active molecule sets were matched and analyzed to assess whether the inactive molecule set possessed the key features of the active molecule set and their degree of matching, revealing differences between the molecules at the 3D structural level and important pharmacophore features. To investigate the Spearman relationship between pharmacophore features and the −log_10_(*P*) values of EDCs' association with T, we defined 0, 1, and 2 based on pharmacophore matching levels.

### 4.10 Verification of Binding Affinity by SPR Experiments

The Biacore 8K system was employed for real‐time binding interaction studies between MCPP (TM standard Co., Ltd., 722564) and CYP17A1 (Feiyuebio Co, Ltd., FY‐523442). In this experiment, the CM5 chip was activated through the amino coupling method. 1‐Ethyl‐3‐(3‐dimethylaminopropyl) carbodiimide (EDC, Cytiva) and N‐hydroxysuccinimide (NHS, Cytiva) were utilized to fix the chip, and channel 4 of the chip was activated at a flow rate of 10 µL/min. The concentration of CYP17A1 was 50 µg/mL, and the protein was immobilized on channel 4 of the chip at a flow rate of 10 µL/min to generate a coupling graph. The channel was blocked with ethanolamine at a flow rate of 10 µL/min. Channel 3 was taken as the reference. MCPP was diluted into several concentrations in a 96‐well plate and coupled with the target protein via the chip from low to high concentrations. The flow rate was 30 µL/min, and the duration was 150 s. After the flow of each concentration point, the chip was regenerated with a 10 mM glycine hydrochloride (pH 2.0) solution for 5 min. This process was repeated until all the corresponding concentrations of the analytes were completed. The data were globally fitted to a 1:1 Langmuir binding model by using the Biacore Insight evaluation software (Cytiva, Marlborough, MA, USA) to obtain the binding and dissociation constants (KD values), typically within the concentration range of 10^−3^–10^−9^ M. These values indicate the strength of binding between the protein and the small molecule: a KD value of 10^−3^ M suggests relatively weak binding, approximately 10^−6^ M indicates moderate binding, and around 10^−9^ M indicates strong binding [58]. Most antibodies exhibit KD values in the low micromolar (10^−6^) to nanomolar (10^−7^–10^−9^) range.

### 4.11 In Vivo and In Vitro Experiments

#### 4.11.1 In Vivo Studies in Mice

Adult male C57 mice (6–8 weeks of age) were used in this study [59]; the mice were purchased from Nanjing Medical University. All animal experimental procedures strictly follow the experimental animal care and use guidelines of Nanjing Medical University, and have been approved by the Animal Ethics Committee of Nanjing Medical University (IACUC‐2411069). The mice were kept in standard cages with an ambient temperature of 222°C ± 2°C, humidity of 50% ± 15%, and a day‐night cycle of 12 h of light /12 h of darkness. Mice were provided with standard feed and clean water. After one week of adaptive feeding, the mice were randomly divided into four groups according to body weight: 6 mice in the control group and 4 mice in each group in the experimental group. Except for the control group, mice in other groups received DnOP (parent chemical of MCPP) by gavage treatment [60, 61]. The doses were 0.01, 1, and 100 mg/kg, respectively, and the drug was dissolved in corn oil. Control mice were given common corn oil. Processing lasts 7 days [62]. The dose selection of DnOP is based on the daily exposure level of the population and previous literature reports. Specifically, the 0.01 mg/kg dose is within the daily exposure of U.S. adults (about 3–30 µg/kg/day) [63]; The medium dose (1 mg/kg) was set with reference to typical DnOP levels reported in environmental media (about 1 mg/kg) [64, 65]; The high dose of 100 mg/kg is close to the previous experimental design for DnOP exposure in mice [66]. Mouse serum and urine samples were collected at the end of the experiment for subsequent analysis. After the experiment, according to the relevant literature [67], we collected urine and testicular tissue samples of mice for the analysis of hormone levels.

#### 4.11.2 Cell Culture

The TM‐3 cell line (mouse Leydig cells) was cultured in DMEM/F‐12 medium containing 5% fetal bovine serum (FBS), 2.5% equine serum (HS), 100 IU/mL penicillin, and 100 µg/mL streptomycin under 37°C and 5% CO_2_, and was tested negative for mycoplasma. To ensure normal cell growth and viability, the treatment concentration of MCPP is set within a range that does not cause significant cell death. Cell‐culture experimental units were randomly assigned to treatment groups before exposure. According to the experimental design, TM3 cells were exposed to different concentrations of MCPP (0, 0.01, 1, and 100 µmol/L) for 24 h. The relevant literature shows that PAEs do not produce significant toxic effects on cells in this concentration range and aligns with previous in vitro studies on structurally similar PAEs, allowing for direct comparison of potency and biological effects [67]. Low‐dose settings represent exposure to the general population [68]. Medium represents situations of occupational exposures [69], and high dose represents large exposures in special situations [70]. After the treatment, the cells and their supernatant were collected for subsequent analysis.

#### 4.11.3 Enzyme Activity of CYP11A1 and CYP17A1

The enzyme activities of CYP11A1 and CYP17A1 in the testicular tissue and TM3 cells were measured using the mouse CYP11A1 ELISA kit and CYP17A1 ELISA kit (Yaji Biological, Shanghai, China) in accordance with the manufacturer's protocol.

#### 4.11.4 Cell Viability Assay

TM3 cells were inoculated in 96‐well plates at a density of 10 000 cells per well and cultured overnight. The cells were exposed to different concentrations of MCPP for 24 h. The survival rate of the cells in each group was then assessed using the CCK‐8 kit (C0038, Beyotime, Beijing, China).

#### 4.11.5 RNA Extraction and Real‐Time Quantitative PCR

Total RNA from testicular tissue was extracted using TRIzol reagent (R711‐01, Vazyme, Nanjing, China). cDNA was generated from 2 µg of RNA with AMV reverse transcriptase (Promega). The expression of mRNAs for the target genes was subsequently assessed using the ABI 7900 Fast Real‐Time PCR System (Applied Biosystems, Foster City, CA, USA). The primer sequences utilized are detailed in Table S20, with Gapdh serving as the internal control. The analysis of target mRNA expression was conducted using the comparative Ct method.

#### 4.11.6 Protein Extraction and Western Blotting

Total proteins from testicular tissue were extracted using a RIPA buffer that contained protease inhibitors (Complete, Roche, Basel, Switzerland). The protein concentrations were measured using the bicinchoninic acid (BCA) Protein Assay kit (E112‐02, Vazyme, Nanjing, China). Proteins were separated using 12% SDS‐polyacrylamide gel electrophoresis and electrophoretically transferred to polyvinylidene fluoride (PVDF) membranes. The membrane was then blocked in PBS buffer containing 5% bovine serum albumin for 1 h at room temperature. Transferred blots were incubated with CYP17A1 (Rabbit Polyclonal Antibody, Cat No. 14447‐1‐AP, Proteintech Group, Rosemont, IL, USA), CYP11A1 (Rabbit Polyclonal Antibody, Cat No. 13363‐1‐AP, Proteintech Group, Rosemont, IL, USA), StAR (Rabbit Monoclonal Antibody, Clone D10H12, Cell Signaling Technology, USA) at a dilution of 1:2 000 and GAPDH (Rabbit Polyclonal Antibody, Cat No. 10494‐1‐AP, Proteintech Group, Rosemont, IL, USA) at a dilution of 1:5 000 overnight at 4°C. The transferred blots were incubated with the secondary antibody horseradish peroxidase‐conjugated goat anti‐rabbit IgG (Boster, Wuhan, China) for 1 h at room temperature. Protein signals were detected using ECL solution (E412‐02, Vazyme, Nanjing, China) by gel imaging analysis system (Tanon 4600, Shanghai, China), and band intensities were quantified using ImageJ software (version 1.48).

#### 4.11.7 Metabolomics Analysis

The sample preparation method for metabolomics analysis of testicular tissue, serum, urine, TM3 cells, and cell supernatant was adapted from our previous reports [71, 72]. Briefly, testicular tissue and TM3 cells were pulverized separately, and then each sample type (testicular tissue, TM3 cells, serum, urine, and cell supernatant) was individually mixed with 80% methanol/water (v/v) and homogenized by ultrasonic disruption. After centrifugation at 16 000 × *g* for 15 min at 4°C, the supernatants were collected, dried, and reconstituted for metabolomics analysis.

Metabolomics analyses were performed on the Ultimate 3000 UPLC system (Dionex, Germering, Germany) and Q‐Exactive mass spectrometer (Thermo Fisher Scientific, Bremen, Germany). During the analysis, the UPLC was separated by a Hypersil GOLD C18 column (100 mm × 2.1 mm, 1.9 µm, Thermo Fisher Scientific), and the mobile phase A was 0.1% formated ultra‐pure water. The mobile phase B was 0.1% formated pure acetonitrile, the flow rate was set at 0.4 mL/min, the column temperature was 40°C, and the total running time was 17 min. Mass spectrometry data were collected in positive and negative ionization modes using an Orbitrap mass spectrometer equipped with a heated electrospray ionization (HESI) source. At the time of analysis, the spray voltage for positive and negative ionization modes was set to 3.5 and 2.5 kV, the capillary temperature was set to 300°C, and the gas flow was set to 50, 13, and 0 AU, respectively. In full scan mode (70–1050 m/z), the resolution is set to 70 000, and the automatic gain control (AGC) target is 3 × 10^6 charges. All sample analyses were carried out in random order to reduce possible sequential effect, and investigators were blinded to group allocation during LC‐MS data acquisition and metabolomics data analysis. LC‐MS data were preprocessed for peak detection, retention‐time alignment, feature extraction, and peak‐area integration using TraceFinder (v5.1) [73, 74, 75]. The identification of metabolites was achieved through comparison of accurate mass and retention time against commercial standard compounds, based on an in‐house library constructed in TraceFinder (v5.1).

#### 4.11.8 Measurement of Steroid Synthesis

After TM3 cells were exposed to different concentrations of MCPP for 24 h, the amount of steroid synthesis was measured. Before treatment, the medium was removed, each well was washed with phosphate‐buffered saline (PBS), then 50 ng/mL human chorionic gonadotropin (hCG) was added to the serum‐free medium for activation, and incubated at 37°C for 2 h. Media were collected to determine steroid content, remaining cells were cleaved with 0.1N sodium hydroxide.

#### 4.11.9 Leydig Cells Isolation

Leydig cells were isolated and extracted from 6‐ to 8‐week‐old adult male C57 mice after one week of adaptive feeding. Briefly, the testes were dissected and decapsulated in RPMI 1640 medium (GIBCO, Grand Island, NY, USA), then dispersed in conical tubes containing 0.5 mg/mL collagenase (type IV, Sigma–Aldrich) and incubated in a shaking incubator at 37°C for 15 min. After briefly holding the tubes in place, the supernatant was collected. The Leydig cells were purified using a discontinuous Percoll density gradient (36% and 60% in PBS), and the gradient was centrifuged at 800 × *g* for 30 min. The interface between the 36% and 60% phases was collected, and the purity of the Leydig cells was found to be greater than 90%. Leydig cells were exposed to 1 µM MCPP, selected based on preliminary cell experiments and population exposure levels [76], or 0.1 µM Orteronel, chosen according to previously reported IC_50_ values for CYP17A1 for 24 h [77], after which the culture supernatant and cells were collected.

#### 4.11.10 Determination of T

T levels in Leydig cells were measured using a mouse T ELISA kit (Jiancheng, Nanjing, China) in accordance with the manufacturer's protocol.

#### 4.11.11 Washout‐Recovery Assay

Primary mouse Leydig cells were seeded and exposed to MCPP (1 µM) or Orteronel (0.1 µM) for 24 h under standard culture conditions. Following exposure, cells were washed five times with sterile PBS to remove residual compounds. Fresh compound‐free medium was then added, and the cultures were maintained for an additional 24 h (washout phase) [78]. At the end of the recovery period, both cell pellets and culture supernatants were collected for subsequent measurements of CYP17A1 enzymatic activity and T levels. Control groups without compound treatment and continuous exposure groups were processed in parallel.

### 4.12 Statistical Analysis

We used linear regression to investigate associations between EDCs and gonadal hormone biosynthesis‐related indicators. For mixed exposure effects and confounding control, PLS‐DA was utilized [79]. Male participants were divided into three groups based on T and E2 levels: low concentration (T deficiency, E2 deficiency), normal, and high concentration (abnormally high T, abnormally high E2). T deficiency was defined as T< 300 ng/dl, while abnormally high T was defined as T >1000 ng/dl, applicable across all male adult stages [80]. Similarly, E2 deficiency was defined as E2< 10 pg/mL, and abnormally high E2 was defined as E2> 50 pg/mL [81, 82]. Logistic regression compared high or low concentration groups with the normal concentration group, using odds ratios (OR) to evaluate risk. Similarly, PLS‐DA was used to explore mixed exposure effects. We integrated results from linear or logistic regression and PLS‐DA analyses to identify significant associations. Covariates include age, race, poverty income ratio (PIR), body mass index (BMI), serum cotinine, seasons of collection, venipuncture times, urinary creatinine, and drinking status (adults only). Participants were classified by BMI (normal: BMI 18.5–25 kg/m^2^; abnormal: BMI< 18.5 kg/m^2^ or >25 kg/m^2^), race (non‐Hispanic white and other), and serum cotinine levels (below or above LLOD). Collection seasons were divided into November first‐April 30th and May first‐October 31st, and venipuncture times into morning, afternoon, or evening sessions. Drinking status was categorized as drinkers or non‐drinkers. To study the overall effects of EDC classes on the gonadal steroidogenic metabolic pathway, we used a binomial test procedure to compare observed frequencies of dichotomous variables against expected frequencies based on a binomial distribution with a default probability parameter of 0.5 (Figure S20) [83]. Associations with *p*< 0.1 in the regression analyses were considered suggestive and were used to define the set of associations evaluated for directional consistency within each chemical class. For this class‐level analysis, a binomial test was used to determine whether the observed direction of associations differed from random expectation, with *p*< 0.05 considered statistically significant. This two‐step procedure used *p*< 0.1 only for exploratory set definition, whereas statistical significance for directional consistency was based on the binomial test (*p*< 0.05) [84]. In animal and cell experiments, an independent sample *t*‐test was used to compare the level of difference between the two groups. Pearson correlation was used to explore the dose‐response relationship between MCPP and T, as well as the metabolome. During data collection and/or analysis, investigators were blinded to the group allocation. The metabolomics data were further analyzed after total‐sum normalization [85]. Statistical analysis of differential metabolites in metabolomics was performed using the OPLS‐DA model in SIMCA‐P (version 14.1, Umetrics). Metabolites with VIP >1 and *p*< 0.05 were considered statistically significant, accounting for multiple comparisons [86]. VIP values summarize the contribution of each variable in multivariate analysis, and VIP >1 was used as the feature‐selection threshold because it indicates an above‐average contribution to model discrimination and has been commonly used for selecting relevant variables [87]. The methods and database implementation of the web portal are detailed in the Supporting Information.

## Author Contributions

**M.C. Conceptualization**: Methodology: **X.Z**., **K.Z**., **X.J**. Investigation: **Y.C**., L.H., **Yingtong Jiang**, G.Z., X.L., A.O.E., L.F. Data curation: Y.C., L.H., Yingtong Jiang, **G.Z**., **L.L**., **A.O.E**., **L.F**. Formal analysis: Y.C., L.H., Yingtong Jiang. Resources: M.C. Validation: M.Z., X.L., L.L., **Yuqi Jiang**, C.S. Visualization: Y.C., L.H., Yingtong Jiang, M.Z. Software: M.Z., Yuqi Jiang, C.S., L.L. Supervision: M.C. Writing – original draft: Y.C., L.H., Yingtong Jiang, X.Z., K.Z., X.J. Writing – review & editing: M.C. Funding acquisition: M.C. Project administration: M.C.

## Funding

This research was supported by the Natural Science Foundation of China (grant numbers: 82273668 and 82574133), Key Natural Science Foundation of the Jiangsu Higher Education Institutions of China (25KJA330002), the “Qinglan Project” for Excellent Young Backbone Teachers in Jiangsu Province, and the Priority Academic Program Development of Jiangsu Higher Education Institutions (PAPD).

## Conflicts of Interest

The authors declare no conflicts of interest.

## Supporting information




**Supporting File 1**: advs76381‐sup‐0001‐SuppMat.docx.

**Supporting File 2**: advs76381‐sup‐0002‐MovieS1.docx.

## Data Availability

All data are available in the main text or the supplementary materials. The EDC–Hormone Explorer is accessible at https://chembio.njmu.edu.cn/edc‐hormone‐explorer.html.
