# Supplementary material for: Mapping Steroidogenic Perturbations Under Endocrine Disruptor Mixtures Across Demographic Subgroups: Structural and Metabolomic Insights
Source: Adv Sci (Weinh). 2026 Jul 9:e76381. Online ahead of print. doi: 10.1002/advs.76381 (PMC13348348; doi:10.1002/advs.76381)
Supplement: Supplementary file 1 — Supporting File 1: advs76381‐sup‐0001‐SuppMat.docx. [file ADVS-9999-e76381-s001.docx]

**Supporting Information for**

Mapping Steroidogenic Perturbations under Endocrine Disruptor Mixtures across Demographic Subgroups: Structural and Metabolomic Insights

**Authors**

Yanling Chen^1,2^, Lei Huang^1,2^, Yingtong Jiang^1,2^, Guohong Zhao^1,2^, Mengyuan Zhu^1,2^, Xiulian Lu^1,2^, Lang Lang^1,2^, Akinpelumi Oluwatobiloba Esther^1,2^, Longhao Fan^1,2^, Yuqi Jiang^1,2^, Chang Sun^1,2^, Xiaoling Zhang^3^, Kun Zhou^1,2,4^, Xiaoming Ji^1,2,5^, Minjian Chen^1,2,5^*.

**Affiliations**

^1^ State Key Laboratory of Reproductive Medicine and Offspring Health, Center for Global Health, School of Public Health, Nanjing Medical University, Nanjing 211166, China;

^2^ Key Laboratory of Modern Toxicology of Ministry of Education, School of Public Health, Nanjing Medical University, Nanjing 211166, China;

^3^ Department of Hygienic Analysis and Detection, School of Public Health, Nanjing Medical University, Nanjing 211166, China;

^4^ Department of Epidemiology, Center for Global Health, School of Public Health, Nanjing Medical University, Nanjing 211166, China;

^5^ Department of Occupational Medicine and Environmental Health, School of Public Health, Key Laboratory of Public Health Safety and Emergency Prevention and Control Technology of Higher Education Institutions in Jiangsu Province, Nanjing Medical University, Nanjing 211166, China.

**Correspondence**: Minjian Chen ([minjianchen@njmu.edu.cn](mailto:minjianchen@njmu.edu.cn))

**Supplementary Methods**

**Measurements of serum and urine samples for PFASs**

Samples were shipped frozen on dry ice to the National Center for Environmental Health for testing. Serum PFASs were measured using online solid phase extraction-high performance liquid chromatography-turbo ion spray-tandem mass spectrometry (online SPE-HPLC-TIS-MS/MS) while urinary PFASs were measured using online SPE-HPLC-TIS-MS/MS. Details of the analytical methodology can be found in the laboratory procedure manual on the National Health and Nutrition Examination Survey (NHANES) website. We included 11 PFASs in our study: perfluorodecanoic acid (PFDA), perfluorohexane sulfonic acid (PFHxS), 2-(N-methyl-perfluorooctane sulfonamido) acetic acid (MPAH), perfluoroheptanoic acid (PFHpA), perfluorononanoic acid (PFNA), perfluoroundecanoic acid (PFUA), perfluorobutanoic acid (PFBA), perfluorohexanoic acid (PFHxA), n-perfluorooctane sulfonic acid (n-PFOS), perfluoromethylheptane sulfonic acid isomers (Sm-PFOS), and n-perfluorooctanoic acid (PFOA). PFBA and PFHxA were detected in urine. The lower limits of detection (LLODs) were 0.10 ng/mL for PFASs measured in NHANES 2013-2014 and 2015-2016.

**Measurements of urine samples for PAEs**

Urine samples were shipped frozen on dry ice to the National Center for Environmental Health for testing. A minimum sample volume of 0.5 mL is preferred. Samples may be stored frozen at temperatures at or below -40°C for several years prior to analysis. PAEs were measured using high performance liquid chromatography-electrospray ionization-tandem mass spectrometry (HPLC/ESI-MS/MS). Details of the analytical methodology can be found in the laboratory procedure manual on the NHANES website. The LLODs were 0.2 ng/mL, 0.3 ng/mL, 0.4 ng/mL, 0.4 ng/mL, 0.4 ng/mL, 0.4 ng/mL, 0.5 ng/mL, 1.2 ng/mL, 0.4 ng/mL, 0.4 ng/mL, 0.4 ng/mL, 0.8 ng/mL, 0.8 ng/mL, 0.9 ng/mL, 0.2 ng/mL and 0.3 ng/mL for MCNP, MCOP, MECPP, MHiBP, MBP, MCPP, MCOCH, MEP, MHBP, MEHHP, MHINCH, MEHP, MiBP, mono-isononyl phthalate (MiNP), MEOHP and MBzP, measured in NHANES 2013-2014 and 2015-2016, respectively.

**Measurements of urine samples for phenols and parabens**

Urine samples were processed, stored, and shipped to the Division of Laboratory Sciences, National Center for Environmental Health, CDC, Atlanta, GA, for analysis. Samples were stored under appropriate frozen conditions (-20°C) until shipped to the National Center for Environmental Health for testing. Phenols and parabens were measured using online SPE-HPLC-isotope dilution-MS/MS (online SPE-HPLC-ID-MS/MS). Details of the analytical methodology can be found in the laboratory procedure manual on the NHANES website. The LLODs were 0.4 ng/mL, 0.2 ng/mL, 0.2 ng/mL, 0.1 ng/mL, 0.1 ng/mL, 1.7 ng/mL, 0.1 ng/mL, 1.0 ng/mL, 1.0 ng/mL, 0.1 ng/mL for BP-3, BPA, bisphenol F (BPF), BPS, TCC, Triclosan (TCS), butyl paraben (BP), ethyl paraben (EP), methyl paraben (MP), and PP, measured in NHANES 2013-2014 and 2015-2016, respectively.

**Measurements of serum samples for gonadal hormones and serum SHBG**

Serum samples were stored under -20°C before shipped to the National Center for Environmental Health for testing. Serum testosterone (T) and estradiol (E2) were measured using isotope dilution high performance liquid chromatography tandem mass spectrometry (ID-LC-MS/MS), while sex hormone-binding globulin (SHBG) was measured using a sex hormone-binding globulin immunoassay. Details of the analytical methodology can be found in the laboratory procedure manual on the NHANES website. The LLODs were 0.75 ng/ml, 2.994 pg/mL and 0.800 nmol/L for T, E2 and SHBG, respectively.

**Measurements of serum samples for HDL-C, LDL-C, TG, and TC**

Serum samples were stored under appropriate frozen conditions (-30°C) until shipped to the University of Minnesota for testing. HDL-C, TC and TG were measured on the Roche modular P and Roche Cobas 6,000 chemistry analyzers. LDL-C was calculated using the Friedewald calculation:

$[LDL-C] = [TC] - [HDL-C] - [TG/5]$ (1)

*where all values were expressed in mg/dL. This calculation was valid for TG values less than or equal to 400 mg/dL.*

Details of the analytical methodology can be found in the laboratory procedure manual on the NHANES website. The LLODs were 3 mg/dL, 4 mg/dL and 9 mg/dL for HDL-C, TC and TG, respectively. Because data on LDL-C as well as TG are available only in people older than 12 years, we analyzed the association of EDCs with lipids only in adult men and women for the sake of standardization.

**Identification of potential MIEs**

To address key findings in adult males, genes related to exogenous chemicals were searched in the Comparative Toxicogenomics Database (CTD, http://ctdbase.org/) using keywords such as “mono-(2-ethylhexyl) phthalate”, “mono-(3-carboxypropyl) phthalate”, “mono-isobutyl phthalate”, and “monobutyl phthalate”. These genes formed the exogenous chemicals-related gene set (ECR-set). Simultaneously, a decreased T-related gene set (DTR-set) was created from GeneCards (https://www.genecards.org/) using the keyword “decreased testosterone”, with a GeneCards Inferred Functionality Score (GIFtS) and relevance score (both≥20) to select pertinent genes [1]. Overlapping genes in the ECR-set and DTR-set were analyzed using the Genotype-Tissue Expression (GTEx) Portal (https://gtexportal.org/home/), focusing on genes expressed in testicular tissue compared to other tissues (i.e., among the top 10 expressed genes in the testis [1]). Potential MIEs were identified by manually screening genes expressed in testis that are involved in hormone synthesis or related to the hypothalamic-pituitary-gonadal axis.

**Molecular docking**

AutoDock Vina 1.1.2 (http://vina.scripps.edu/) was used for molecular docking. Ligand structures were downloaded from PubChem (https://pubchem.ncbi.nlm.nih.gov/) and energy-minimized before docking. Protein structures were obtained from the Protein Data Bank for CYP11A1, CYP17A1, and StAR (PDB IDs: 3N9Y, 3RUK and 3P0L, respectively), and from the AlphaFold Protein Structure Database for NR0B1 and NR5A1 (model IDs: AF-P51843-F1-model_v4 and AF-Q13285-F1-model_v4, respectively). Before docking, receptor structures were prepared by removing water molecules and non-essential ligands, adding hydrogen atoms, assigning charges, and converting structures into the AutoDock Vina-compatible format. Binding energy (kcal/mol) value indicating the binding capacity, where lower values signify more stable ligand-receptor binding. Visual analysis was conducted using PyMOL (<https://pymol.org/2/>).

**Assessment of the AOP framework**

PubMed was utilized to gather evidence supporting the AOP framework. Aspects of AOP assessment included (1) the biological domain of AOP applicability, (2) the essentiality of KEs, and (3) the evidence supporting KERs. Confidence levels were classified as “high”, “moderate”, or “low” following guidelines from the Organization for Economic Co-operation and Development (OECD) handbook of AOPs.

**Statistical analysis**

Due to the influence of menopause on hormone levels, we divided female adults into pre- and postmenopausal groups for further correlation tests [2]. For sensitivity analysis, considering that sampling weights can reduce the accuracy of estimates and lead to excessive adjustment bias, we used unweighted data for our analysis similar to previous studies [3, 4]. However, to assess the impact of survey weights on outcomes, survey weights were added to the models. In binomial test, we assumed *β* and OR parameter directions followed a random distribution with equal probabilities. Linear and logistic regression results with *P*<0.1 were categorized into four sets based on EDCs’ types [5]. We computed the proportion of positive and negative directions within these sets and conducted a statistical test on the hypothesized proportion, with significance set at *P*<0.05 (**Figure S20**).

**Database implementation of the web portal**

To maximize accessibility and reproducibility, the portal was implemented as a static, client-side application using HTML5, CSS3, and JavaScript (ES6+), such that all core functions run in a modern web browser without requiring dedicated backend services for routine use. Interactive network rendering was implemented with D3.js (v7).

Association results were preprocessed using Python (Pandas) to convert tabular outputs into structured JSON assets consumed by the browser. To support robust chemical lookup across naming conventions and identifiers, we implemented a multi-tier search strategy that combines exact matching, substring matching, identifier matching, and fuzzy matching based on Levenshtein distance; candidate matches are ranked using a heuristic scoring scheme that prioritizes exact and identifier-level matches while retaining sensitivity to common synonyms and spelling variants. Retrieved chemicals are linked to standardized identifiers and cross-references curated across commonly used chemical resources (e.g., CAS Registry, PubChem, ChEBI, ChEMBL, CompTox Dashboard, InChIKey, PubChem Substance, MeSH) and supplemented with equivalent terms/synonyms to improve retrieval.

The portal was deployed using standard HTTP static hosting and is accessible through modern web browsers without specialized software installation, supporting FAIR-aligned dissemination of the processed association resource. An overview of the system architecture and core functions is provided in **Figure S17–S18**. The current release will be maintained by the study team as a stable version of the association results, and the website will be reviewed at least annually to ensure accessibility, repair technical issues, and update documentation when needed. For long-term data sharing, the processed association results, chemical annotations, and downloadable tables will remain accessible through the EDC-Hormone Explorer website, while the underlying data remain available from the public repository.

**Supplementary Results**

**Interactive web tool enables in-depth exploration of chemical–hormone landscape**

We developed an interactive web-based platform (Chemical–Hormone Association Explorer) that allows users to query chemicals, apply demographic stratification, and visualize chemical–hormone (and related biomarker) association results generated in this study. The interface integrates three core functions. First, users can search by chemical name/abbreviation and refine outputs through stratified filtering (e.g., study population subgroup and user-defined statistical/importance thresholds). Second, a chemical annotation panel displays standardized identifiers and classification fields, with missing entries automatically hidden to preserve readability. Third, a detailed association table summarizes key metrics (e.g., association direction and effect estimates) and supports CSV export (UTF-8) for downstream analyses. For visual synthesis, the Explorer provides an interactive association network rendered with a force-directed layout, in which edges encode association direction and relative effect magnitude; users can iteratively add additional exogenous chemicals to expand the network and compare multi-chemical patterns under identical population and filtering settings (**Figure S17–S18**).

**Supplementary Figures**

**
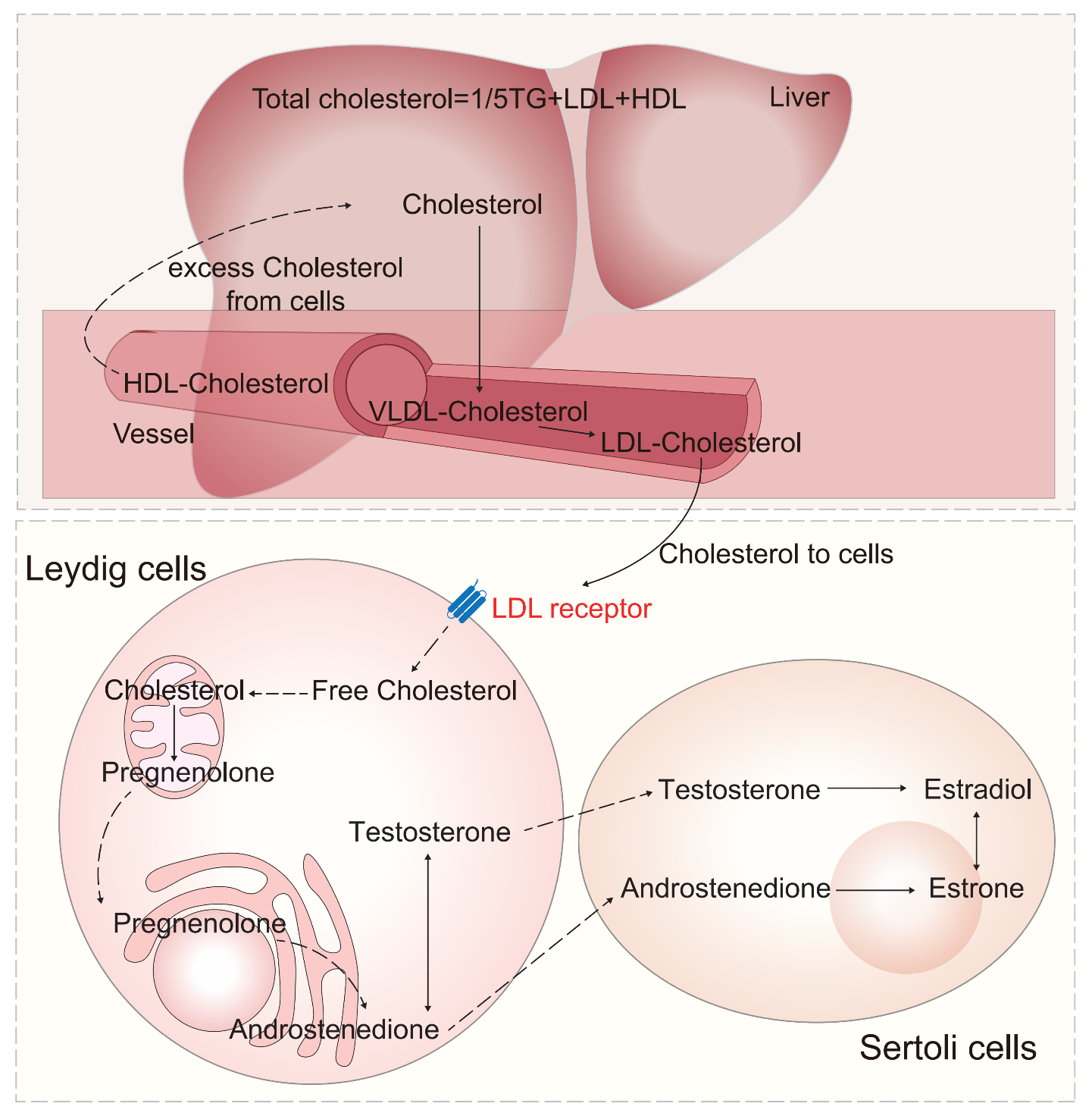
**

Figure S1. The metabolic relationship between molecules in the gonadal steroidogenic metabolic pathway. HDL-C is responsible for transporting excess cholesterol back to the liver for metabolism. The endogenous lipoprotein pathway begins in the liver, generating VLDL-C. The triglycerides in VLDL-C are metabolized by lipoprotein lipase, releasing free fatty acids and further metabolized to LDL-C, which delivers cholesterol to Leydig cells. In these cells, free cholesterol is internalized via LDLR-mediated endocytosis, converted to pregnenolone, and subsequently transformed into T, ultimately leading to the synthesis of E2. Thus, the metabolism of cholesterol is closely related to the synthesis of sex hormone.


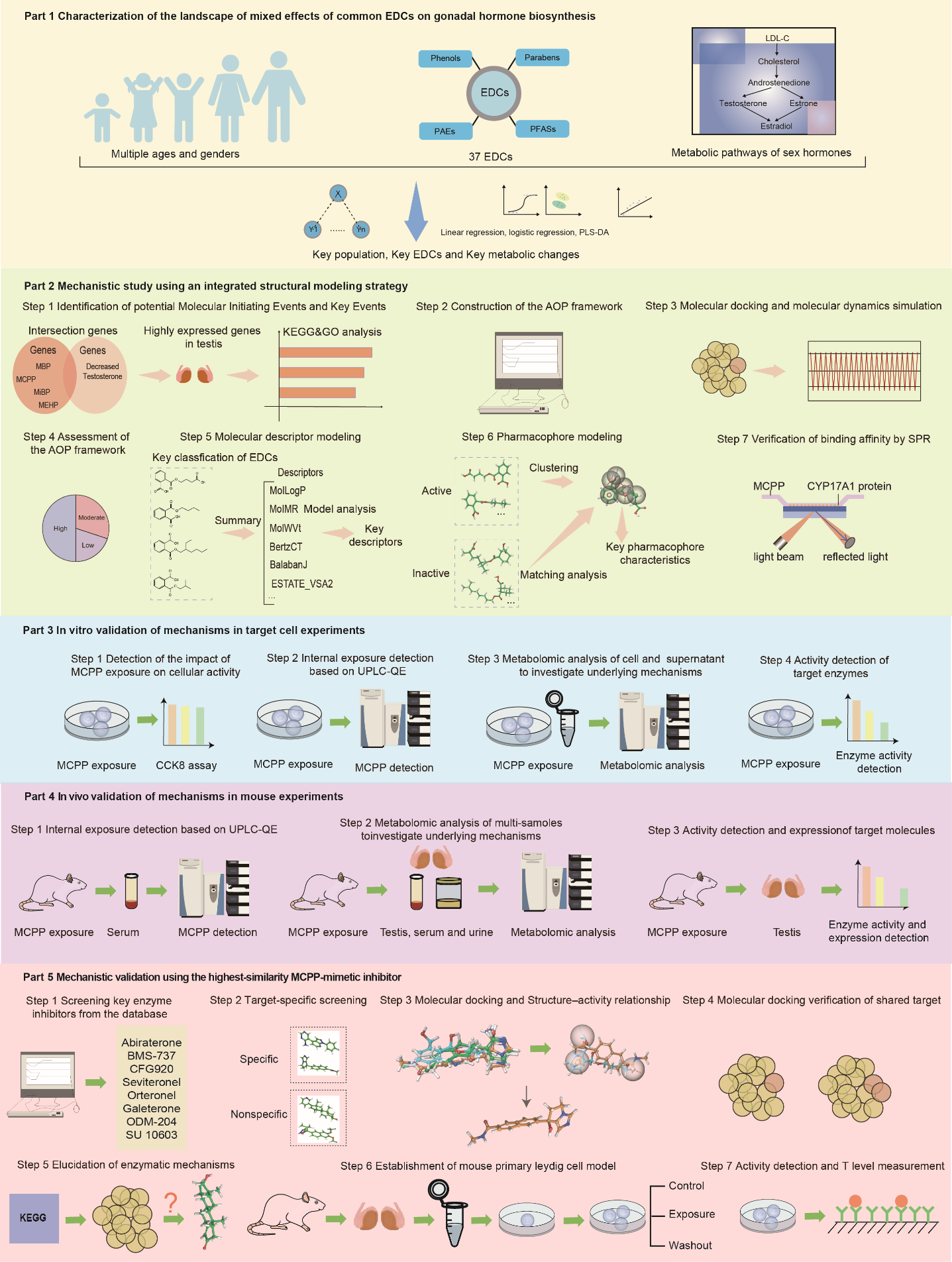


Figure S2. The overall experimental design.


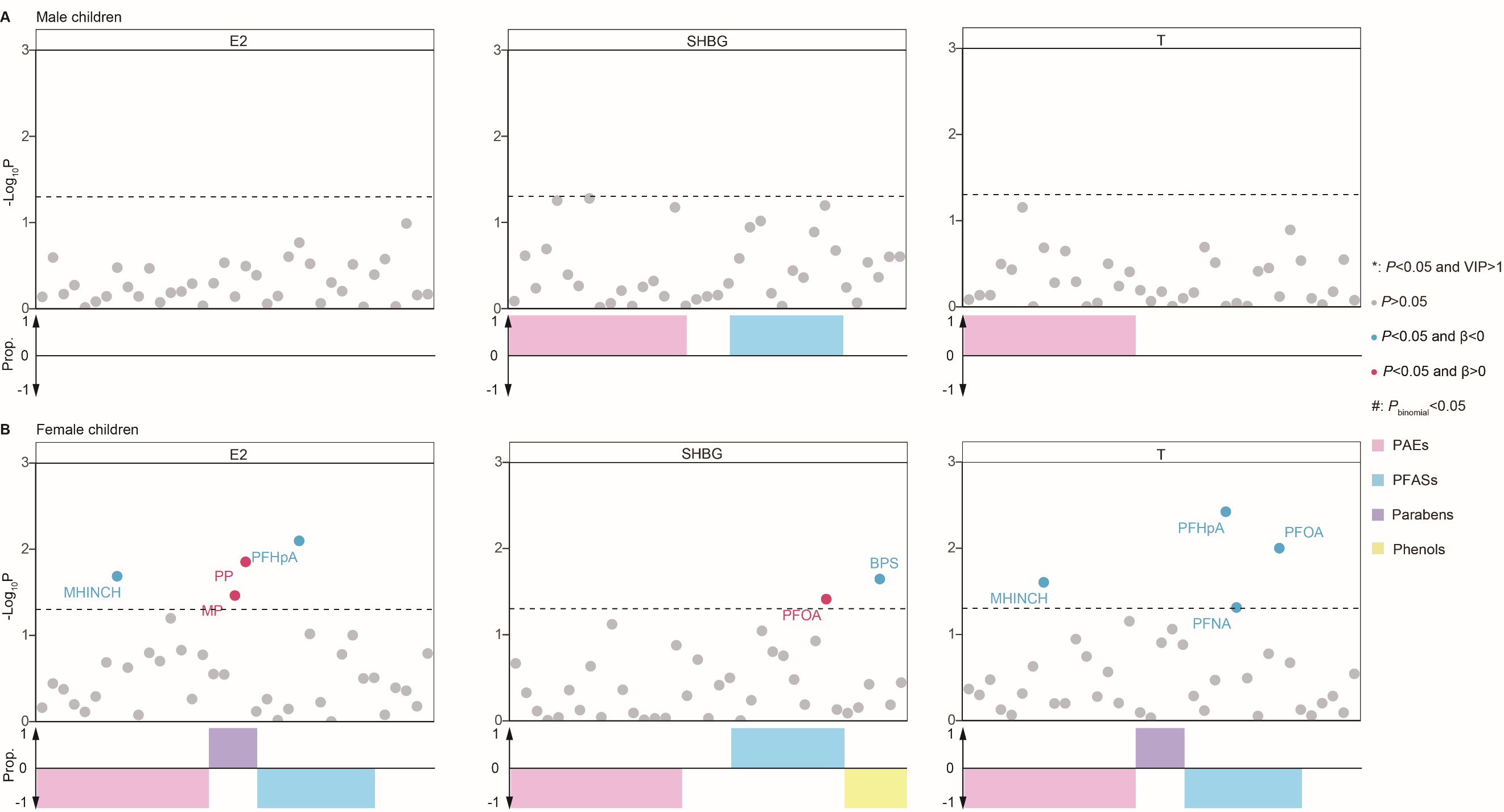


Figure S3. Comprehensive analysis of EDCs and molecules in the gonadal steroidogenic metabolic pathway in children: linear regression, PLS-DA, and correlation percentages. (A) Linear regression and PLS-DA analysis of EDCs and molecules in the gonadal steroidogenic metabolic pathway in male children. The axis for prop. represents percentages coming from the binomial test described in the statistical analysis, with values above zero indicating positive correlations and values below zero indicating negative correlations. "#" means the statistical test is significant. (B) Linear regression and PLS-DA analysis of EDCs and molecules in the gonadal steroidogenic metabolic pathway in female children. The axis for prop. represents percentages coming from the binomial test described in the statistical analysis, with values above zero indicating positive correlations and values below zero indicating negative correlations. "#" means the statistical test is significant.

**
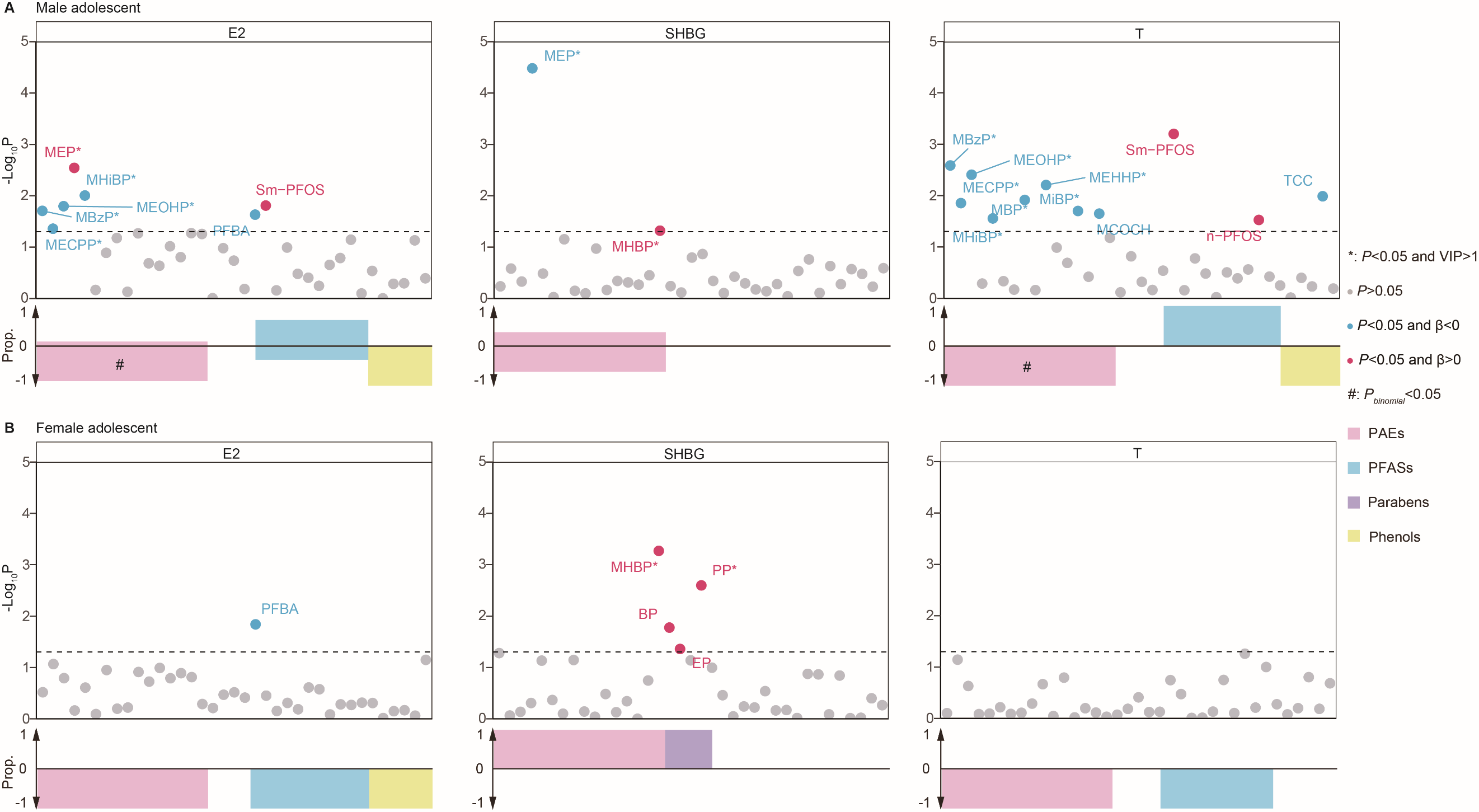
**

Figure S4. Comprehensive analysis of EDCs and molecules in the gonadal steroidogenic metabolic pathway in adolescents: linear regression, PLS-DA, and correlation percentages. (A) Linear regression and PLS-DA analysis of EDCs and molecules in the gonadal steroidogenic metabolic pathway in male adolescents. The axis for prop. represents percentages coming from the binomial test described in the statistical analysis, with values above zero indicating positive correlations and values below zero indicating negative correlations. "#" means the statistical test is significant. (B) Linear regression and PLS-DA analysis of EDCs and molecules in the gonadal steroidogenic metabolic pathway in female adolescents. The axis for prop. represents percentages coming from the binomial test described in the statistical analysis, with values above zero indicating positive correlations and values below zero indicating negative correlations. "#" means the statistical test is significant.

**
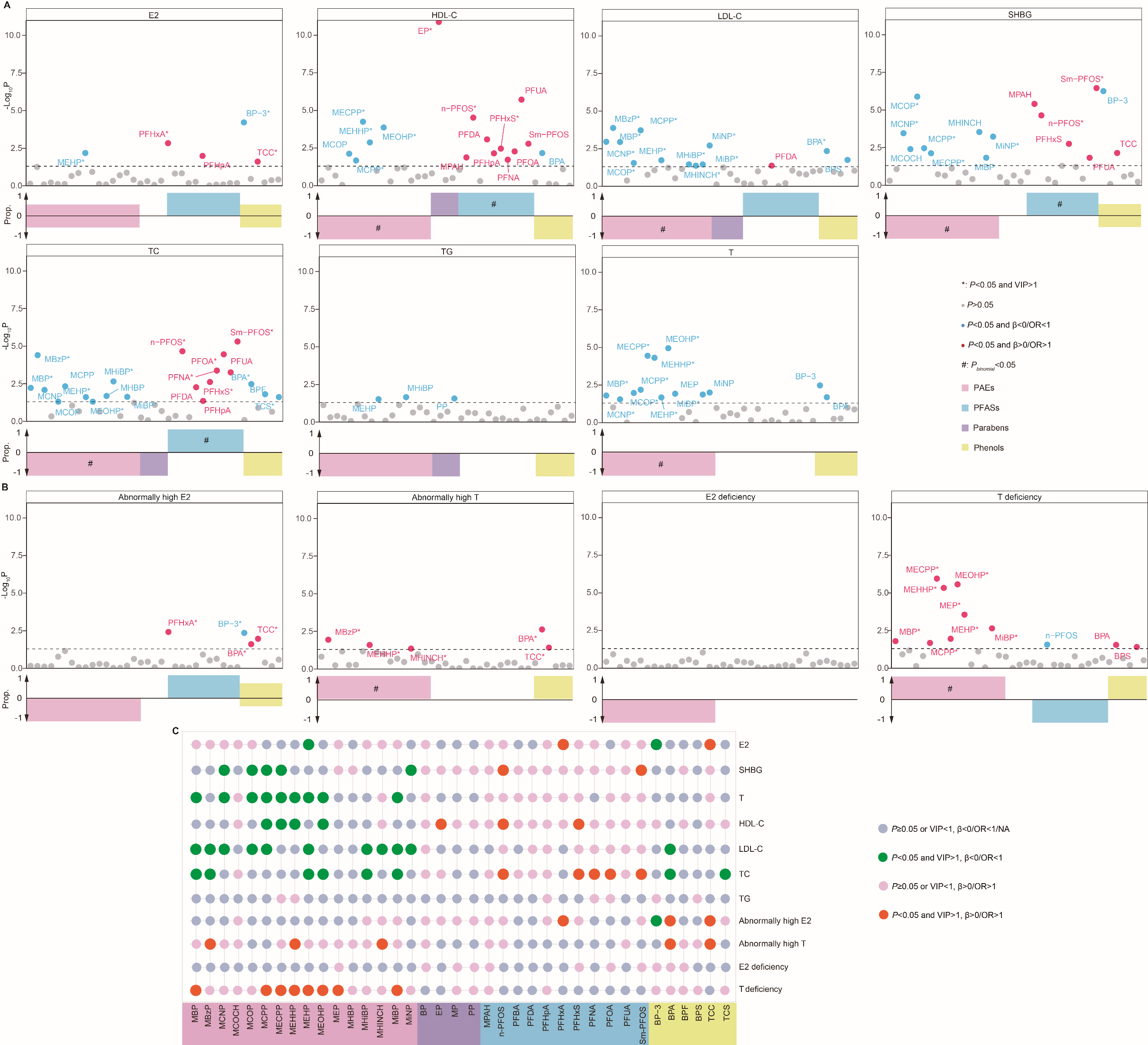
**

Figure S5. Comprehensive analysis of EDCs and molecules in the gonadal steroidogenic metabolic pathway in male adults: linear and logistic regression, PLS-DA, and correlation percentages. (A) Linear regression and PLS-DA analysis of EDCs and molecules in the gonadal steroidogenic metabolic pathway. The axis for prop. represents percentages coming from the binomial test described in the statistical analysis, with values above zero indicating positive correlations and values below zero indicating negative correlations. "#" means the statistical test is significant. (B) Logistic regression and PLS-DA analysis of EDCs and gonadal hormones abnormalities. The axis for prop. represents percentages coming from the binomial test described in the statistical analysis, with values above zero indicating positive correlations and values below zero indicating negative correlations. "#" means the statistical test is significant. (C) Summary of the associations between EDCs and molecules in the gonadal steroidogenic metabolic pathway and gonadal hormones abnormalities.

**
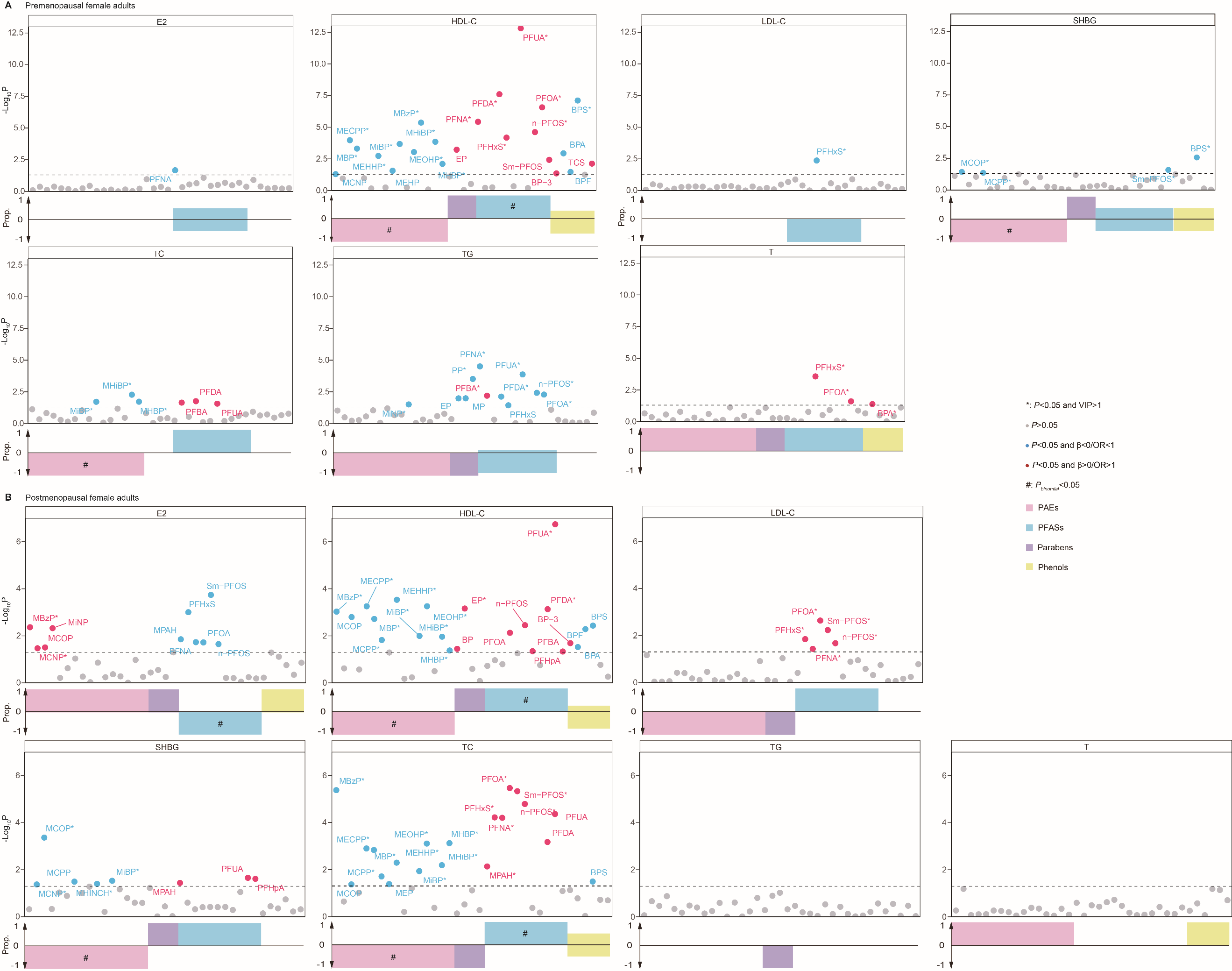
**

Figure S6. Comprehensive analysis of EDCs and molecules in the gonadal steroidogenic metabolic pathway in female adults: linear regression, PLS-DA, and correlation percentages. (A). Linear regression and PLS-DA analysis of EDCs and molecules in the gonadal steroidogenic metabolic pathway in premenopausal female adults. The axis for prop. represents percentages coming from the binomial test described in the statistical analysis, with values above zero indicating positive correlations and values below zero indicating negative correlations. "#" means the statistical test is significant. (B) Linear regression and PLS-DA analysis of EDCs and molecules in the gonadal steroidogenic metabolic pathway in postmenopausal female adults. The axis for prop. represents percentages coming from the binomial test described in the statistical analysis, with values above zero indicating positive correlations and values below zero indicating negative correlations. "#" means the statistical test is significant.


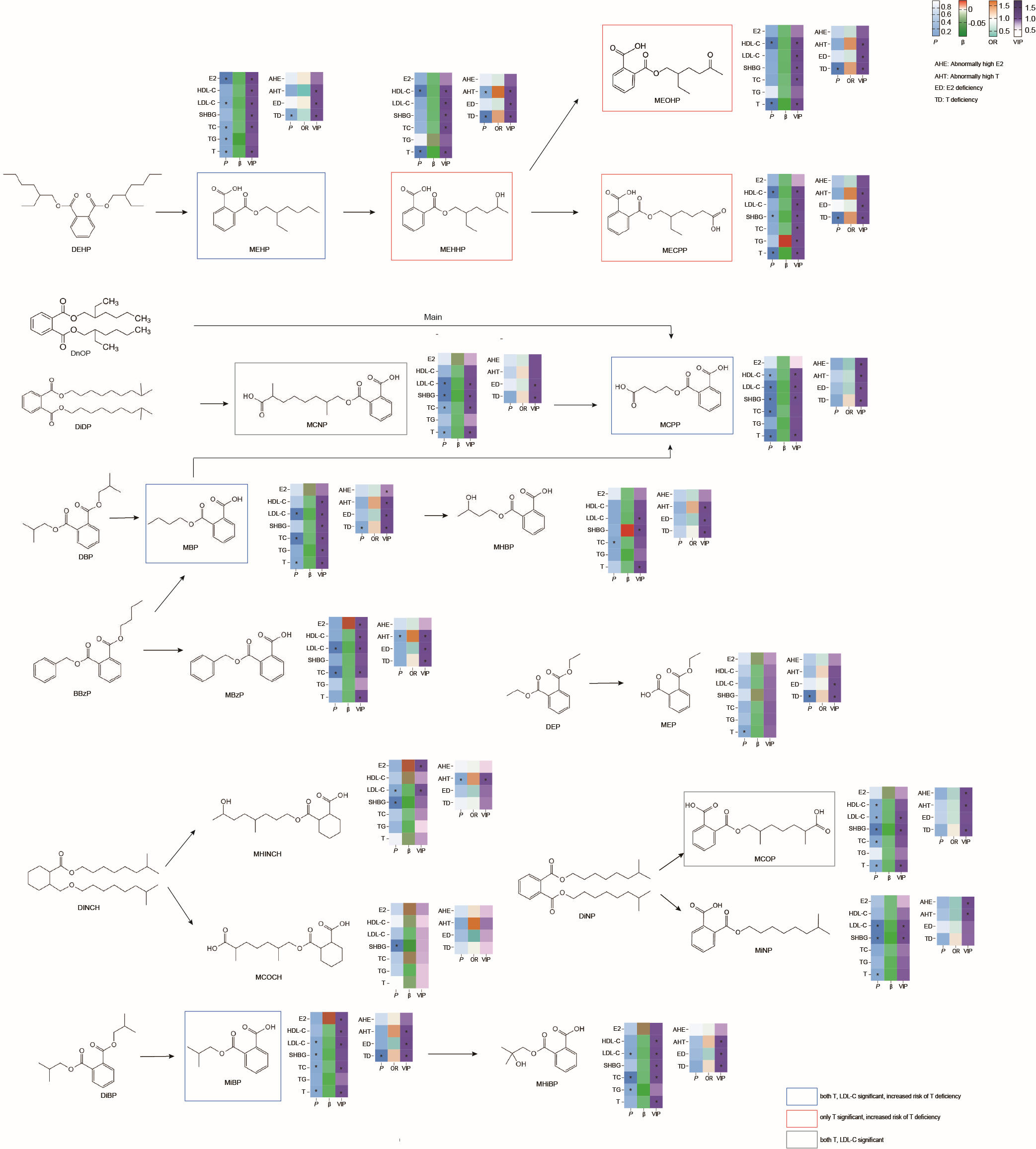


Figure S7. Summary of metabolic processes and statistical results for PAEs in the human body. Blue boxes represent chemicals that significantly affect both T and LDL-C, increasing the risk of T deficiency. Red boxes represent chemicals that significantly affect only T, increasing the risk of T deficiency. Gray boxes represent chemicals that significantly affect both T and LDL-C. Adjacent heatmaps summarize the results of linear regression, logistic regression, and PLS-DA models for each chemical.


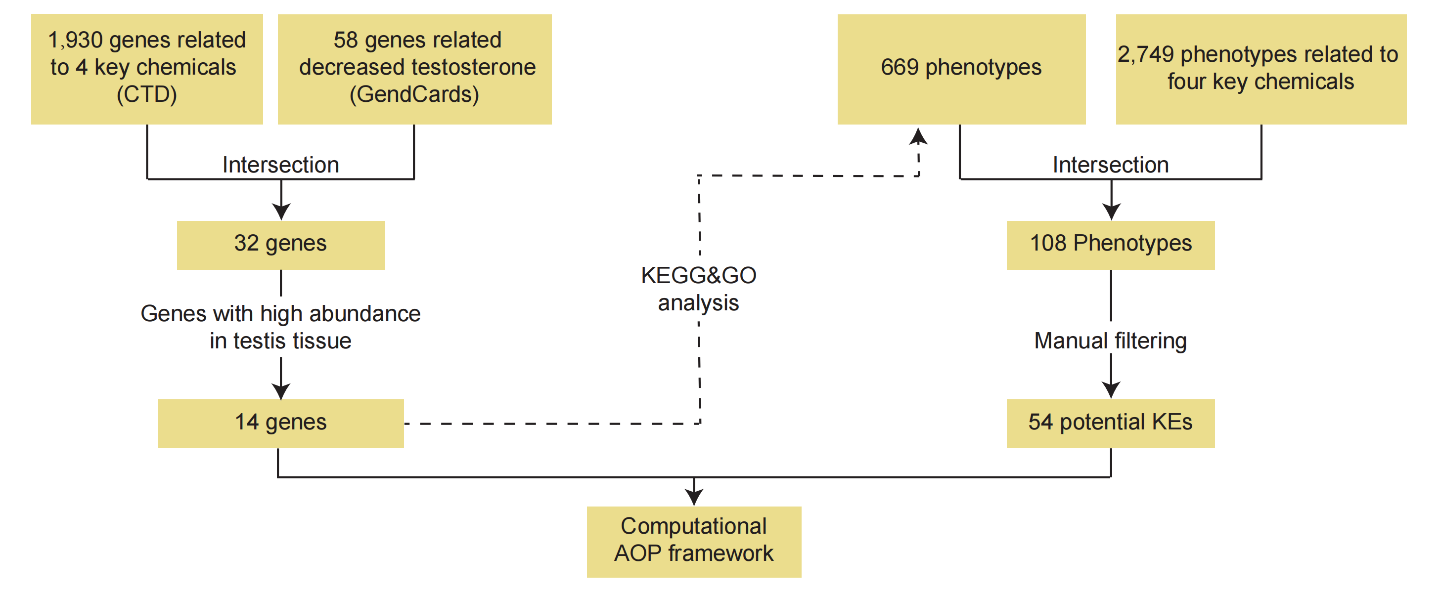


Figure S8. Flow diagram of computational AOP framework construction.


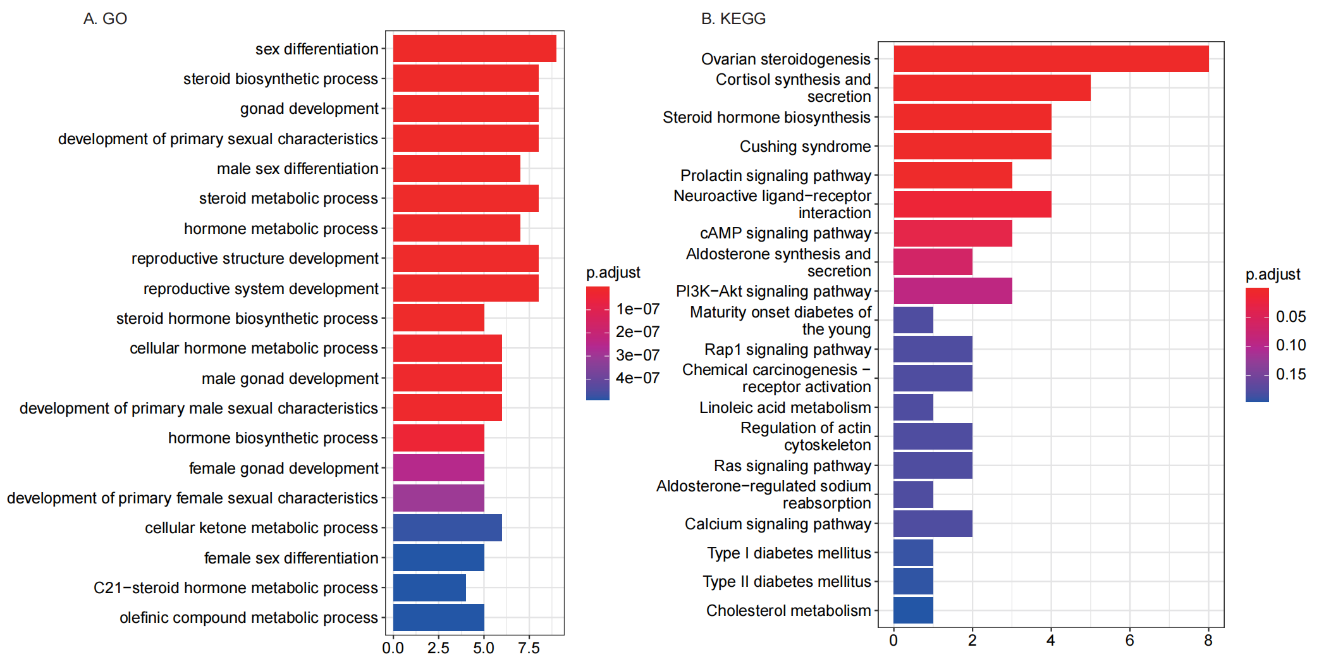


Figure S9. Top 20 phenotypes related to key chemicals. (A) GO terms. (B) KEGG pathways.


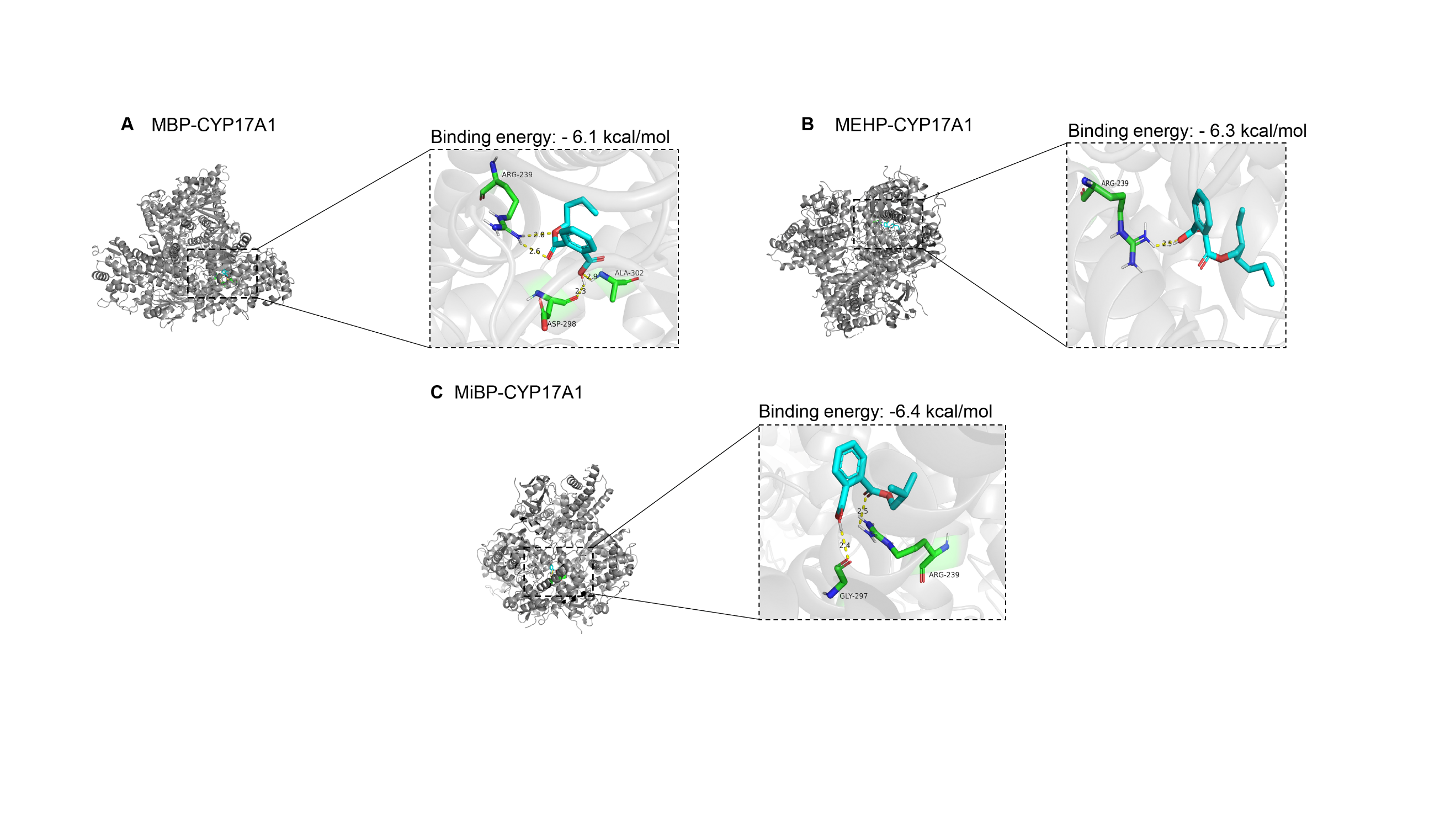


Figure S10. Molecular docking analysis of CYP17A1 with A) MBP, B) MEHP, and C) MiBP. MBP, mono-n-butyl phthalate; MCPP, mono-(3-carboxypropyl) phthalate; MEHP, mono-(2-ethyl)-hexyl phthalate; MiBP, mono-isobutyl phthalate; CYP17A1, Cytochrome P450 Family 17 Subfamily A Member 1.


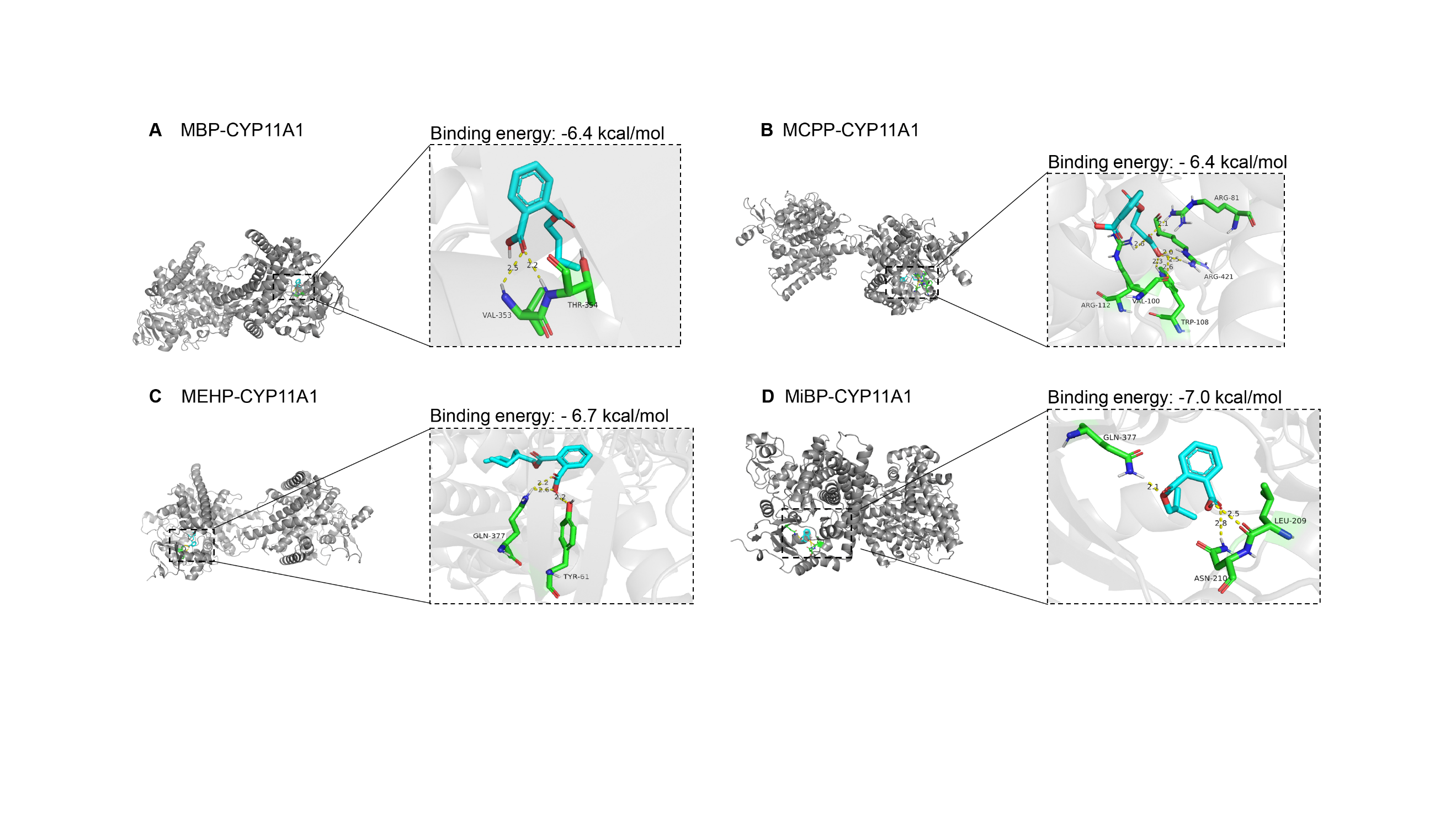


Figure S11. Molecular docking analysis of CYP11A1 with A) MBP, B) MCPP, C) MEHP and D) MiBP. MBP, mono-n-butyl phthalate; MCPP, mono-(3-carboxypropyl) phthalate; MEHP, mono-(2-ethyl)-hexyl phthalate; MiBP, mono-isobutyl phthalate; CYP11A1, Cytochrome P450 Family 11 Subfamily A Member 1.


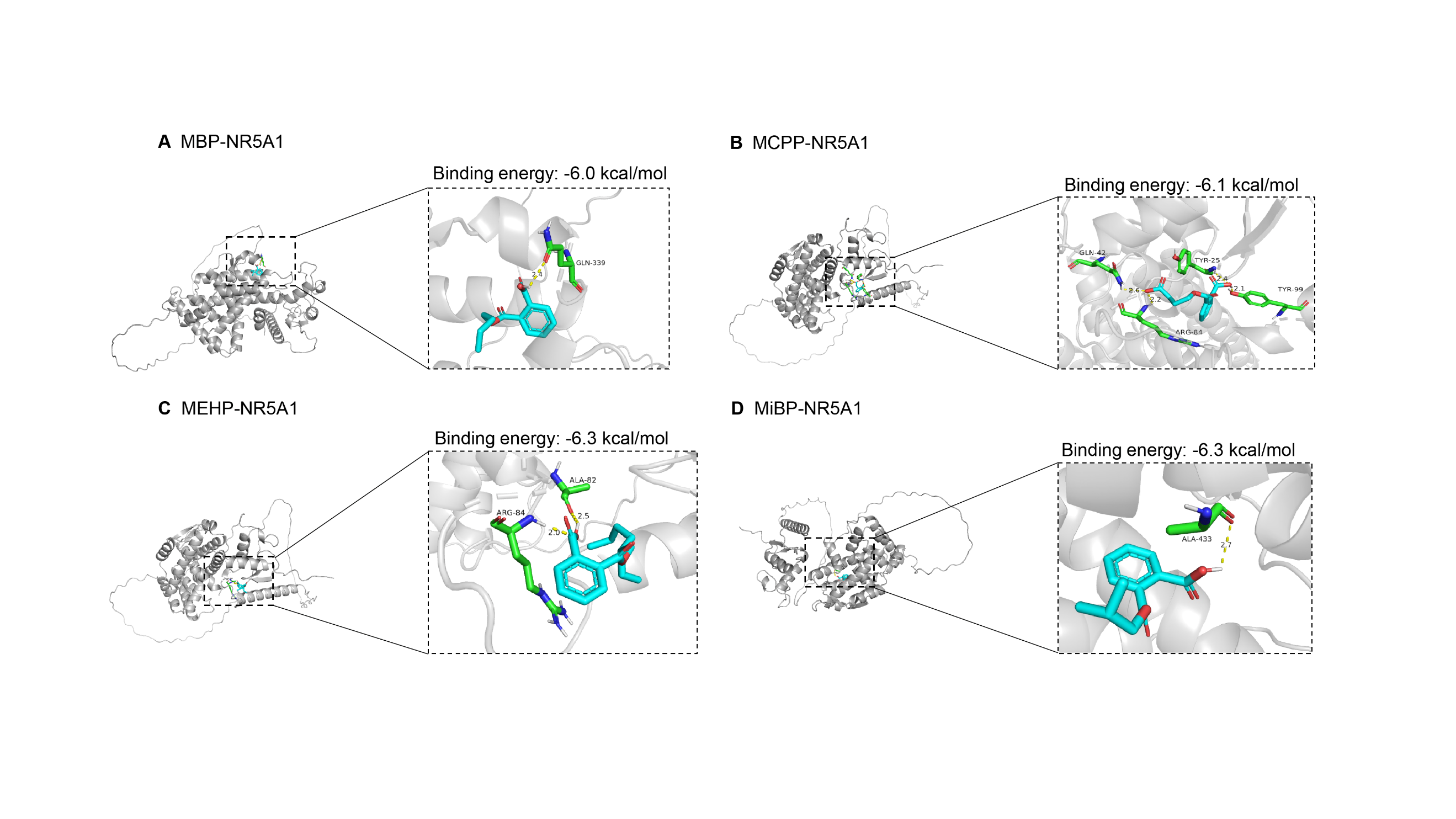


Figure S12. Molecular docking analysis of NR5A1 with A) MBP, B) MCPP, C) MEHP and D) MiBP. MBP, mono-n-butyl phthalate; MCPP, mono-(3-carboxypropyl) phthalate; MEHP, mono-(2-ethyl)-hexyl phthalate; MiBP, mono-isobutyl phthalate; NR5A1, Nuclear Receptor Subfamily 5 Group A Member 1.


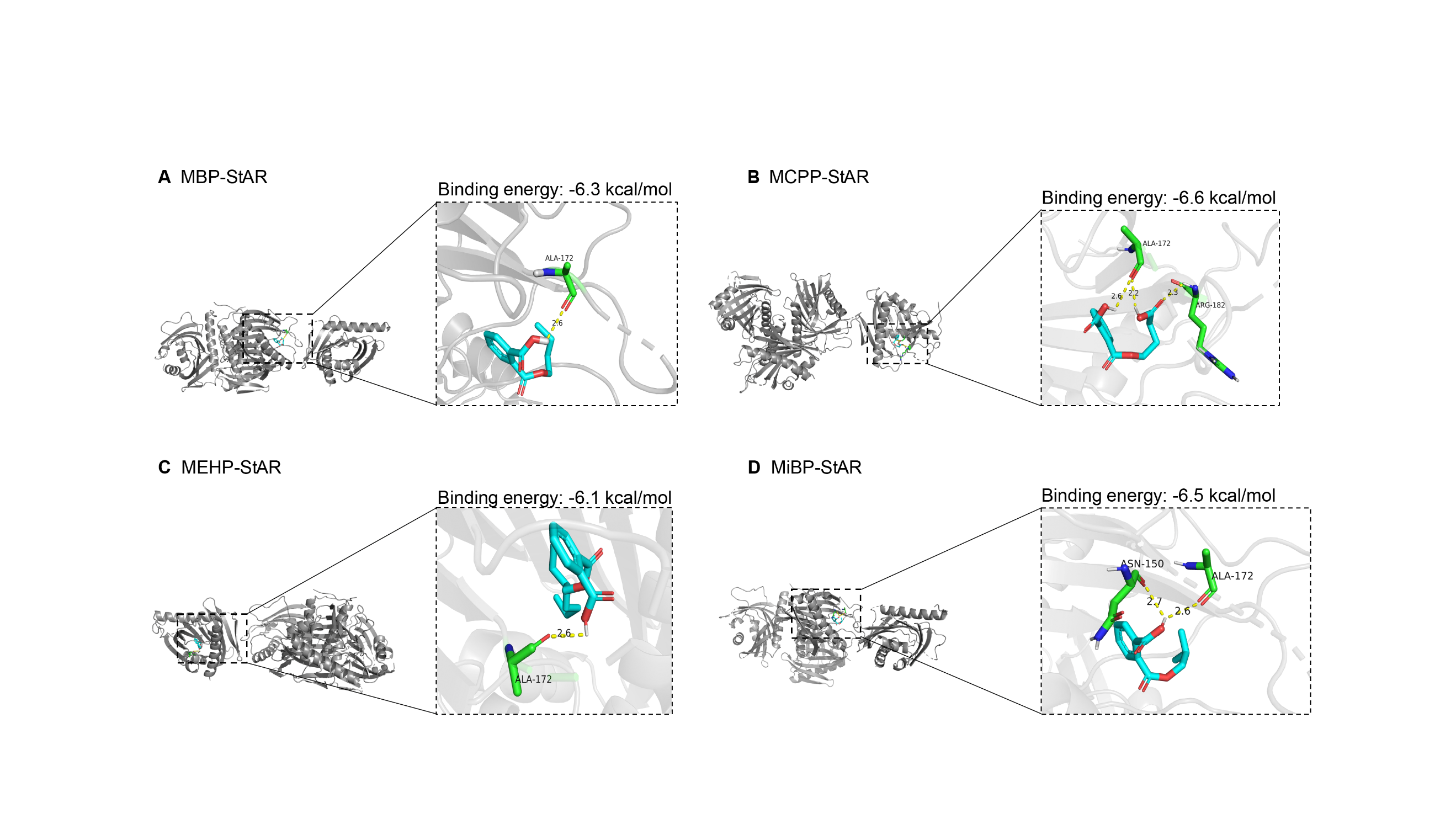


Figure S13. Molecular docking analysis of StAR with A) MBP, B) MCPP, C) MEHP and D) MiBP. MBP, mono-n-butyl phthalate; MCPP, mono-(3-carboxypropyl) phthalate; MEHP, mono-(2-ethyl)-hexyl phthalate; MiBP, mono-isobutyl phthalate; StAR, Steroidogenic Acute Regulatory Protein.


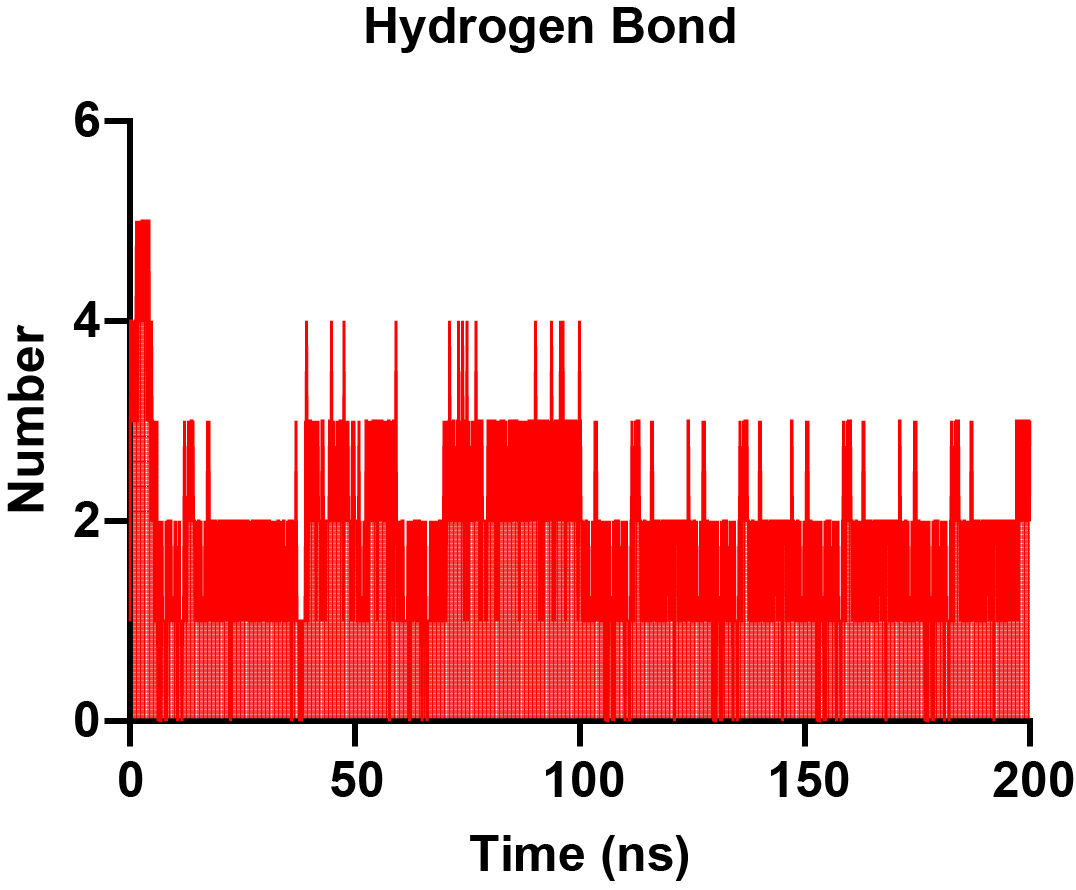


Figure S14. Line chart of the number of hydrogen bonds formed between CYP17A1 and MCPP over time.


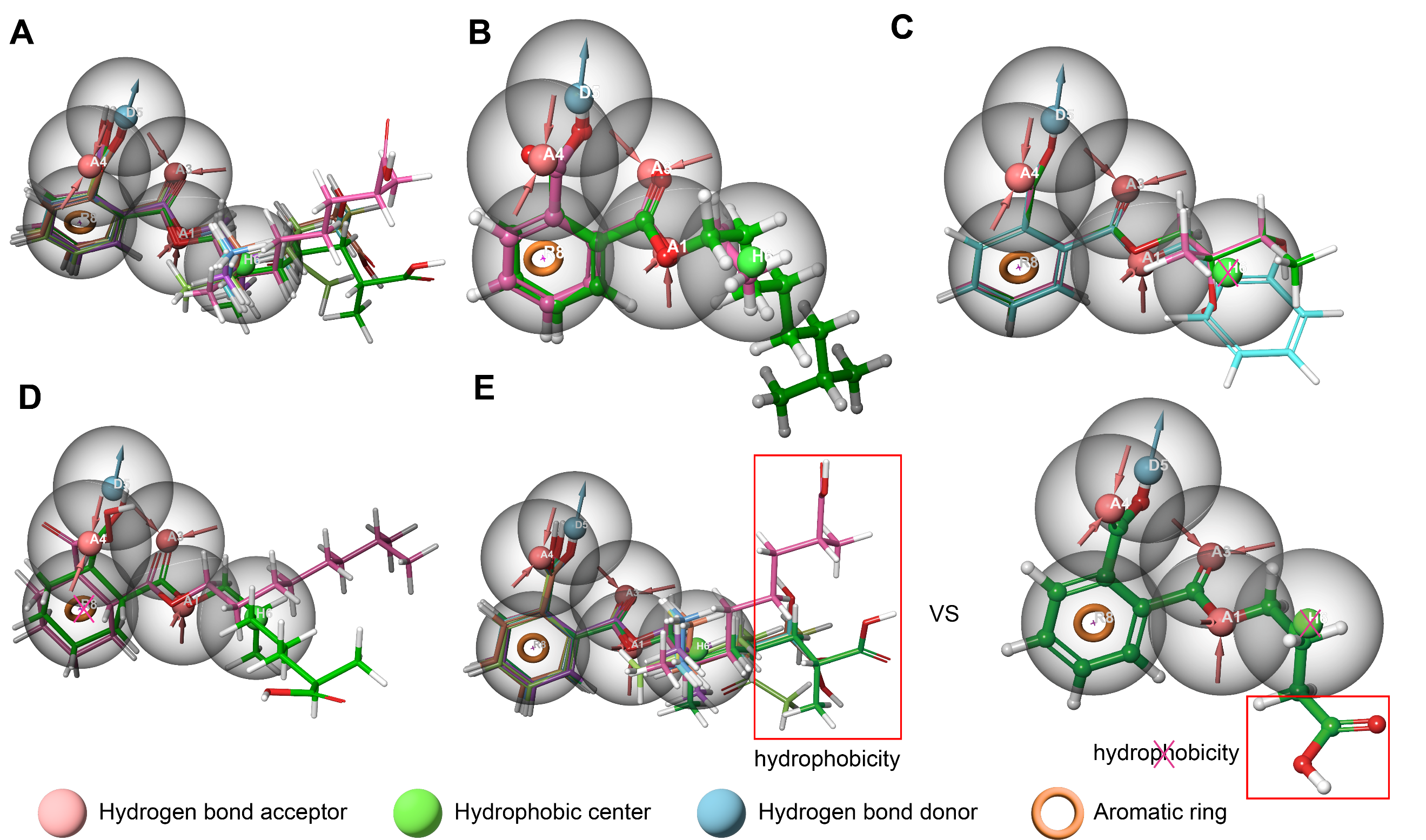


Figure S15. Pharmacophore features among active and inactive molecules: AAADHR analysis. (A) Eight active molecules possess the AAADHR pharmacophore features (*P*<0.05, VIP>1). (B) Two inactive molecules possess the AAADHR pharmacophore features but with suggestive effect (*P*<0.05, VIP<1). (C) Three inactive molecules partially possess the AAADHR pharmacophore features, namely AAADR (*P*>0.09). (D) Two inactive molecules partially possess the AAADHR pharmacophore features, namely AAADH (*P*>0.90). (E) MCPP has one less hydrophobic center compared to other active PAEs.
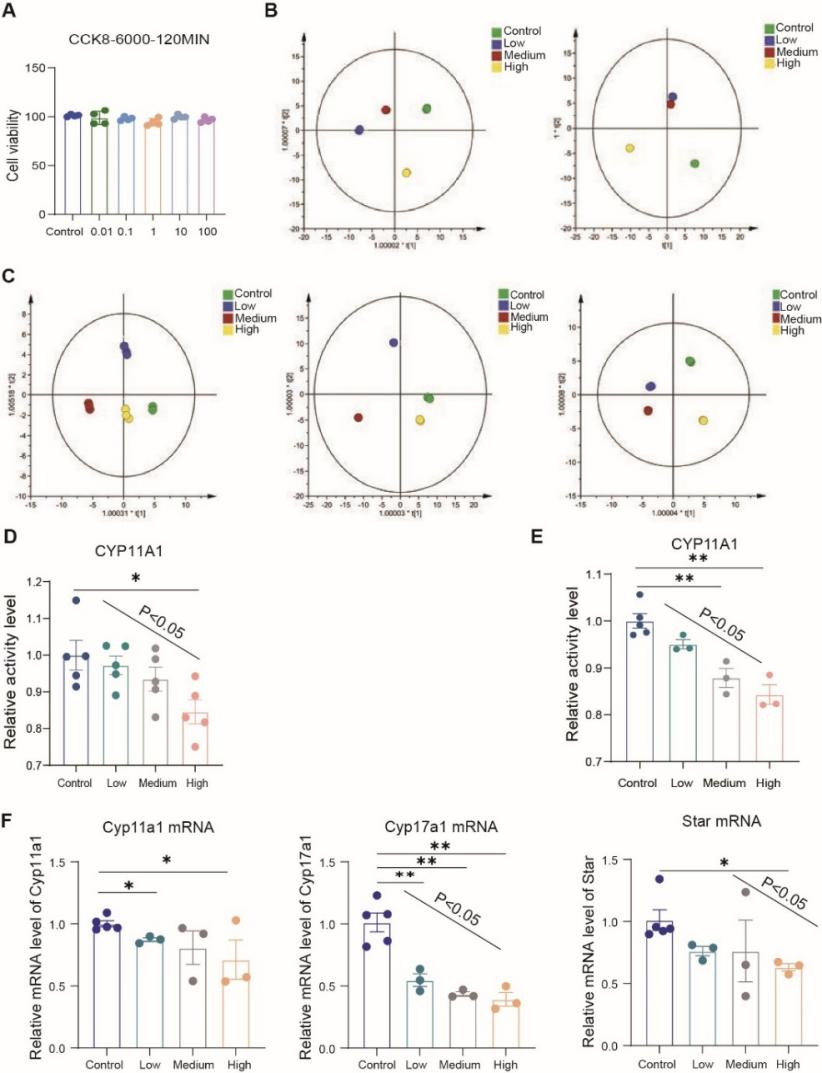


Figure S16. Results of in vitro and in vivo experiments. (A) CCK8 assays showed no toxicity to TM3 Leydig cells at low, medium, and high MCPP concentrations. (B) OPLS-DA of metabolomes between the control and MCPP-treated groups in cells and supernatants, indicating global metabolic changes caused by MCPP treatment. In cells, the R2X (cum), R2Y (cum), and Q2 (cum) are 1, 1, and 0.41, respectively. In cell supernatants, the R2X (cum), R2Y (cum), and Q2 (cum) values are 0.86, 1, and 0.65, respectively, showing that the OPLS-DA model had good fitting and predictability. (C) OPLS-DA of metabolomes between the control and MCPP-treated groups in testis, serum, and urine samples, indicating global metabolic changes caused by MCPP treatment. In testis, the R2X (cum), R2Y (cum), and Q2 (cum) values were 0.73, 1, and 0.45, respectively; in serum, the R2X (cum), R2Y (cum), and Q2 (cum) values were 0.90, 1, and 0.54, respectively; in urine, the R2X (cum), R2Y (cum), and Q2 (cum) values were 0.83, 1, and 0.76, respectively. (D) The activity of CYP11A1 dose-dependently decreased with increasing concentrations in vitro. (E) The activity of CYP11A1 dose-dependently decreased with increasing concentrations in vivo. (F) Relative mRNA levels of Star, Cyp11a1, and Cyp17a1 decreased with increasing concentrations in vivo. *In vitro*: low, medium, and high: 0.01, 1, and 100 μmol/L, respectively. *In vivo*: low, medium, and high: 0.01, 1, and 100 mg/kg, respectively. Data are shown as mean ± SD. “*” indicates *P*<0.05, “**” indicates *P*<0.01.


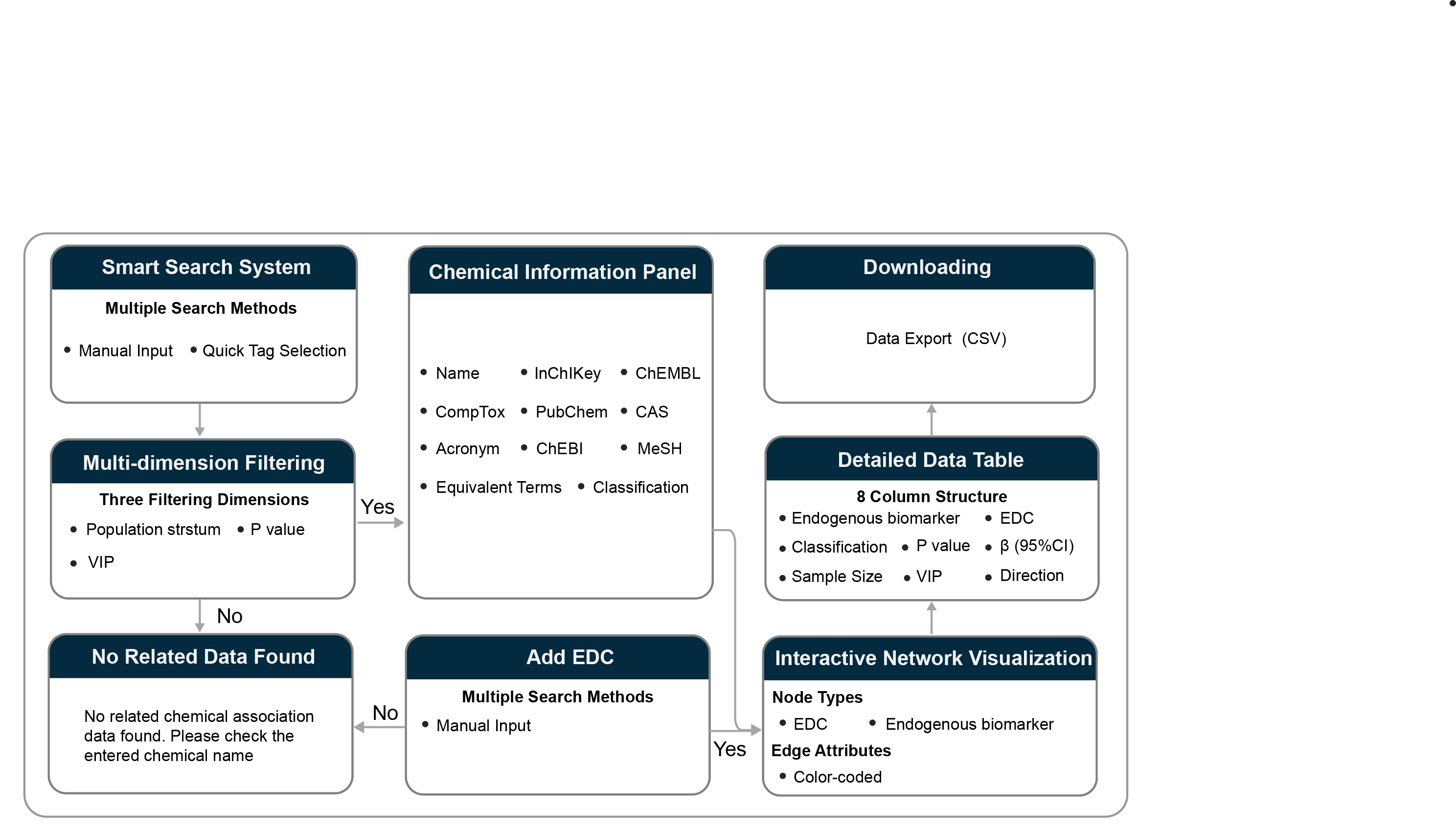


Figure S17. Web portal workflow for exploring EDC–biomarker association landscapes. Schematic of the interactive web portal used to query, filter, and visualize associations between exogenous endocrine-disrupting chemicals (EDCs) and endogenous biomarkers across population strata. Users search for chemicals via the Smart Search System (manual input or tag selection) and refine results using multi-dimensional filtering (population subgroup, *P* value, and variable importance in projection [VIP]). When filter criteria are met, the Chemical Information Panel reports standardized identifiers and database cross-references (e.g., InChIKey, ChEBI, ChEMBL, CompTox, PubChem, CAS, MeSH). An Interactive Network Visualization displays exogenous and endogenous node types, with edge attributes encoding association patterns. The portal further supports iterative inclusion of additional chemicals to update querying and visualization under the same population and filtering settings. Filtered results populate a Detailed Data Table summarizing exogenous–endogenous pairs, classification, effect size (*β*), *P* value, sample size (N), VIP, and association type; results can be exported as CSV.


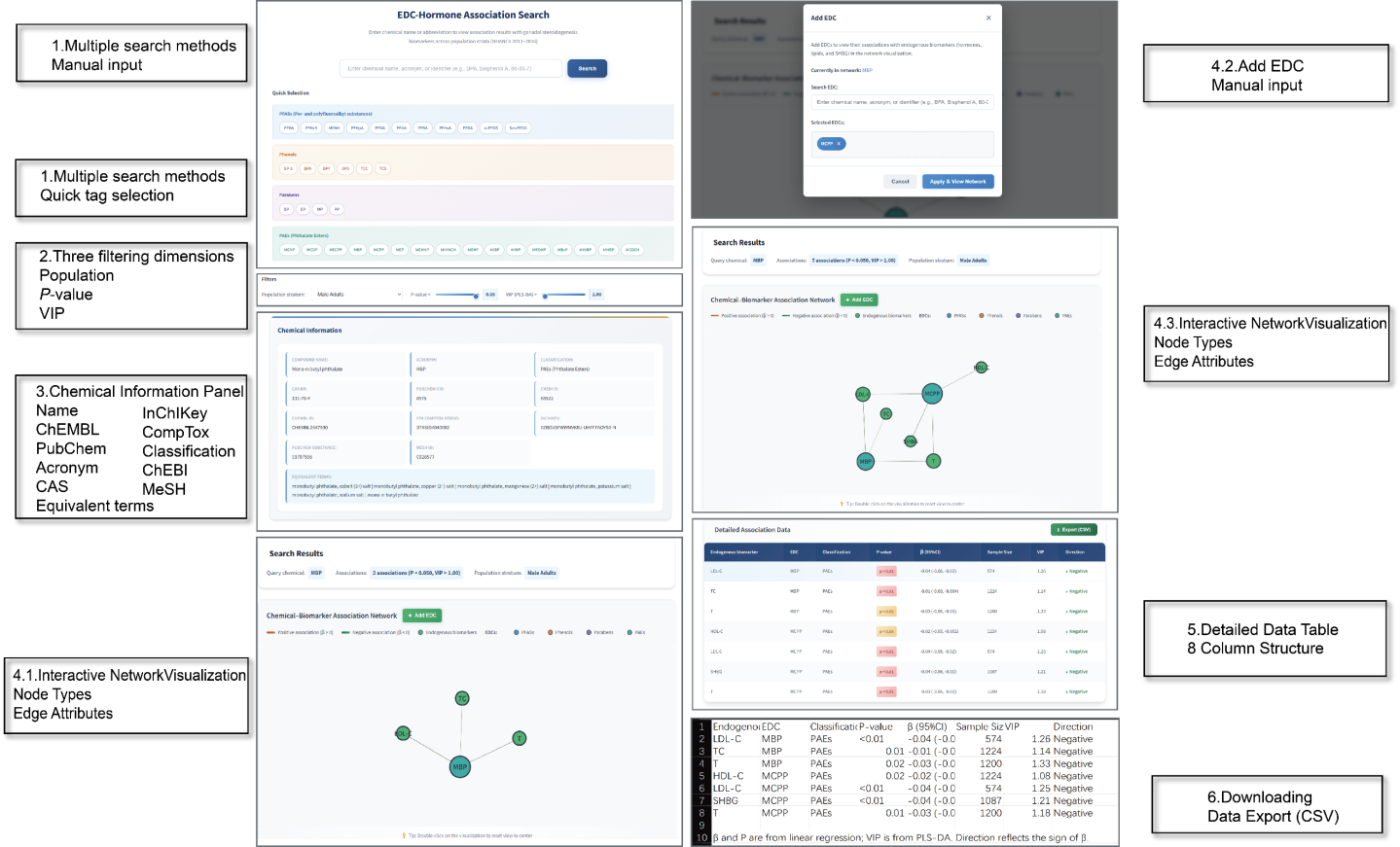


Figure S18. User interface of the web portal for querying and visualizing EDC–biomarker association landscapes. Screenshot showing the end-to-end workflow implemented in the interactive portal. (1) Chemical Association Search enables manual entry of a chemical name/abbreviation and quick class-based tag selection (e.g., PFASs, phenols, PAEs). (2) Multi-dimensional filtering refines results by study population, statistical significance (*P* value), and variable importance in projection (VIP). (3) When filter criteria are met, the Chemical Information panel displays standardized identifiers and database cross-references (e.g., CAS, PubChem, CompTox, ChEBI) for the selected compound (example shown: MBP). (4) Interactive Network Visualization renders the association network between the selected exogenous chemical(s) and endogenous biomarkers (node types encoded by category; edges encode association direction and relative strength as defined in the portal legend). (5) The portal supports iterative inclusion of additional chemicals to expand the network view and compare multi-chemical patterns within the same population and filtering settings(example shown: MCPP). (6) A Detailed Association Data table summarizes significant exogenous–endogenous pairs, including classification, effect size (*β* with 95% CI), sample size (N), *P*-value, VIP, and association direction (MBP and MCPP), with an option to export results.

**
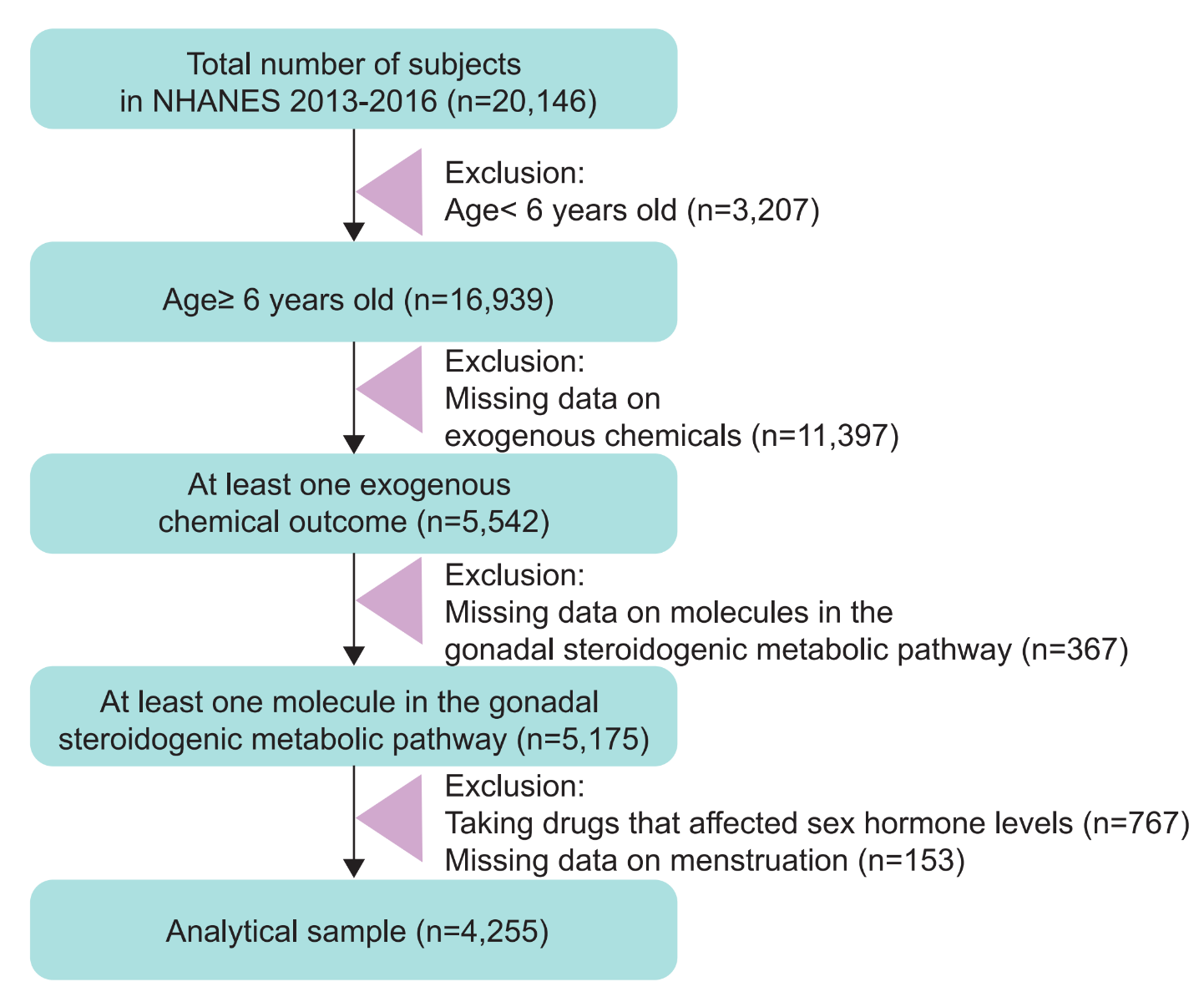
**

Figure S19. Flowchart of participant selection.

**
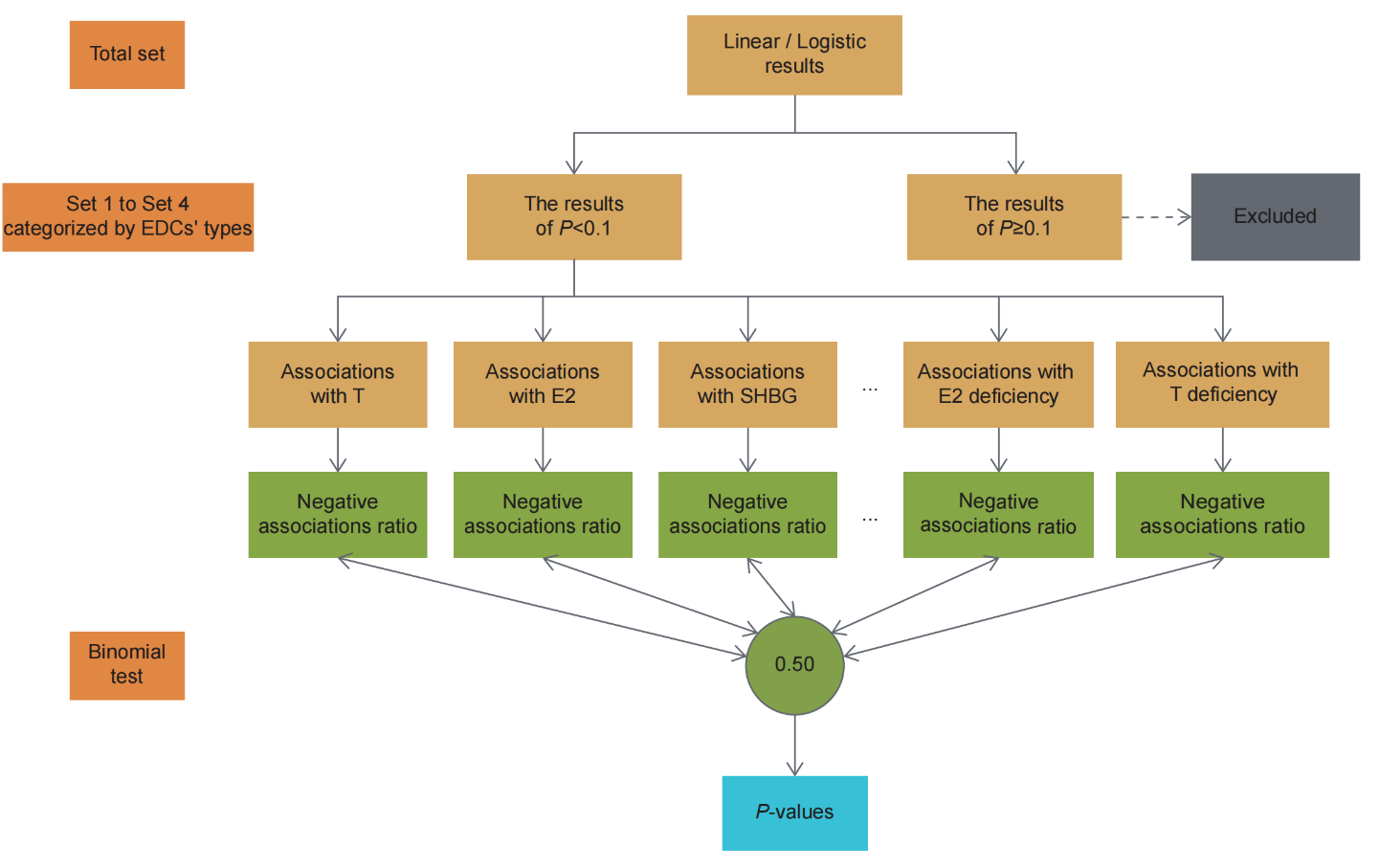
**

Figure S20. Binomial test statistical procedure.

Table S1. Population characteristics of individuals (n=4,255)

| **Characteristics** | **Male children** | **Female children** | **Male adolescents** | **Female adolescents** | **Male adults** | **Female adults** |
| --- | --- | --- | --- | --- | --- | --- |
| Number | 493 | 448 | 412 | 345 | 1,236 | 1,321 |
| Age^a^ | 8.48 (1.71) | 8.61 (1.74) | 15.42 (2.21) | 15.41 (2.25) | 44.27 (16.16) | 46.00 (16.49) |
| Race, n (%) |  |  |  |  |  |  |
| Other | 364 (73.8) | 327 (73.0) | 291 (70.6) | 261 (75.7) | 803 (65.0) | 852 (64.5) |
| Non-Hispanic White | 129 (26.2) | 121 (27.0) | 121 (29.4) | 84 (24.3) | 433 (35.0) | 469 (35.5) |
| BMI, n (%) |  |  |  |  |  |  |
| Abnormal | 327 (66.3) | 279 (62.3) | 210 (51.0) | 174 (50.4) | 867 (70.1) | 934 (70.7) |
| Normal | 166 (33.7) | 169 (37.7) | 201 (48.8) | 165 (47.8) | 356 (28.8) | 379 (28.7) |
| Missing | - | - | 1 (0.2) | 6 (1.7) | 13 (1.1) | 8 (0.6) |
| PIR, n (%) |  |  |  |  |  |  |
| 0-1 | 172 (34.9) | 146 (32.6) | 125 (30.3) | 110 (31.9) | 232 (18.8) | 307 (23.2) |
| 1-3 | 187 (37.9) | 172 (38.4) | 160 (38.8) | 129 (37.4) | 493 (39.9) | 474 (35.9) |
| >3 | 106 (21.5) | 98 (21.9) | 92 (22.3) | 73 (21.2) | 393 (31.8) | 431 (32.6) |
| Missing | 28 (5.7) | 32 (7.1) | 35 (8.5) | 33 (9.6) | 118 (9.5) | 109 (8.3) |
| Serum cotinine level, n (%) |  |  |  |  |  |  |
| <LLOD | 186 (37.7) | 178 (39.7) | 130 (31.6) | 133 (38.6) | 290 (23.5) | 470 (35.6) |
| >LLOD | 306 (62.1) | 269 (60.0) | 282 (68.4) | 212 (61.4) | 946 (76.5) | 851 (64.4) |
| Missing | 1 (0.2) | 1 (0.2) | - | - | - | - |
| Alcohol use, n (%) |  |  |  |  |  |  |
| No | - | - | - | - | 205 (16.6) | 495 (37.5) |
| Yes | - | - | - | - | 939 (76.0) | 824 (62.4) |
| Missing | - | - | - | - | 92 (7.4) | 2 (0.2) |
| Six-month period when the examination was performed, n (%) |  |  |  |  |  |  |
| November 1 through April 30 | 255 (51.7) | 212 (47.3) | 212 (51.5) | 177 (51.3) | 589 (47.7) | 657 (49.7) |
| May 1 through October 31 | 238 (48.3) | 236 (52.7) | 200 (48.5) | 168 (48.7) | 647 (52.3) | 664 (50.3) |
| Session time of venipuncture, n (%) |  |  |  |  |  |  |
| morning | 186 (37.7) | 176 (39.3) | 192 (46.6) | 173 (50.1) | 609 (49.3) | 619 (46.9) |
| afternoon | 197 (40.0) | 178 (39.7) | 142 (34.5) | 104 (30.1) | 426 (34.5) | 488 (36.9) |
| evening | 110 (22.3) | 94 (21.0) | 78 (18.9) | 68 (19.7) | 201 (16.3) | 214 (16.2) |
| T deficiency, n (%) |  |  |  |  |  |  |
| No | - | - | - | - | 917 (74.2) | - |
| Yes | - | - | - | - | 276 (22.3) | - |
| Missing | - | - | - | - | 43 (3.5) | - |
| Abnormally high T, n (%) |  |  |  |  |  |  |
| No | - | - | - | - | 917 (74.2) | - |
| Yes | - | - | - | - | 19 (1.5) | - |
| Missing | - | - | - | - | 300 (24.3) | - |
| E2 deficiency, n (%) |  |  |  |  |  |  |
| No | - | - | - | - | 1139 (92.2) | - |
| Yes | - | - | - | - | 23 (1.9) | - |
| Missing | - | - | - | - | 74 (6.0) | - |
| Abnormally high E2, n (%) |  |  |  |  |  |  |
| No | - | - | - | - | 1139 (92.2) | - |
| Yes | - | - | - | - | 31 (2.5) | - |
| Missing | - | - | - | - | 66 (5.3) | - |
| T (ng/dL)^a^ | 22.14 (66.55) | 7.75 (7.91) | 382.49 (201.26) | 28.35 (18.86) | 441.63 (198.65) | 25.84 (32.25) |
| E2 (pg/mL)^a^ | 2.68 (2.29) | 12.28 (23.56) | 19.45 (10.36) | 131.00 (757.12) | 25.53 (10.11) | 141.23 (883.16) |
| SHBG (nmol/L)^a^ | 98.20 (47.30) | 80.72 (42.38) | 37.70 (22.22) | 61.84 (58.09) | 43.20 (24.92) | 77.93 (74.54) |
| HDL-C (mg/dL)^a^ | - | - | - | - | 48.91 (15.64) | 58.38 (16.90) |
| LDL-C (mg/dL)^a^ | - | - | - | - | 118.14 (34.06) | 114.28 (35.13) |
| TC (mg/dL)^a^ | - | - | - | - | 193.51 (40.74) | 195.45 (38.94) |
| TG (mg/dL)^a^ | - | - | - | - | 128.84 (123.84) | 102.76 (63.41) |

a: Variables are presented as means ± standard deviation (SD)

Table S2. Concentrations and detection frequencies of chemicals in childhood

| **Classific ations** | **Chemicals (ng/ml)** | **Male children** | | | | | | | | | **Female children** | | | | | | | | |
| --- | --- | --- | --- | --- | --- | --- | --- | --- | --- | --- | --- | --- | --- | --- | --- | --- | --- | --- | --- |
|  |  | **N** | **Detection rate** | **5%** | **10%** | **25%** | **50%** | **75%** | **90%** | **95%** | **N** | **Detection rate** | **5%** | **10%** | **25%** | **50%** | **75%** | **90%** | **95%** |
| PAEs | MBP | 347 | 99.71% | 2.96 | 4.26 | 7.75 | 14.80 | 24.95 | 40.26 | 56.69 | 336 | 98.81% | 2.28 | 3.95 | 7.18 | 13.95 | 27.45 | 44.90 | 60.03 |
| PAEs | MBzP | 347 | 99.71% | 1.43 | 2.16 | 4.70 | 10.90 | 26.45 | 60.92 | 90.89 | 336 | 99.40% | 1.00 | 1.75 | 4.00 | 8.85 | 20.23 | 36.10 | 54.25 |
| PAEs | MCNP | 347 | 99.42% | 0.60 | 0.80 | 1.50 | 2.60 | 5.10 | 9.18 | 15.25 | 336 | 99.70% | 0.60 | 0.85 | 1.40 | 2.80 | 4.60 | 7.65 | 11.33 |
| PAEs | MCOCH | 182 | 71.43% | <LOD | <LOD | <LOD | 0.70 | 1.18 | 2.10 | 5.57 | 161 | 71.43% | <LOD | <LOD | <LOD | 0.80 | 1.40 | 3.70 | 5.10 |
| PAEs | MCOP | 347 | 100.00% | 2.60 | 3.62 | 7.40 | 14.60 | 35.45 | 92.78 | 179.23 | 336 | 99.70% | 2.60 | 3.90 | 6.88 | 14.75 | 34.65 | 82.65 | 151.00 |
| PAEs | MCPP | 347 | 92.80% | <LOD | 0.60 | 1.20 | 2.20 | 4.70 | 8.92 | 15.55 | 336 | 92.26% | <LOD | 0.50 | 1.10 | 2.00 | 4.00 | 8.10 | 12.83 |
| PAEs | MECPP | 347 | 100.00% | 3.63 | 5.16 | 9.75 | 17.00 | 29.80 | 49.26 | 72.61 | 336 | 100.00% | 3.48 | 4.90 | 8.80 | 16.50 | 30.05 | 53.35 | 71.80 |
| PAEs | MEHHP | 347 | 99.42% | 2.10 | 2.96 | 5.25 | 10.30 | 18.10 | 29.68 | 43.50 | 336 | 100.00% | 1.70 | 2.50 | 4.70 | 9.15 | 16.95 | 34.05 | 49.75 |
| PAEs | MEHP | 347 | 68.01% | <LOD | <LOD | <LOD | 1.50 | 2.95 | 4.74 | 8.31 | 336 | 66.67% | <LOD | <LOD | <LOD | 1.25 | 2.80 | 4.80 | 7.68 |
| PAEs | MEOHP | 347 | 100.00% | 1.43 | 2.00 | 3.70 | 6.90 | 12.10 | 18.76 | 27.96 | 336 | 100.00% | 1.20 | 1.75 | 3.10 | 6.40 | 11.50 | 22.00 | 33.08 |
| PAEs | MEP | 347 | 99.71% | 4.62 | 6.88 | 12.70 | 29.10 | 59.30 | 142.78 | 221.88 | 336 | 99.40% | 3.45 | 5.50 | 10.90 | 26.15 | 55.90 | 126.60 | 267.38 |
| PAEs | MHBP | 347 | 85.17% | <LOD | <LOD | 0.60 | 1.30 | 2.30 | 3.94 | 5.20 | 336 | 84.52% | <LOD | <LOD | 0.60 | 1.30 | 2.50 | 4.20 | 5.93 |
| PAEs | MHiBP | 347 | 97.69% | 0.70 | 1.10 | 2.25 | 4.10 | 7.50 | 13.60 | 18.21 | 336 | 97.62% | 0.70 | 1.10 | 2.10 | 4.50 | 8.33 | 14.55 | 23.80 |
| PAEs | MHINCH | 347 | 71.76% | <LOD | <LOD | <LOD | 0.80 | 1.50 | 3.10 | 4.67 | 336 | 75.00% | <LOD | <LOD | 0.37 | 0.90 | 1.80 | 3.60 | 6.40 |
| PAEs | MiBP | 347 | 98.56% | 1.73 | 3.02 | 5.50 | 11.10 | 20.80 | 35.98 | 51.67 | 336 | 98.51% | 1.60 | 2.95 | 5.38 | 11.50 | 21.65 | 40.85 | 64.03 |
| PAEs | MiNP | 347 | 38.33% | <LOD | <LOD | <LOD | <LOD | 1.40 | 4.48 | 8.90 | 336 | 37.50% | <LOD | <LOD | <LOD | <LOD | 1.40 | 3.40 | 9.25 |
| Parabens | BP | 347 | 15.56% | <LOD | <LOD | <LOD | <LOD | <LOD | 0.10 | 0.27 | 336 | 28.57% | <LOD | <LOD | <LOD | <LOD | 0.10 | 0.20 | 0.50 |
| Parabens | EP | 347 | 30.26% | <LOD | <LOD | <LOD | <LOD | 1.10 | 3.08 | 8.99 | 336 | 36.90% | <LOD | <LOD | <LOD | <LOD | 1.60 | 7.15 | 25.78 |
| Parabens | MP | 347 | 97.98% | 2.36 | 3.60 | 7.05 | 17.90 | 61.05 | 229.76 | 550.14 | 336 | 98.81% | 2.15 | 4.15 | 8.60 | 27.20 | 106.60 | 344.55 | 652.65 |
| Parabens | PP | 347 | 98.56% | 0.30 | 0.40 | 0.70 | 1.70 | 4.90 | 40.62 | 79.64 | 336 | 98.81% | 0.20 | 0.40 | 1.00 | 3.40 | 19.30 | 66.25 | 122.60 |
| PFASs | MPAH | 201 | 44.78% | <LOD | <LOD | <LOD | <LOD | 0.25 | 0.70 | 1.37 | 171 | 47.95% | <LOD | <LOD | <LOD | <LOD | 0.22 | 0.40 | 0.65 |
| PFASs | n-PFOS | 201 | 100.00% | 0.99 | 1.16 | 1.79 | 2.73 | 4.03 | 5.91 | 9.04 | 171 | 100.00% | 1.12 | 1.38 | 1.72 | 2.22 | 3.19 | 5.06 | 5.95 |
| PFASs | PFBA | 165 | 9.70% | <LOD | <LOD | <LOD | <LOD | <LOD | <LOD | 0.20 | 175 | 10.86% | <LOD | <LOD | <LOD | <LOD | <LOD | 0.10 | 0.23 |
| PFASs | PFDA | 201 | 44.78% | <LOD | <LOD | <LOD | <LOD | 0.18 | 0.26 | 0.32 | 171 | 49.12% | <LOD | <LOD | <LOD | <LOD | 0.18 | 0.31 | 0.37 |
| PFASs | PFHpA | 201 | 13.93% | <LOD | <LOD | <LOD | <LOD | <LOD | 0.12 | 0.15 | 171 | 16.37% | <LOD | <LOD | <LOD | <LOD | <LOD | 0.14 | 0.18 |
| PFASs | PFHxA | 165 | 23.64% | <LOD | <LOD | <LOD | <LOD | <LOD | 0.30 | 0.50 | 175 | 18.86% | <LOD | <LOD | <LOD | <LOD | <LOD | 0.26 | 0.40 |
| PFASs | PFHxS | 201 | 100.00% | 0.30 | 0.37 | 0.53 | 0.81 | 1.41 | 2.63 | 4.59 | 171 | 99.42% | 0.30 | 0.33 | 0.47 | 0.67 | 0.99 | 1.97 | 2.81 |
| PFASs | PFNA | 201 | 100.00% | 0.36 | 0.41 | 0.50 | 0.77 | 1.17 | 2.24 | 3.74 | 171 | 99.42% | 0.36 | 0.40 | 0.50 | 0.71 | 1.04 | 1.82 | 2.71 |
| PFASs | PFOA | 201 | 100.00% | 0.84 | 0.94 | 1.24 | 1.70 | 2.39 | 3.20 | 3.64 | 171 | 100.00% | 0.80 | 0.93 | 1.18 | 1.74 | 2.27 | 2.84 | 3.71 |
| PFASs | PFUA | 201 | 32.84% | <LOD | <LOD | <LOD | <LOD | 0.12 | 0.19 | 0.26 | 171 | 33.92% | <LOD | <LOD | <LOD | <LOD | 0.12 | 0.24 | 0.30 |
| PFASs | Sm-PFOS | 201 | 100.00% | 0.36 | 0.47 | 0.80 | 1.31 | 2.05 | 3.41 | 4.10 | 171 | 100.00% | 0.38 | 0.50 | 0.80 | 1.08 | 1.66 | 2.70 | 3.28 |
| Phenols | BP-3 | 347 | 97.69% | 1.23 | 1.90 | 4.80 | 11.80 | 33.30 | 135.06 | 356.53 | 336 | 99.11% | 1.45 | 2.55 | 6.30 | 15.70 | 49.30 | 159.20 | 469.85 |
| Phenols | BPA | 347 | 97.12% | 0.30 | 0.40 | 0.70 | 1.40 | 2.70 | 4.94 | 7.27 | 336 | 97.02% | 0.30 | 0.40 | 0.70 | 1.20 | 2.20 | 4.30 | 5.58 |
| Phenols | BPF | 347 | 48.41% | <LOD | <LOD | <LOD | <LOD | 0.60 | 1.90 | 5.48 | 336 | 51.79% | <LOD | <LOD | <LOD | 0.20 | 0.60 | 1.55 | 3.13 |
| Phenols | BPS | 347 | 89.91% | <LOD | <LOD | 0.20 | 0.40 | 0.80 | 1.70 | 3.58 | 336 | 88.99% | <LOD | <LOD | 0.20 | 0.40 | 0.70 | 1.60 | 3.23 |
| Phenols | TCC | 347 | 36.60% | <LOD | <LOD | <LOD | <LOD | 0.20 | 1.18 | 10.24 | 336 | 26.49% | <LOD | <LOD | <LOD | <LOD | 0.10 | 0.30 | 1.38 |
| Phenols | TCS | 347 | 71.76% | <LOD | <LOD | <LOD | 3.80 | 18.50 | 62.14 | 140.93 | 336 | 75.30% | <LOD | <LOD | 1.70 | 3.75 | 15.33 | 64.35 | 130.20 |

Table S3. Concentrations and detection frequencies of chemicals in adolescents

| **Classific ations** | **Chemicals (ng/ml)** | **Male adolescents** | | | | | | | | | **Female adolescents** | | | | | | | | |
| --- | --- | --- | --- | --- | --- | --- | --- | --- | --- | --- | --- | --- | --- | --- | --- | --- | --- | --- | --- |
|  |  | **N** | **Detection rate** | **5%** | **10%** | **25%** | **50%** | **75%** | **90%** | **95%** | **N** | **Detection rate** | **5%** | **10%** | **25%** | **50%** | **75%** | **90%** | **95%** |
| PAEs | MBP | 407 | 99.75% | 2.90 | 3.90 | 6.60 | 12.80 | 22.15 | 40.32 | 56.86 | 340 | 98.82% | 1.70 | 2.60 | 6.18 | 12.60 | 23.95 | 43.87 | 59.85 |
| PAEs | MBzP | 407 | 99.26% | 1.00 | 1.60 | 3.10 | 6.90 | 15.40 | 27.82 | 44.97 | 340 | 98.24% | 0.50 | 0.99 | 2.40 | 6.00 | 14.25 | 25.31 | 45.55 |
| PAEs | MCNP | 407 | 99.51% | 0.60 | 0.80 | 1.50 | 2.50 | 4.40 | 8.08 | 13.97 | 340 | 99.12% | 0.40 | 0.59 | 1.20 | 2.30 | 4.45 | 10.40 | 16.43 |
| PAEs | MCOCH | 200 | 63.00% | <LOD | <LOD | <LOD | 0.60 | 1.00 | 2.70 | 5.71 | 151 | 63.58% | <LOD | <LOD | <LOD | 0.60 | 0.95 | 1.90 | 3.70 |
| PAEs | MCOP | 407 | 99.75% | 2.43 | 3.26 | 6.20 | 15.20 | 43.50 | 123.50 | 175.95 | 340 | 100.00% | 1.60 | 2.59 | 5.40 | 13.75 | 37.13 | 110.33 | 194.31 |
| PAEs | MCPP | 407 | 89.19% | <LOD | <LOD | 0.85 | 1.70 | 3.60 | 7.30 | 14.09 | 340 | 83.24% | <LOD | <LOD | 0.60 | 1.40 | 3.40 | 8.71 | 19.53 |
| PAEs | MECPP | 407 | 100.00% | 2.80 | 3.96 | 7.05 | 12.40 | 19.55 | 30.70 | 47.64 | 340 | 99.71% | 2.10 | 2.70 | 5.10 | 11.65 | 22.78 | 40.48 | 49.85 |
| PAEs | MEHHP | 407 | 99.51% | 1.60 | 2.40 | 4.20 | 7.30 | 13.05 | 20.92 | 28.69 | 340 | 98.53% | 1.00 | 1.49 | 2.78 | 6.65 | 13.20 | 23.85 | 31.42 |
| PAEs | MEHP | 407 | 66.34% | <LOD | <LOD | <LOD | 1.10 | 2.50 | 4.68 | 6.10 | 340 | 59.71% | <LOD | <LOD | <LOD | 1.10 | 2.80 | 4.81 | 6.91 |
| PAEs | MEOHP | 407 | 99.75% | 1.00 | 1.60 | 2.75 | 4.70 | 8.30 | 12.70 | 18.69 | 340 | 99.71% | 0.70 | 1.00 | 2.00 | 4.70 | 9.35 | 15.90 | 21.59 |
| PAEs | MEP | 407 | 100.00% | 5.50 | 8.72 | 16.10 | 34.00 | 74.35 | 221.16 | 458.04 | 340 | 99.41% | 4.30 | 6.18 | 15.38 | 30.25 | 85.25 | 226.71 | 374.03 |
| PAEs | MHBP | 407 | 81.57% | <LOD | <LOD | 0.50 | 1.00 | 1.90 | 3.34 | 4.51 | 339 | 78.47% | <LOD | <LOD | 0.50 | 1.00 | 2.15 | 3.50 | 4.31 |
| PAEs | MHiBP | 407 | 97.79% | 0.63 | 1.00 | 1.70 | 3.60 | 6.00 | 11.12 | 14.97 | 339 | 96.46% | 0.50 | 0.88 | 1.90 | 3.70 | 7.30 | 15.00 | 21.02 |
| PAEs | MHINCH | 407 | 56.76% | <LOD | <LOD | <LOD | 0.50 | 1.00 | 2.40 | 5.60 | 340 | 51.76% | <LOD | <LOD | <LOD | 0.40 | 1.00 | 2.01 | 3.61 |
| PAEs | MiBP | 407 | 99.75% | 2.00 | 2.90 | 5.35 | 10.20 | 18.60 | 31.94 | 43.24 | 340 | 98.82% | 1.60 | 2.78 | 4.80 | 9.90 | 20.60 | 39.47 | 59.47 |
| PAEs | MiNP | 407 | 42.01% | <LOD | <LOD | <LOD | <LOD | 1.65 | 4.80 | 9.83 | 340 | 35.59% | <LOD | <LOD | <LOD | <LOD | 1.50 | 5.34 | 11.55 |
| Parabens | BP | 407 | 14.50% | <LOD | <LOD | <LOD | <LOD | <LOD | 0.10 | 0.20 | 340 | 37.06% | <LOD | <LOD | <LOD | <LOD | 0.20 | 1.31 | 3.40 |
| Parabens | EP | 407 | 27.52% | <LOD | <LOD | <LOD | <LOD | 1.20 | 3.34 | 7.20 | 340 | 50.88% | <LOD | <LOD | <LOD | 1.10 | 4.23 | 25.81 | 54.25 |
| Parabens | MP | 407 | 98.77% | 2.23 | 3.16 | 6.40 | 16.10 | 66.25 | 364.54 | 732.32 | 340 | 98.82% | 3.09 | 4.30 | 12.93 | 55.95 | 224.48 | 531.96 | 1105.81 |
| Parabens | PP | 407 | 99.02% | 0.20 | 0.30 | 0.65 | 1.70 | 9.50 | 49.74 | 123.34 | 340 | 99.71% | 0.40 | 0.60 | 1.90 | 6.85 | 38.00 | 123.88 | 279.00 |
| PFASs | MPAH | 402 | 47.51% | <LOD | <LOD | <LOD | <LOD | 0.20 | 0.30 | 0.60 | 337 | 37.69% | <LOD | <LOD | <LOD | <LOD | 0.10 | 0.30 | 0.50 |
| PFASs | n-PFOS | 402 | 99.75% | 1.00 | 1.20 | 1.70 | 2.40 | 3.68 | 5.28 | 7.10 | 336 | 100.00% | 0.80 | 0.90 | 1.30 | 1.80 | 2.70 | 4.60 | 5.58 |
| PFASs | PFBA | 207 | 14.49% | <LOD | <LOD | <LOD | <LOD | <LOD | 0.10 | 0.30 | 188 | 10.11% | <LOD | <LOD | <LOD | <LOD | <LOD | 0.08 | 0.27 |
| PFASs | PFDA | 402 | 63.18% | <LOD | <LOD | <LOD | 0.10 | 0.20 | 0.30 | 0.40 | 337 | 61.13% | <LOD | <LOD | <LOD | 0.10 | 0.20 | 0.30 | 0.40 |
| PFASs | PFHpA | 208 | 23.56% | <LOD | <LOD | <LOD | <LOD | <LOD | 0.10 | 0.20 | 185 | 15.68% | <LOD | <LOD | <LOD | <LOD | <LOD | 0.10 | 0.20 |
| PFASs | PFHxA | 207 | 24.64% | <LOD | <LOD | <LOD | <LOD | <LOD | 0.30 | 0.40 | 188 | 23.94% | <LOD | <LOD | <LOD | <LOD | <LOD | 0.23 | 0.53 |
| PFASs | PFHxS | 402 | 99.50% | 0.40 | 0.50 | 0.70 | 1.00 | 1.80 | 3.59 | 5.20 | 337 | 100.00% | 0.20 | 0.30 | 0.50 | 0.70 | 1.30 | 1.90 | 2.72 |
| PFASs | PFNA | 402 | 100.00% | 0.20 | 0.30 | 0.40 | 0.50 | 0.80 | 1.30 | 1.60 | 337 | 99.70% | 0.20 | 0.20 | 0.30 | 0.50 | 0.70 | 1.04 | 1.30 |
| PFASs | PFOA | 402 | 99.75% | 0.70 | 0.90 | 1.10 | 1.40 | 1.90 | 2.60 | 3.10 | 336 | 100.00% | 0.50 | 0.60 | 0.80 | 1.10 | 1.53 | 2.20 | 2.63 |
| PFASs | PFUA | 402 | 19.90% | <LOD | <LOD | <LOD | <LOD | <LOD | 0.20 | 0.30 | 337 | 19.88% | <LOD | <LOD | <LOD | <LOD | <LOD | 0.20 | 0.22 |
| PFASs | Sm-PFOS | 402 | 48.13% | <LOD | <LOD | <LOD | <LOD | 1.30 | 1.90 | 2.20 | 336 | 44.71% | <LOD | <LOD | <LOD | <LOD | 0.93 | 1.20 | 1.50 |
| Phenols | BP-3 | 407 | 98.28% | 1.23 | 2.36 | 5.05 | 11.30 | 34.65 | 112.62 | 254.41 | 340 | 98.82% | 2.50 | 3.69 | 7.88 | 22.75 | 64.93 | 197.93 | 370.58 |
| Phenols | BPA | 407 | 97.30% | 0.30 | 0.40 | 0.70 | 1.20 | 2.30 | 4.40 | 7.07 | 340 | 98.24% | 0.30 | 0.40 | 0.60 | 1.30 | 2.30 | 4.91 | 7.31 |
| Phenols | BPF | 407 | 56.51% | <LOD | <LOD | <LOD | 0.30 | 1.00 | 5.60 | 16.45 | 339 | 55.75% | <LOD | <LOD | <LOD | 0.30 | 0.80 | 3.10 | 9.95 |
| Phenols | BPS | 407 | 89.43% | <LOD | <LOD | 0.20 | 0.40 | 0.90 | 2.20 | 3.20 | 339 | 87.02% | <LOD | <LOD | 0.20 | 0.40 | 0.90 | 2.10 | 3.02 |
| Phenols | TCC | 407 | 33.91% | <LOD | <LOD | <LOD | <LOD | 0.20 | 1.30 | 6.07 | 340 | 35.29% | <LOD | <LOD | <LOD | <LOD | 0.20 | 0.91 | 3.55 |
| Phenols | TCS | 407 | 75.43% | <LOD | <LOD | 1.70 | 4.10 | 17.45 | 109.18 | 301.93 | 340 | 71.18% | <LOD | <LOD | <LOD | 5.15 | 18.13 | 179.39 | 522.77 |

Table S4. Concentrations and detection frequencies of chemicals in adults

| **Classific ations** | **Chemicals (ng/ml)** | **Male adults** | | | | | | | | | **Female adults** | | | | | | | | |
| --- | --- | --- | --- | --- | --- | --- | --- | --- | --- | --- | --- | --- | --- | --- | --- | --- | --- | --- | --- |
|  |  | **N** | **Detection rate** | **5%** | **10%** | **25%** | **50%** | **75%** | **90%** | **95%** | **N** | **Detection rate** | **5%** | **10%** | **25%** | **50%** | **75%** | **90%** | **95%** |
| PAEs | MBP | 1224 | 98.86% | 1.40 | 2.63 | 5.20 | 10.90 | 20.10 | 34.08 | 48.99 | 1447 | 98.06% | 1.20 | 2.10 | 5.10 | 10.70 | 20.60 | 38.48 | 53.34 |
| PAEs | MBzP | 1224 | 97.55% | 0.50 | 0.90 | 1.90 | 4.40 | 10.53 | 21.87 | 35.97 | 1447 | 96.89% | 0.40 | 0.70 | 1.60 | 4.00 | 10.10 | 22.58 | 35.97 |
| PAEs | MCNP | 1224 | 97.96% | 0.40 | 0.60 | 1.20 | 2.20 | 4.30 | 8.90 | 14.93 | 1447 | 97.30% | 0.30 | 0.50 | 0.90 | 1.80 | 3.70 | 7.30 | 11.64 |
| PAEs | MCOCH | 599 | 48.08% | <LOD | <LOD | <LOD | <LOD | 0.80 | 2.02 | 3.31 | 704 | 53.27% | <LOD | <LOD | <LOD | 0.50 | 0.80 | 2.00 | 4.40 |
| PAEs | MCOP | 1224 | 99.67% | 1.60 | 2.30 | 4.80 | 11.75 | 32.63 | 93.74 | 170.85 | 1447 | 99.45% | 1.30 | 2.10 | 4.30 | 10.40 | 30.00 | 82.84 | 154.30 |
| PAEs | MCPP | 1224 | 84.80% | <LOD | <LOD | 0.70 | 1.40 | 3.50 | 8.50 | 15.97 | 1447 | 80.58% | <LOD | <LOD | 0.50 | 1.20 | 3.00 | 7.40 | 14.17 |
| PAEs | MECPP | 1224 | 99.51% | 1.72 | 2.80 | 5.50 | 10.60 | 18.73 | 32.87 | 52.51 | 1447 | 99.72% | 1.80 | 2.46 | 4.80 | 9.50 | 18.65 | 33.70 | 46.47 |
| PAEs | MEHHP | 1224 | 99.10% | 1.10 | 1.80 | 3.50 | 7.10 | 13.10 | 23.87 | 35.99 | 1447 | 99.59% | 1.00 | 1.40 | 2.90 | 6.10 | 12.10 | 22.70 | 32.61 |
| PAEs | MEHP | 1224 | 64.95% | <LOD | <LOD | <LOD | 1.40 | 2.70 | 5.07 | 7.70 | 1447 | 59.78% | <LOD | <LOD | <LOD | 1.10 | 2.50 | 4.80 | 7.60 |
| PAEs | MEOHP | 1224 | 98.94% | 0.60 | 1.10 | 2.10 | 4.40 | 7.90 | 14.40 | 20.37 | 1447 | 99.31% | 0.60 | 0.90 | 1.90 | 4.00 | 8.00 | 14.50 | 19.94 |
| PAEs | MEP | 1224 | 99.84% | 4.62 | 6.83 | 14.10 | 33.25 | 103.23 | 338.97 | 745.49 | 1447 | 99.59% | 4.20 | 7.20 | 15.40 | 39.20 | 108.80 | 300.58 | 561.38 |
| PAEs | MHBP | 1223 | 70.56% | <LOD | <LOD | <LOD | 0.70 | 1.50 | 2.90 | 4.29 | 1445 | 71.21% | <LOD | <LOD | <LOD | 0.90 | 1.70 | 3.40 | 4.50 |
| PAEs | MHiBP | 1223 | 95.26% | 0.40 | 0.70 | 1.40 | 2.80 | 5.30 | 9.80 | 15.50 | 1445 | 93.63% | <LOD | 0.60 | 1.30 | 2.90 | 5.90 | 10.40 | 15.68 |
| PAEs | MHINCH | 1224 | 38.64% | <LOD | <LOD | <LOD | <LOD | 0.70 | 1.80 | 3.69 | 1447 | 40.22% | <LOD | <LOD | <LOD | <LOD | 0.70 | 1.80 | 3.97 |
| PAEs | MiBP | 1224 | 98.20% | 1.40 | 2.33 | 4.40 | 9.10 | 17.50 | 30.07 | 48.77 | 1447 | 97.17% | 1.10 | 1.80 | 3.90 | 8.90 | 17.30 | 31.42 | 49.51 |
| PAEs | MiNP | 1224 | 37.17% | <LOD | <LOD | <LOD | <LOD | 1.43 | 4.67 | 10.40 | 1447 | 31.31% | <LOD | <LOD | <LOD | <LOD | 1.20 | 3.80 | 8.90 |
| Parabens | BP | 1224 | 15.44% | <LOD | <LOD | <LOD | <LOD | <LOD | 0.20 | 0.50 | 1448 | 43.72% | <LOD | <LOD | <LOD | <LOD | 0.40 | 3.23 | 13.15 |
| Parabens | EP | 1224 | 44.12% | <LOD | <LOD | <LOD | <LOD | 3.13 | 15.79 | 48.08 | 1448 | 59.39% | <LOD | <LOD | <LOD | 1.80 | 13.60 | 67.29 | 144.85 |
| Parabens | MP | 1224 | 98.28% | 2.30 | 3.90 | 9.00 | 25.90 | 94.93 | 325.67 | 697.70 | 1448 | 99.24% | 4.60 | 8.17 | 25.90 | 88.65 | 285.08 | 646.96 | 991.31 |
| Parabens | PP | 1224 | 97.06% | 0.20 | 0.30 | 0.60 | 1.90 | 13.43 | 64.40 | 146.63 | 1448 | 99.24% | 0.30 | 0.70 | 3.08 | 16.15 | 63.33 | 179.15 | 318.49 |
| PFASs | MPAH | 1221 | 41.20% | <LOD | <LOD | <LOD | <LOD | 0.20 | 0.40 | 0.60 | 1449 | 36.16% | <LOD | <LOD | <LOD | <LOD | 0.10 | 0.30 | 0.50 |
| PFASs | n-PFOS | 1220 | 99.51% | 1.30 | 1.80 | 2.70 | 4.30 | 7.30 | 11.51 | 17.90 | 1448 | 99.10% | 0.70 | 0.90 | 1.50 | 2.65 | 4.53 | 8.40 | 13.00 |
| PFASs | PFBA | 624 | 10.74% | <LOD | <LOD | <LOD | <LOD | <LOD | 0.10 | 0.20 | 742 | 9.97% | <LOD | <LOD | <LOD | <LOD | <LOD | <LOD | 0.20 |
| PFASs | PFDA | 1221 | 78.62% | <LOD | <LOD | 0.10 | 0.20 | 0.30 | 0.60 | 0.90 | 1449 | 69.57% | <LOD | <LOD | <LOD | 0.20 | 0.30 | 0.50 | 0.80 |
| PFASs | PFHpA | 625 | 9.76% | <LOD | <LOD | <LOD | <LOD | <LOD | <LOD | 0.10 | 742 | 9.70% | <LOD | <LOD | <LOD | <LOD | <LOD | <LOD | 0.10 |
| PFASs | PFHxA | 624 | 23.24% | <LOD | <LOD | <LOD | <LOD | <LOD | 0.37 | 0.60 | 742 | 23.58% | <LOD | <LOD | <LOD | <LOD | <LOD | 0.30 | 0.40 |
| PFASs | PFHxS | 1221 | 99.02% | 0.50 | 0.80 | 1.20 | 1.70 | 2.70 | 4.20 | 5.60 | 1449 | 98.14% | 0.20 | 0.30 | 0.40 | 0.80 | 1.50 | 2.60 | 3.60 |
| PFASs | PFNA | 1221 | 98.77% | 0.30 | 0.30 | 0.50 | 0.70 | 1.10 | 1.60 | 2.10 | 1449 | 98.34% | 0.20 | 0.20 | 0.40 | 0.60 | 0.90 | 1.40 | 1.90 |
| PFASs | PFOA | 1220 | 99.26% | 0.70 | 0.90 | 1.40 | 2.00 | 2.80 | 3.91 | 5.10 | 1448 | 99.03% | 0.30 | 0.50 | 0.80 | 1.30 | 2.10 | 3.30 | 4.20 |
| PFASs | PFUA | 1221 | 44.64% | <LOD | <LOD | <LOD | <LOD | 0.20 | 0.40 | 0.60 | 1449 | 41.75% | <LOD | <LOD | <LOD | <LOD | 0.20 | 0.30 | 0.60 |
| PFASs | Sm-PFOS | 1220 | 48.28% | <LOD | <LOD | <LOD | <LOD | 3.10 | 4.40 | 5.70 | 1448 | 47.96% | <LOD | <LOD | <LOD | <LOD | 1.70 | 3.10 | 4.27 |
| Phenols | BP-3 | 1224 | 95.42% | 0.60 | 1.50 | 3.90 | 10.80 | 34.15 | 147.34 | 317.83 | 1448 | 95.99% | 0.74 | 2.10 | 5.80 | 20.75 | 98.23 | 530.32 | 1574.86 |
| Phenols | BPA | 1224 | 95.26% | 0.20 | 0.40 | 0.60 | 1.30 | 2.70 | 5.27 | 8.77 | 1448 | 93.65% | <LOD | 0.30 | 0.50 | 1.10 | 2.30 | 4.10 | 6.57 |
| Phenols | BPF | 1223 | 56.91% | <LOD | <LOD | <LOD | 0.30 | 0.90 | 3.50 | 9.70 | 1446 | 54.01% | <LOD | <LOD | <LOD | 0.20 | 0.80 | 2.80 | 7.28 |
| Phenols | BPS | 1223 | 92.80% | <LOD | 0.10 | 0.20 | 0.50 | 1.30 | 3.00 | 6.29 | 1446 | 89.07% | <LOD | <LOD | 0.20 | 0.50 | 1.20 | 2.75 | 4.70 |
| Phenols | TCC | 1224 | 38.15% | <LOD | <LOD | <LOD | <LOD | 0.20 | 2.84 | 19.09 | 1448 | 37.29% | <LOD | <LOD | <LOD | <LOD | 0.20 | 2.23 | 11.17 |
| Phenols | TCS | 1224 | 71.81% | <LOD | <LOD | <LOD | 4.30 | 23.60 | 198.53 | 430.46 | 1448 | 71.20% | <LOD | <LOD | <LOD | 4.60 | 23.63 | 179.93 | 404.98 |

Table S5. Associations between chemicals and molecules in the gonadal steroidogenic metabolic pathway among male and female children

| **Endogenous chemicals** | **Exogenous chemicals** | **Classification** | **Male children** | | | | **Female children** | | | |
| --- | --- | --- | --- | --- | --- | --- | --- | --- | --- | --- |
|  |  |  | ***P*** | ***β* (95%CI)** | **Number** | **VIP** | ***P*** | ***β* (95%CI)** | **Number** | **VIP** |
| E2 | MBP | PAEs | 0.53 | 0.01 (-0.03, 0.06) | 329 | 0.57 | 0.63 | -0.03 (-0.16, 0.09) | 327 | 1.01 |
|  | MBzP | PAEs | 0.65 | 0.01 (-0.03, 0.04) | 329 | 0.56 | 0.06 | -0.10 (-0.20, 0.01) | 327 | >1.00 |
|  | MCNP | PAEs | 0.73 | 0.01 (-0.04, 0.05) | 329 | 0.87 | 0.69 | 0.03 (-0.11, 0.16) | 327 | 0.62 |
|  | MCOCH | PAEs | 0.63 | -0.02 (-0.09, 0.05) | 182 | 0.18 | 0.15 | -0.16 (-0.37, 0.06) | 158 | 0.48 |
|  | MCOP | PAEs | 0.25 | 0.02 (-0.01, 0.05) | 329 | 0.94 | 0.36 | 0.05 (-0.06, 0.16) | 327 | 0.65 |
|  | MCPP | PAEs | 0.97 | 0.001 (-0.04, 0.04) | 329 | 0.63 | 0.77 | -0.02 (-0.14, 0.10) | 327 | 0.69 |
|  | MECPP | PAEs | 0.68 | -0.01 (-0.06, 0.04) | 329 | 0.56 | 0.42 | -0.06 (-0.19, 0.08) | 327 | 0.91 |
|  | MEHHP | PAEs | 0.72 | -0.01 (-0.05, 0.04) | 329 | 0.63 | 0.21 | -0.08 (-0.21, 0.05) | 327 | 0.98 |
|  | MEHP | PAEs | 0.56 | -0.01 (-0.06, 0.03) | 329 | 0.90 | 0.24 | -0.09 (-0.23, 0.06) | 327 | 0.74 |
|  | MEOHP | PAEs | 0.84 | -0.004 (-0.05, 0.04) | 329 | 0.53 | 0.20 | -0.08 (-0.21, 0.04) | 327 | 1.03 |
|  | MEP | PAEs | 0.83 | 0.004 (-0.03, 0.04) | 329 | 0.51 | 0.51 | 0.03 (-0.06, 0.13) | 327 | 0.64 |
|  | MHBP | PAEs | 0.92 | 0.002 (-0.04, 0.05) | 329 | 0.48 | 0.17 | -0.09 (-0.23, 0.04) | 327 | 1.17 |
|  | MHiBP | PAEs | 0.51 | -0.01 (-0.06, 0.03) | 329 | 0.41 | 0.55 | -0.04 (-0.15, 0.08) | 327 | 0.90 |
|  | MHINCH | PAEs | 0.33 | -0.02 (-0.06, 0.02) | 329 | 0.23 | 0.02 | -0.15 (-0.27, -0.02) | 327 | 0.89 |
|  | MiBP | PAEs | 0.72 | -0.01 (-0.05, 0.03) | 329 | 0.43 | 0.84 | 0.01 (-0.10, 0.13) | 327 | 0.86 |
|  | MiNP | PAEs | 0.34 | 0.02 (-0.02, 0.07) | 329 | 0.89 | 0.16 | 0.10 (-0.04, 0.25) | 327 | 0.48 |
|  | BP | Parabens | 0.51 | -0.02 (-0.09, 0.04) | 329 | 0.56 | 0.28 | 0.08 (-0.06, 0.22) | 327 | 0.24 |
|  | EP | Parabens | 0.29 | -0.02 (-0.07, 0.02) | 329 | 0.61 | 0.28 | 0.06 (-0.05, 0.17) | 327 | 0.11 |
|  | MP | Parabens | 0.72 | -0.005 (-0.03, 0.02) | 329 | 0.35 | 0.03 | 0.08 (0.01, 0.15) | 327 | 0.45 |
|  | PP | Parabens | 0.32 | -0.01 (-0.04, 0.01) | 329 | 0.46 | 0.01 | 0.08 (0.02, 0.15) | 327 | 0.61 |
|  | MPAH | PFASs | 0.71 | -0.01 (-0.06, 0.04) | 184 | 0.13 | 0.96 | -0.01 (-0.22, 0.21) | 166 | 0.15 |
|  | n-PFOS | PFASs | 0.62 | -0.02 (-0.11, 0.06) | 184 | 0.23 | 0.17 | -0.25 (-0.59, 0.10) | 166 | 0.35 |
|  | PFBA | PFASs | 0.41 | 0.07 (-0.10, 0.24) | 147 | 0.14 | 0.76 | 0.06 (-0.31, 0.43) | 169 | 0.11 |
|  | PFDA | PFASs | 0.25 | -0.05 (-0.15, 0.04) | 184 | 0.52 | 0.71 | -0.05 (-0.35, 0.24) | 166 | 0.18 |
|  | PFHpA | PFASs | 0.17 | 0.13 (-0.05, 0.31) | 184 | 0.38 | 0.01 | -0.70 (-1.22, -0.19) | 166 | 0.68 |
|  | PFHxA | PFASs | 0.88 | -0.01 (-0.10, 0.08) | 147 | 0.27 | 0.55 | 0.09 (-0.20, 0.38) | 169 | 0.14 |
|  | PFHxS | PFASs | 0.50 | 0.02 (-0.04, 0.09) | 184 | 0.31 | 1.00 | -0.001 (-0.24, 0.24) | 166 | 0.10 |
|  | PFNA | PFASs | 0.30 | -0.04 (-0.11, 0.04) | 184 | 0.10 | 0.10 | -0.23 (-0.51, 0.04) | 166 | 0.46 |
|  | PFOA | PFASs | 0.31 | -0.06 (-0.17, 0.05) | 184 | 0.09 | 0.10 | -0.32 (-0.7, 0.06) | 166 | 0.58 |
|  | PFUA | PFASs | 0.87 | -0.01 (-0.12, 0.10) | 184 | 0.07 | 0.60 | 0.09 (-0.24, 0.42) | 166 | 0.18 |
|  | Sm-PFOS | PFASs | 0.95 | 0.002 (-0.07, 0.08) | 184 | 0.25 | 0.32 | -0.14 (-0.41, 0.13) | 166 | 0.42 |
|  | BP-3 | Phenols | 0.40 | -0.01 (-0.04, 0.01) | 329 | 0.64 | 0.31 | -0.04 (-0.11, 0.03) | 327 | 0.61 |
|  | BPA | Phenols | 0.27 | -0.02 (-0.07, 0.02) | 329 | 0.56 | 0.83 | -0.01 (-0.15, 0.12) | 327 | 0.77 |
|  | BPF | Phenols | 0.94 | 0.001 (-0.03, 0.04) | 329 | 0.42 | 0.40 | 0.05 (-0.06, 0.16) | 327 | 0.35 |
|  | BPS | Phenols | 0.10 | -0.03 (-0.07, 0.01) | 329 | 0.79 | 0.44 | 0.04 (-0.06, 0.15) | 327 | 0.53 |
|  | TCC | Phenols | 0.69 | -0.01 (-0.03, 0.02) | 329 | 0.24 | 0.66 | -0.03 (-0.14, 0.09) | 327 | 0.39 |
|  | TCS | Phenols | 0.68 | 0.01 (-0.02, 0.03) | 329 | 0.20 | 0.16 | 0.06 (-0.02, 0.14) | 327 | 0.48 |
| SHBG | MBP | PAEs | 0.20 | 0.04 (-0.02, 0.11) | 309 | 0.47 | 0.98 | -0.001 (-0.06, 0.06) | 307 | 0.46 |
|  | MBzP | PAEs | 0.56 | 0.01 (-0.04, 0.07) | 309 | 0.32 | 0.97 | 0.001 (-0.05, 0.05) | 307 | 0.43 |
|  | MCNP | PAEs | 0.82 | 0.01 (-0.06, 0.07) | 309 | 0.58 | 0.21 | -0.04 (-0.11, 0.03) | 307 | 0.42 |
|  | MCOCH | PAEs | 0.48 | 0.04 (-0.06, 0.13) | 173 | 0.14 | 0.93 | 0.004 (-0.09, 0.10) | 158 | 0.25 |
|  | MCOP | PAEs | 0.24 | -0.03 (-0.08, 0.02) | 309 | 0.94 | 0.47 | -0.02 (-0.08, 0.03) | 307 | 0.32 |
|  | MCPP | PAEs | 0.06 | 0.06 (-0.001, 0.12) | 309 | 0.89 | 0.91 | 0.004 (-0.06, 0.07) | 307 | 0.44 |
|  | MECPP | PAEs | 0.58 | -0.02 (-0.09, 0.05) | 309 | 0.77 | 0.77 | -0.01 (-0.08, 0.06) | 307 | 0.39 |
|  | MEHHP | PAEs | 0.55 | -0.02 (-0.09, 0.05) | 309 | 0.82 | 0.75 | -0.01 (-0.08, 0.06) | 307 | 0.45 |
|  | MEHP | PAEs | 0.96 | 0.002 (-0.07, 0.07) | 309 | 0.69 | 0.91 | -0.004 (-0.08, 0.07) | 307 | 0.42 |
|  | MEOHP | PAEs | 0.93 | 0.003 (-0.06, 0.07) | 309 | 0.69 | 0.81 | 0.01 (-0.06, 0.07) | 307 | 0.52 |
|  | MEP | PAEs | 0.40 | -0.02 (-0.08, 0.03) | 309 | 0.54 | 0.44 | -0.02 (-0.07, 0.03) | 307 | 0.14 |
|  | MHBP | PAEs | 0.07 | 0.06 (-0.005, 0.13) | 309 | 0.40 | 0.13 | 0.05 (-0.02, 0.12) | 307 | 0.69 |
|  | MHiBP | PAEs | 0.72 | 0.01 (-0.05, 0.08) | 309 | 0.44 | 0.93 | -0.003 (-0.06, 0.06) | 307 | 0.33 |
|  | MHINCH | PAEs | 0.05 | 0.06 (-0.001, 0.13) | 309 | 0.93 | 0.23 | 0.04 (-0.02, 0.10) | 307 | 0.78 |
|  | MiBP | PAEs | 0.87 | 0.01 (-0.06, 0.07) | 309 | 0.52 | 0.08 | -0.05 (-0.11, 0.01) | 307 | 0.72 |
|  | MiNP | PAEs | 0.62 | -0.02 (-0.09, 0.05) | 309 | 0.63 | 0.44 | -0.03 (-0.11, 0.05) | 307 | 0.38 |
|  | BP | Parabens | 0.93 | -0.004 (-0.10, 0.09) | 309 | 0.28 | 0.51 | -0.02 (-0.10, 0.05) | 307 | 0.19 |
|  | EP | Parabens | 0.78 | 0.01 (-0.05, 0.07) | 309 | 0.10 | 0.19 | -0.04 (-0.09, 0.02) | 307 | 0.71 |
|  | MP | Parabens | 0.72 | -0.01 (-0.05, 0.03) | 309 | 0.33 | 0.93 | -0.002 (-0.04, 0.04) | 307 | 0.46 |
|  | PP | Parabens | 0.70 | -0.01 (-0.04, 0.03) | 309 | 0.39 | 0.39 | 0.02 (-0.02, 0.05) | 307 | 0.68 |
|  | MPAH | PFASs | 0.11 | 0.06 (-0.02, 0.14) | 164 | 0.41 | 0.58 | 0.04 (-0.10, 0.18) | 137 | 0.27 |
|  | n-PFOS | PFASs | 0.13 | 0.10 (-0.03, 0.23) | 164 | 0.27 | 0.12 | 0.17 (-0.04, 0.39) | 137 | 0.32 |
|  | PFBA | PFASs | 0.51 | -0.10 (-0.39, 0.20) | 136 | 0.11 | 0.32 | 0.13 (-0.13, 0.39) | 149 | 0.19 |
|  | PFDA | PFASs | 0.10 | 0.12 (-0.02, 0.25) | 164 | 0.33 | 0.09 | 0.15 (-0.02, 0.33) | 137 | 0.26 |
|  | PFHpA | PFASs | 0.66 | 0.06 (-0.20, 0.32) | 164 | 0.28 | 0.16 | 0.24 (-0.09, 0.56) | 137 | 0.37 |
|  | PFHxA | PFASs | 0.26 | 0.09 (-0.07, 0.25) | 136 | 0.64 | 0.99 | -0.001 (-0.19, 0.18) | 149 | 0.04 |
|  | PFHxS | PFASs | 0.44 | 0.04 (-0.06, 0.15) | 164 | 0.04 | 0.65 | 0.04 (-0.12, 0.20) | 137 | 0.26 |
|  | PFNA | PFASs | 0.94 | 0.01 (-0.12, 0.13) | 164 | 0.10 | 0.18 | 0.12 (-0.06, 0.30) | 137 | 0.26 |
|  | PFOA | PFASs | 0.06 | 0.16 (-0.01, 0.33) | 164 | 0.13 | 0.04 | 0.25 (0.01, 0.49) | 137 | 0.62 |
|  | PFUA | PFASs | 0.36 | 0.07 (-0.08, 0.22) | 164 | 0.43 | 0.33 | 0.10 (-0.10, 0.30) | 137 | 0.40 |
|  | Sm-PFOS | PFASs | 0.21 | 0.07 (-0.04, 0.19) | 164 | 0.09 | 0.74 | 0.03 (-0.15, 0.21) | 137 | 0.32 |
|  | BP-3 | Phenols | 0.57 | -0.01 (-0.05, 0.03) | 309 | 0.49 | 0.81 | -0.005 (-0.04, 0.03) | 307 | 0.40 |
|  | BPA | Phenols | 0.86 | -0.01 (-0.07, 0.06) | 309 | 0.51 | 0.70 | 0.01 (-0.06, 0.08) | 307 | 0.54 |
|  | BPF | Phenols | 0.29 | 0.03 (-0.02, 0.08) | 309 | 0.51 | 0.38 | 0.03 (-0.03, 0.09) | 307 | 0.82 |
|  | BPS | Phenols | 0.43 | -0.02 (-0.08, 0.03) | 309 | 0.75 | 0.02 | -0.06 (-0.12, -0.01) | 307 | 0.94 |
|  | TCC | Phenols | 0.25 | -0.02 (-0.06, 0.02) | 309 | 0.18 | 0.65 | 0.01 (-0.05, 0.07) | 307 | 0.08 |
|  | TCS | Phenols | 0.25 | -0.02 (-0.06, 0.02) | 309 | 1.20 | 0.36 | -0.02 (-0.06, 0.02) | 307 | 0.31 |
| T | MBP | PAEs | 0.32 | 0.08 (-0.08, 0.23) | 347 | 0.17 | 0.75 | -0.01 (-0.10, 0.07) | 336 | 0.44 |
|  | MBzP | PAEs | 0.90 | 0.01 (-0.11, 0.12) | 347 | 0.23 | 0.53 | -0.02 (-0.10, 0.05) | 336 | 0.41 |
|  | MCNP | PAEs | 0.82 | -0.02 (-0.17, 0.13) | 347 | 0.23 | 0.43 | 0.04 (-0.06, 0.14) | 336 | 0.43 |
|  | MCOCH | PAEs | 0.32 | -0.12 (-0.36, 0.12) | 182 | 0.24 | 0.27 | -0.08 (-0.23, 0.07) | 161 | 0.35 |
|  | MCOP | PAEs | 0.73 | 0.02 (-0.10, 0.14) | 347 | 0.19 | 0.50 | 0.03 (-0.05, 0.10) | 336 | 0.09 |
|  | MCPP | PAEs | 0.37 | -0.06 (-0.19, 0.07) | 347 | 0.45 | 0.86 | -0.01 (-0.09, 0.08) | 336 | 0.39 |
|  | MECPP | PAEs | 0.73 | -0.03 (-0.19, 0.13) | 347 | 0.45 | 0.33 | -0.05 (-0.14, 0.05) | 336 | 0.33 |
|  | MEHHP | PAEs | 0.99 | 0.001 (-0.15, 0.15) | 347 | 0.33 | 0.24 | -0.06 (-0.15, 0.04) | 336 | 0.40 |
|  | MEHP | PAEs | 0.52 | 0.05 (-0.11, 0.21) | 347 | 0.30 | 0.63 | -0.03 (-0.13, 0.08) | 336 | 0.28 |
|  | MEOHP | PAEs | 0.99 | -0.001 (-0.15, 0.15) | 347 | 0.38 | 0.18 | -0.06 (-0.15, 0.03) | 336 | 0.45 |
|  | MEP | PAEs | 0.07 | 0.12 (-0.01, 0.24) | 347 | 0.26 | 0.49 | 0.02 (-0.04, 0.09) | 336 | 0.30 |
|  | MHBP | PAEs | 0.39 | 0.07 (-0.09, 0.23) | 347 | 0.19 | 0.07 | -0.09 (-0.18, 0.01) | 336 | 0.77 |
|  | MHiBP | PAEs | 0.57 | 0.04 (-0.10, 0.19) | 347 | 0.24 | 0.63 | -0.02 (-0.11, 0.06) | 336 | 0.43 |
|  | MHINCH | PAEs | 0.21 | -0.09 (-0.24, 0.05) | 347 | 0.35 | 0.02 | -0.10 (-0.19, -0.01) | 336 | 0.85 |
|  | MiBP | PAEs | 0.23 | 0.09 (-0.05, 0.23) | 347 | 0.39 | 0.63 | 0.02 (-0.06, 0.10) | 336 | 0.44 |
|  | MiNP | PAEs | 0.51 | 0.05 (-0.11, 0.21) | 347 | 0.08 | 0.11 | 0.08 (-0.02, 0.19) | 336 | 0.17 |
|  | BP | Parabens | 0.64 | -0.05 (-0.27, 0.17) | 347 | 0.33 | 0.81 | -0.01 (-0.12, 0.09) | 336 | 0.31 |
|  | EP | Parabens | 0.86 | 0.01 (-0.13, 0.16) | 347 | 0.31 | 0.93 | 0.004 (-0.08, 0.08) | 336 | 0.25 |
|  | MP | Parabens | 0.67 | -0.02 (-0.11, 0.07) | 347 | 0.39 | 0.12 | 0.04 (-0.01, 0.10) | 336 | 0.53 |
|  | PP | Parabens | 0.98 | -0.001 (-0.08, 0.08) | 347 | 0.27 | 0.09 | 0.04 (-0.01, 0.09) | 336 | 0.52 |
|  | MPAH | PFASs | 0.20 | -0.12 (-0.30, 0.06) | 198 | 0.12 | 0.77 | 0.03 (-0.14, 0.20) | 171 | 0.26 |
|  | n-PFOS | PFASs | 0.35 | 0.14 (-0.16, 0.44) | 198 | 0.64 | 0.17 | -0.18 (-0.44, 0.08) | 171 | 0.30 |
|  | PFBA | PFASs | 0.79 | 0.08 (-0.51, 0.66) | 165 | 0.21 | 0.13 | 0.22 (-0.07, 0.50) | 175 | 0.59 |
|  | PFDA | PFASs | 0.31 | -0.16 (-0.47, 0.15) | 198 | 0.01 | 0.34 | -0.11 (-0.33, 0.11) | 171 | 0.27 |
|  | PFHpA | PFASs | 0.98 | -0.01 (-0.60, 0.58) | 198 | 0.40 | <0.01 | -0.59 (-0.99, -0.19) | 171 | 0.68 |
|  | PFHxA | PFASs | 0.68 | -0.07 (-0.38, 0.25) | 165 | 0.28 | 0.52 | 0.07 (-0.15, 0.29) | 175 | 0.08 |
|  | PFHxS | PFASs | 0.39 | 0.1 (-0.13, 0.34) | 198 | 0.31 | 0.89 | -0.01 (-0.20, 0.17) | 171 | 0.10 |
|  | PFNA | PFASs | 0.91 | -0.02 (-0.28, 0.25) | 198 | 0.17 | <0.05 | -0.21 (-0.42, -0.001) | 171 | 0.59 |
|  | PFOA | PFASs | 0.76 | -0.06 (-0.45, 0.33) | 198 | 0.13 | 0.01 | -0.38 (-0.67, -0.09) | 171 | 0.75 |
|  | PFUA | PFASs | 0.98 | 0.005 (-0.34, 0.35) | 198 | 0.02 | 0.32 | -0.12 (-0.37, 0.12) | 171 | 0.52 |
|  | Sm-PFOS | PFASs | 0.13 | 0.20 (-0.06, 0.46) | 198 | 0.56 | 0.21 | -0.13 (-0.34, 0.08) | 171 | 0.44 |
|  | BP-3 | Phenols | 0.29 | 0.05 (-0.04, 0.13) | 347 | 0.72 | 0.75 | -0.01 (-0.06, 0.04) | 336 | 0.19 |
|  | BPA | Phenols | 0.80 | -0.02 (-0.17, 0.13) | 347 | 0.08 | 0.87 | -0.01 (-0.11, 0.09) | 336 | 0.38 |
|  | BPF | Phenols | 0.94 | -0.004 (-0.12, 0.11) | 347 | 0.43 | 0.63 | 0.02 (-0.06, 0.10) | 336 | 0.20 |
|  | BPS | Phenols | 0.67 | -0.03 (-0.15, 0.10) | 347 | 0.63 | 0.52 | 0.03 (-0.05, 0.10) | 336 | 0.39 |
|  | TCC | Phenols | 0.28 | -0.05 (-0.15, 0.04) | 347 | 0.29 | 0.81 | -0.01 (-0.09, 0.07) | 336 | 0.34 |
|  | TCS | Phenols | 0.84 | 0.01 (-0.08, 0.10) | 347 | 0.31 | 0.29 | 0.03 (-0.03, 0.09) | 336 | 0.31 |

Table S6. Associations between chemicals and molecules in the gonadal steroidogenic metabolic pathway among male and female adolescents

| **Endogenous chemicals** | **Exogenous chemicals** | **Classification** | **Male adolescents** | | | | **Female adolescents** | | | |
| --- | --- | --- | --- | --- | --- | --- | --- | --- | --- | --- |
|  |  |  | ***P*** | ***β* (95%CI)** | **Number** | **VIP** | ***P*** | ***β* (95%CI)** | **Number** | **VIP** |
| E2 | MBP | PAEs | 0.07 | -0.07 (-0.15, 0.005) | 401 | 1.20 | 0.64 | -0.02 (-0.11, 0.07) | 337 | 1.24 |
|  | MBzP | PAEs | 0.02 | -0.07 (-0.14, -0.01) | 401 | 1.06 | 0.30 | 0.04 (-0.04, 0.11) | 337 | 1.98 |
|  | MCNP | PAEs | 0.68 | -0.02 (-0.09, 0.06) | 401 | 0.56 | 0.81 | 0.01 (-0.07, 0.09) | 337 | 1.03 |
|  | MCOCH | PAEs | 0.05 | -0.10 (-0.20, 0.002) | 200 | 0.33 | 0.15 | -0.13 (-0.32, 0.05) | 149 | 1.01 |
|  | MCOP | PAEs | 0.13 | 0.04 (-0.01, 0.09) | 401 | 0.63 | 0.11 | 0.05 (-0.01, 0.12) | 337 | 1.39 |
|  | MCPP | PAEs | 0.74 | 0.01 (-0.05, 0.07) | 401 | 0.65 | 0.60 | 0.02 (-0.06, 0.09) | 337 | 1.15 |
|  | MECPP | PAEs | 0.04 | -0.09 (-0.17, -0.002) | 401 | 1.17 | 0.09 | -0.08 (-0.16, 0.01) | 337 | 1.46 |
|  | MEHHP | PAEs | 0.05 | -0.08 (-0.16, 0.001) | 401 | 1.19 | 0.12 | -0.06 (-0.15, 0.02) | 337 | 1.36 |
|  | MEHP | PAEs | 0.23 | -0.05 (-0.14, 0.03) | 401 | 0.70 | 0.10 | -0.08 (-0.18, 0.02) | 337 | 1.32 |
|  | MEOHP | PAEs | 0.02 | -0.10 (-0.18, -0.02) | 401 | 1.28 | 0.16 | -0.06 (-0.15, 0.02) | 337 | 1.36 |
|  | MEP | PAEs | <0.01 | 0.08 (0.03, 0.14) | 401 | 1.01 | 0.69 | -0.01 (-0.08, 0.05) | 337 | 0.83 |
|  | MHBP | PAEs | 0.06 | -0.08 (-0.16, 0.002) | 401 | 1.17 | 0.52 | -0.03 (-0.14, 0.07) | 336 | 1.11 |
|  | MHiBP | PAEs | 0.01 | -0.10 (-0.17, -0.02) | 401 | 1.26 | 0.25 | -0.05 (-0.14, 0.04) | 336 | 1.23 |
|  | MHINCH | PAEs | 0.21 | -0.04 (-0.11, 0.02) | 401 | 0.50 | 0.19 | -0.07 (-0.16, 0.03) | 337 | 0.98 |
|  | MiBP | PAEs | 0.10 | -0.06 (-0.14, 0.01) | 401 | 1.17 | 0.16 | -0.06 (-0.15, 0.03) | 337 | 1.34 |
|  | MiNP | PAEs | 0.16 | 0.05 (-0.02, 0.13) | 401 | 0.49 | 0.13 | 0.07 (-0.02, 0.16) | 337 | 0.99 |
|  | BP | Parabens | 0.99 | 0.0009 (-0.11, 0.11) | 401 | 0.36 | 0.62 | 0.02 (-0.05, 0.08) | 337 | 0.93 |
|  | EP | Parabens | 0.10 | 0.06 (-0.01, 0.14) | 401 | 0.47 | 0.34 | 0.03 (-0.03, 0.09) | 337 | 0.60 |
|  | MP | Parabens | 0.18 | 0.03 (-0.01, 0.07) | 401 | 0.27 | 0.31 | 0.03 (-0.02, 0.08) | 337 | 0.62 |
|  | PP | Parabens | 0.65 | 0.01 (-0.03, 0.04) | 401 | 0.04 | 0.39 | 0.02 (-0.03, 0.07) | 337 | 0.56 |
|  | MPAH | PFASs | 0.39 | -0.04 (-0.14, 0.05) | 396 | 0.84 | 0.25 | -0.08 (-0.22, 0.06) | 334 | 0.36 |
|  | n-PFOS | PFASs | 0.07 | 0.10 (-0.01, 0.22) | 396 | 0.57 | 0.54 | 0.05 (-0.11, 0.21) | 333 | 0.38 |
|  | PFBA | PFASs | 0.02 | -0.26 (-0.48, -0.04) | 201 | 0.83 | 0.01 | -0.28 (-0.50, -0.06) | 187 | 0.60 |
|  | PFDA | PFASs | 0.10 | 0.09 (-0.02, 0.21) | 396 | 0.74 | 0.49 | 0.05 (-0.10, 0.21) | 334 | 0.21 |
|  | PFHpA | PFASs | 0.16 | 0.18 (-0.07, 0.43) | 202 | 0.16 | 0.52 | -0.12 (-0.48, 0.25) | 184 | 0.14 |
|  | PFHxA | PFASs | 0.69 | 0.03 (-0.13, 0.19) | 201 | 0.23 | 0.70 | 0.03 (-0.14, 0.21) | 187 | 0.36 |
|  | PFHxS | PFASs | 0.33 | 0.04 (-0.05, 0.13) | 396 | 0.27 | 0.65 | 0.03 (-0.10, 0.16) | 334 | 0.37 |
|  | PFNA | PFASs | 0.57 | 0.04 (-0.09, 0.16) | 396 | 0.39 | 0.27 | -0.09 (-0.25, 0.07) | 334 | 0.63 |
|  | PFOA | PFASs | 0.80 | 0.02 (-0.13, 0.17) | 396 | 0.45 | 0.49 | -0.07 (-0.26, 0.12) | 333 | 0.23 |
|  | PFUA | PFASs | 0.22 | 0.10 (-0.06, 0.26) | 396 | 0.53 | 0.82 | -0.02 (-0.22, 0.18) | 334 | 0.33 |
|  | Sm-PFOS | PFASs | 0.02 | 0.16 (0.03, 0.29) | 396 | 0.50 | 0.35 | -0.08 (-0.25, 0.09) | 333 | 0.63 |
|  | BP-3 | Phenols | 0.29 | -0.02 (-0.07, 0.02) | 401 | 0.82 | 0.49 | -0.02 (-0.08, 0.04) | 337 | 0.64 |
|  | BPA | Phenols | 0.99 | -0.0003 (-0.08, 0.07) | 401 | 0.69 | 0.97 | -0.002 (-0.10, 0.09) | 337 | 0.85 |
|  | BPF | Phenols | 0.52 | -0.01 (-0.06, 0.03) | 401 | 0.37 | 0.71 | -0.01 (-0.08, 0.06) | 336 | 0.53 |
|  | BPS | Phenols | 0.51 | 0.02 (-0.04, 0.07) | 401 | 0.44 | 0.68 | 0.02 (-0.06, 0.09) | 336 | 0.82 |
|  | TCC | Phenols | 0.07 | -0.04 (-0.09, 0.004) | 401 | 0.33 | 0.87 | -0.01 (-0.08, 0.06) | 337 | 0.22 |
|  | TCS | Phenols | 0.40 | -0.02 (-0.06, 0.02) | 401 | 0.97 | 0.07 | -0.05 (-0.10, 0.004) | 337 | 0.77 |
| SHBG | MBP | PAEs | 0.71 | 0.01 (-0.05, 0.07) | 375 | 0.92 | 0.07 | 0.05 (-0.005, 0.11) | 323 | 1.22 |
|  | MBzP | PAEs | 0.58 | 0.01 (-0.03, 0.06) | 375 | 0.38 | 0.05 | 0.05 (-0.001, 0.10) | 323 | 1.07 |
|  | MCNP | PAEs | 0.94 | 0.002 (-0.05, 0.06) | 375 | 0.49 | 0.43 | -0.02 (-0.08, 0.03) | 323 | 1.44 |
|  | MCOCH | PAEs | 0.35 | -0.04 (-0.12, 0.04) | 196 | 0.30 | 0.18 | -0.08 (-0.20, 0.04) | 149 | 0.81 |
|  | MCOP | PAEs | 0.07 | -0.04 (-0.07, 0.003) | 375 | 0.43 | 0.80 | 0.01 (-0.04, 0.05) | 323 | 1.00 |
|  | MCPP | PAEs | 0.80 | -0.01 (-0.05, 0.04) | 375 | 0.53 | 0.72 | 0.01 (-0.04, 0.06) | 323 | 1.14 |
|  | MECPP | PAEs | 0.26 | -0.04 (-0.10, 0.03) | 375 | 0.25 | 0.86 | 0.01 (-0.06, 0.07) | 323 | 1.33 |
|  | MEHHP | PAEs | 0.11 | -0.05 (-0.11, 0.01) | 375 | 0.58 | 0.91 | -0.003 (-0.06, 0.05) | 323 | 1.40 |
|  | MEHP | PAEs | 0.46 | -0.02 (-0.09, 0.04) | 375 | 0.25 | 0.74 | 0.01 (-0.06, 0.08) | 323 | 1.14 |
|  | MEOHP | PAEs | 0.47 | -0.02 (-0.08, 0.04) | 375 | 0.14 | 0.73 | 0.01 (-0.05, 0.07) | 323 | 1.38 |
|  | MEP | PAEs | <0.01 | -0.08 (-0.12, -0.05) | 375 | 1.95 | 0.49 | 0.02 (-0.03, 0.06) | 323 | 1.01 |
|  | MHBP | PAEs | <0.05 | 0.06 (0.0006, 0.12) | 375 | 1.60 | <0.01 | 0.12 (0.05, 0.19) | 322 | 1.45 |
|  | MHiBP | PAEs | 0.33 | 0.03 (-0.03, 0.09) | 375 | 0.79 | 0.07 | 0.06 (-0.01, 0.12) | 322 | 1.10 |
|  | MHINCH | PAEs | 0.68 | -0.01 (-0.06, 0.04) | 375 | 0.18 | 0.33 | -0.03 (-0.10, 0.03) | 323 | 0.91 |
|  | MiBP | PAEs | 0.49 | -0.02 (-0.08, 0.04) | 375 | 0.25 | 0.46 | 0.02 (-0.04, 0.08) | 323 | 1.37 |
|  | MiNP | PAEs | 0.54 | -0.02 (-0.07, 0.04) | 375 | 0.69 | 1.00 | 0.0001 (-0.06, 0.07) | 323 | 0.79 |
|  | BP | Parabens | 0.57 | 0.02 (-0.06, 0.11) | 375 | 1.14 | 0.02 | 0.06 (0.01, 0.10) | 323 | 0.64 |
|  | EP | Parabens | 0.76 | -0.01 (-0.06, 0.05) | 375 | 0.42 | 0.04 | 0.04 (0.001, 0.08) | 323 | 0.88 |
|  | MP | Parabens | 0.16 | -0.02 (-0.05, 0.01) | 375 | 1.28 | 0.07 | 0.03 (-0.003, 0.07) | 323 | 1.03 |
|  | PP | Parabens | 0.14 | -0.02 (-0.05, 0.01) | 375 | 1.17 | <0.01 | 0.05 (0.02, 0.08) | 323 | 1.19 |
|  | MPAH | PFASs | 0.72 | 0.01 (-0.06, 0.08) | 371 | 0.61 | 0.29 | 0.05 (-0.04, 0.15) | 320 | 0.90 |
|  | n-PFOS | PFASs | 0.17 | 0.06 (-0.03, 0.14) | 371 | 1.30 | 0.13 | 0.08 (-0.03, 0.19) | 319 | 0.96 |
|  | PFBA | PFASs | 0.45 | 0.07 (-0.12, 0.27) | 179 | 0.53 | 0.10 | 0.14 (-0.03, 0.32) | 173 | 0.09 |
|  | PFDA | PFASs | 0.51 | 0.03 (-0.06, 0.11) | 371 | 1.05 | 0.57 | 0.03 (-0.08, 0.14) | 320 | 0.60 |
|  | PFHpA | PFASs | 0.29 | -0.10 (-0.28, 0.08) | 181 | 0.58 | 0.97 | -0.01 (-0.29, 0.28) | 170 | 0.05 |
|  | PFHxA | PFASs | 0.38 | 0.05 (-0.06, 0.17) | 179 | 0.38 | 0.90 | -0.01 (-0.14, 0.12) | 173 | 0.08 |
|  | PFHxS | PFASs | 0.67 | -0.01 (-0.08, 0.05) | 371 | 0.52 | 0.60 | -0.02 (-0.12, 0.07) | 320 | 0.70 |
|  | PFNA | PFASs | 0.53 | 0.03 (-0.06, 0.12) | 371 | 0.91 | 0.69 | -0.02 (-0.13, 0.09) | 320 | 0.39 |
|  | PFOA | PFASs | 0.78 | 0.02 (-0.09, 0.12) | 371 | 1.13 | 0.14 | -0.10 (-0.23, 0.03) | 319 | 0.61 |
|  | PFUA | PFASs | 0.91 | 0.01 (-0.11, 0.12) | 371 | 1.13 | 0.67 | 0.03 (-0.11, 0.17) | 320 | 0.56 |
|  | Sm-PFOS | PFASs | 0.79 | 0.01 (-0.08, 0.11) | 371 | 0.77 | 0.35 | -0.06 (-0.17, 0.06) | 319 | 0.30 |
|  | BP-3 | Phenols | 0.23 | -0.02 (-0.05, 0.01) | 375 | 0.85 | 0.82 | 0.005 (-0.04, 0.05) | 323 | 0.55 |
|  | BPA | Phenols | 0.53 | -0.02 (-0.08, 0.04) | 375 | 0.45 | 0.14 | 0.05 (-0.02, 0.11) | 323 | 0.94 |
|  | BPF | Phenols | 0.27 | -0.02 (-0.05, 0.01) | 375 | 0.50 | 0.97 | 0.001 (-0.05, 0.05) | 322 | 0.66 |
|  | BPS | Phenols | 0.33 | -0.02 (-0.06, 0.02) | 375 | 0.40 | 0.96 | 0.001 (-0.05, 0.05) | 322 | 1.32 |
|  | TCC | Phenols | 0.59 | 0.01 (-0.03, 0.05) | 375 | 0.76 | 0.40 | 0.02 (-0.03, 0.07) | 323 | 0.16 |
|  | TCS | Phenols | 0.26 | -0.02 (-0.05, 0.01) | 375 | 0.79 | 0.54 | -0.01 (-0.05, 0.02) | 323 | 0.42 |
| T | MBP | PAEs | 0.01 | -0.12 (-0.21, -0.03) | 407 | 1.28 | 0.78 | 0.01 (-0.04, 0.05) | 340 | 0.55 |
|  | MBzP | PAEs | <0.01 | -0.12 (-0.20, -0.04) | 407 | 1.28 | 0.79 | -0.01 (-0.04, 0.03) | 340 | 0.31 |
|  | MCNP | PAEs | 0.46 | -0.03 (-0.13, 0.06) | 407 | 0.74 | 0.61 | -0.01 (-0.06, 0.03) | 340 | 0.87 |
|  | MCOCH | PAEs | 0.02 | -0.16 (-0.29, -0.02) | 200 | 0.62 | 0.77 | -0.01 (-0.11, 0.08) | 151 | 0.08 |
|  | MCOP | PAEs | 0.68 | 0.01 (-0.05, 0.08) | 407 | 0.58 | 0.82 | -0.004 (-0.04, 0.03) | 340 | 0.75 |
|  | MCPP | PAEs | 0.69 | -0.02 (-0.09, 0.06) | 407 | 0.75 | 0.51 | -0.01 (-0.05, 0.03) | 340 | 1.12 |
|  | MECPP | PAEs | 0.01 | -0.13 (-0.24, -0.03) | 407 | 1.31 | 0.07 | -0.04 (-0.09, 0.004) | 340 | 1.54 |
|  | MEHHP | PAEs | 0.01 | -0.14 (-0.23, -0.04) | 407 | 1.39 | 0.21 | -0.03 (-0.07, 0.02) | 340 | 0.90 |
|  | MEHP | PAEs | 0.20 | -0.07 (-0.17, 0.04) | 407 | 0.92 | 0.16 | -0.04 (-0.09, 0.02) | 340 | 0.92 |
|  | MEOHP | PAEs | <0.01 | -0.15 (-0.25, -0.05) | 407 | 1.43 | 0.23 | -0.03 (-0.07, 0.02) | 340 | 0.88 |
|  | MEP | PAEs | 0.52 | 0.02 (-0.05, 0.09) | 407 | 0.72 | 0.82 | -0.004 (-0.04, 0.03) | 340 | 0.31 |
|  | MHBP | PAEs | 0.07 | -0.10 (-0.20, 0.01) | 407 | 1.12 | 0.93 | -0.003 (-0.06, 0.05) | 339 | 0.47 |
|  | MHiBP | PAEs | 0.03 | -0.10 (-0.20, -0.01) | 407 | 1.26 | 0.81 | -0.01 (-0.05, 0.04) | 339 | 0.30 |
|  | MHINCH | PAEs | 0.10 | -0.07 (-0.15, 0.01) | 407 | 0.58 | 0.90 | -0.003 (-0.06, 0.05) | 340 | 0.11 |
|  | MiBP | PAEs | 0.02 | -0.11 (-0.21, -0.02) | 407 | 1.25 | 0.95 | -0.001 (-0.05, 0.05) | 340 | 0.53 |
|  | MiNP | PAEs | 0.38 | 0.04 (-0.05, 0.13) | 407 | 0.98 | 0.63 | 0.01 (-0.04, 0.06) | 340 | 0.69 |
|  | BP | Parabens | 0.76 | -0.02 (-0.16, 0.12) | 407 | 0.34 | 0.85 | -0.004 (-0.04, 0.03) | 340 | 0.71 |
|  | EP | Parabens | 0.15 | 0.07 (-0.03, 0.16) | 407 | 0.75 | 0.65 | 0.01 (-0.03, 0.04) | 340 | 0.37 |
|  | MP | Parabens | 0.48 | 0.02 (-0.03, 0.07) | 407 | 0.46 | 0.39 | 0.01 (-0.02, 0.04) | 340 | 1.47 |
|  | PP | Parabens | 0.69 | -0.01 (-0.05, 0.04) | 407 | 0.37 | 0.75 | 0.004 (-0.02, 0.03) | 340 | 0.76 |
|  | MPAH | PFASs | 0.95 | -0.004 (-0.12, 0.11) | 402 | 0.52 | 0.74 | -0.01 (-0.09, 0.06) | 337 | 0.84 |
|  | n-PFOS | PFASs | 0.03 | 0.15 (0.02, 0.29) | 402 | 0.69 | 0.61 | -0.02 (-0.11, 0.06) | 336 | 0.99 |
|  | PFBA | PFASs | 0.29 | -0.15 (-0.42, 0.12) | 207 | 0.49 | 0.74 | 0.02 (-0.11, 0.15) | 188 | 0.71 |
|  | PFDA | PFASs | 0.17 | 0.10 (-0.04, 0.23) | 402 | 0.81 | 0.98 | -0.001 (-0.09, 0.08) | 337 | 0.98 |
|  | PFHpA | PFASs | 0.27 | 0.17 (-0.13, 0.47) | 208 | 0.32 | 0.05 | -0.20 (-0.40, 0.004) | 185 | 0.89 |
|  | PFHxA | PFASs | 0.70 | -0.04 (-0.23, 0.15) | 207 | 0.17 | 0.34 | 0.05 (-0.05, 0.15) | 188 | 0.84 |
|  | PFHxS | PFASs | 0.33 | 0.05 (-0.05, 0.16) | 402 | 0.35 | 0.97 | 0.002 (-0.07, 0.07) | 337 | 0.50 |
|  | PFNA | PFASs | 0.31 | 0.08 (-0.07, 0.22) | 402 | 0.24 | 0.18 | -0.06 (-0.14, 0.03) | 337 | 0.37 |
|  | PFOA | PFASs | 0.38 | 0.08 (-0.10, 0.26) | 402 | 0.49 | 0.10 | -0.09 (-0.19, 0.02) | 336 | 0.31 |
|  | PFUA | PFASs | 0.41 | 0.08 (-0.11, 0.27) | 402 | 0.53 | 0.79 | 0.02 (-0.09, 0.12) | 337 | 0.76 |
|  | Sm-PFOS | PFASs | <0.01 | 0.27 (0.12, 0.43) | 402 | 0.53 | 0.18 | -0.06 (-0.16, 0.03) | 336 | 0.77 |
|  | BP-3 | Phenols | 0.56 | -0.02 (-0.07, 0.04) | 407 | 0.77 | 0.53 | -0.01 (-0.04, 0.02) | 340 | 1.01 |
|  | BPA | Phenols | 0.97 | 0.002 (-0.09, 0.10) | 407 | 0.74 | 0.83 | -0.01 (-0.06, 0.04) | 340 | 0.64 |
|  | BPF | Phenols | 0.40 | -0.02 (-0.08, 0.03) | 407 | 0.44 | 0.63 | -0.01 (-0.05, 0.03) | 339 | 0.55 |
|  | BPS | Phenols | 0.59 | 0.02 (-0.05, 0.09) | 407 | 0.87 | 0.16 | 0.03 (-0.01, 0.07) | 339 | 0.61 |
|  | TCC | Phenols | 0.01 | -0.08 (-0.13, -0.02) | 407 | 0.75 | 0.65 | 0.01 (-0.03, 0.05) | 340 | 0.78 |
|  | TCS | Phenols | 0.65 | -0.01 (-0.06, 0.04) | 407 | 0.18 | 0.21 | -0.02 (-0.05, 0.01) | 340 | 0.34 |

Table S7. Associations between chemicals and molecules in the gonadal steroidogenic metabolic pathway among male adults

| **Endogenous chemicals** | **Exogenous chemicals** | **Classification** | **Male adults** | | | |
| --- | --- | --- | --- | --- | --- | --- |
|  |  |  | ***P*** | ***β* (95%CI)** | **Number** | **VIP** |
| E2 | MBP | PAEs | 0.72 | 0.004 (-0.02, 0.02) | 1181 | 0.65 |
|  | MBzP | PAEs | 0.06 | 0.02 (-0.0004, 0.03) | 1181 | 1.05 |
|  | MCNP | PAEs | 0.80 | 0.003 (-0.02, 0.02) | 1181 | 0.41 |
|  | MCOCH | PAEs | 0.60 | 0.01 (-0.03, 0.05) | 587 | 0.51 |
|  | MCOP | PAEs | 0.76 | 0.002 (-0.01, 0.02) | 1181 | 0.51 |
|  | MCPP | PAEs | 0.75 | -0.003 (-0.02, 0.01) | 1181 | 0.19 |
|  | MECPP | PAEs | 0.22 | -0.01 (-0.04, 0.01) | 1181 | 0.42 |
|  | MEHHP | PAEs | 0.14 | -0.02 (-0.04, 0.01) | 1181 | 0.60 |
|  | MEHP | PAEs | 0.01 | -0.03 (-0.06, -0.01) | 1181 | 1.60 |
|  | MEOHP | PAEs | 0.12 | -0.02 (-0.04, 0.004) | 1181 | 0.65 |
|  | MEP | PAEs | 0.75 | 0.002 (-0.01, 0.02) | 1181 | 0.52 |
|  | MHBP | PAEs | 0.81 | -0.003 (-0.03, 0.02) | 1180 | 0.62 |
|  | MHiBP | PAEs | 0.46 | 0.01 (-0.01, 0.03) | 1180 | 0.96 |
|  | MHINCH | PAEs | 0.20 | 0.02 (-0.01, 0.04) | 1181 | 1.15 |
|  | MiBP | PAEs | 0.13 | 0.02 (-0.005, 0.04) | 1181 | 1.18 |
|  | MiNP | PAEs | 0.39 | -0.01 (-0.03, 0.01) | 1181 | 0.37 |
|  | BP | Parabens | 0.69 | 0.005 (-0.02, 0.03) | 1181 | 0.40 |
|  | EP | Parabens | 0.49 | -0.01 (-0.02, 0.01) | 1181 | 0.53 |
|  | MP | Parabens | 0.86 | -0.001 (-0.01, 0.01) | 1181 | 0.30 |
|  | PP | Parabens | 0.46 | -0.004 (-0.01, 0.01) | 1181 | 0.19 |
|  | MPAH | PFASs | 0.15 | 0.02 (-0.01, 0.05) | 1181 | 1.21 |
|  | n-PFOS | PFASs | 0.15 | 0.02 (-0.01, 0.04) | 1180 | 0.79 |
|  | PFBA | PFASs | 0.66 | -0.02 (-0.09, 0.06) | 593 | 0.49 |
|  | PFDA | PFASs | 0.54 | -0.01 (-0.03, 0.02) | 1181 | 0.77 |
|  | PFHpA | PFASs | 0.01 | 0.18 (0.04, 0.32) | 596 | 0.92 |
|  | PFHxA | PFASs | <0.01 | 0.07 (0.03, 0.12) | 593 | 1.13 |
|  | PFHxS | PFASs | 0.94 | 0.001 (-0.03, 0.03) | 1181 | 0.55 |
|  | PFNA | PFASs | 0.82 | 0.004 (-0.03, 0.04) | 1181 | 0.72 |
|  | PFOA | PFASs | 0.80 | -0.004 (-0.04, 0.03) | 1180 | 0.75 |
|  | PFUA | PFASs | 0.66 | 0.01 (-0.02, 0.03) | 1181 | 0.59 |
|  | Sm-PFOS | PFASs | 0.65 | 0.01 (-0.02, 0.03) | 1180 | 0.45 |
|  | BP-3 | Phenols | <0.01 | -0.02 (-0.03, -0.01) | 1181 | 2.20 |
|  | BPA | Phenols | 0.35 | -0.01 (-0.03, 0.01) | 1181 | 0.64 |
|  | BPF | Phenols | 0.59 | -0.004 (-0.02, 0.01) | 1180 | 0.66 |
|  | BPS | Phenols | 0.42 | -0.01 (-0.02, 0.01) | 1180 | 0.59 |
|  | TCC | Phenols | 0.02 | 0.01 (0.002, 0.03) | 1181 | 1.23 |
|  | TCS | Phenols | 0.38 | -0.01 (-0.02, 0.01) | 1181 | 1.12 |
| HDL-C | MBP | PAEs | 0.45 | -0.01 (-0.02, 0.01) | 1224 | 1.35 |
|  | MBzP | PAEs | 0.06 | -0.01 (-0.03, 0.001) | 1224 | 1.09 |
|  | MCNP | PAEs | 0.22 | -0.01 (-0.02, 0.01) | 1224 | 0.96 |
|  | MCOCH | PAEs | 0.90 | 0.002 (-0.03, 0.03) | 599 | 0.25 |
|  | MCOP | PAEs | 0.01 | -0.02 (-0.03, -0.004) | 1224 | 0.87 |
|  | MCPP | PAEs | 0.02 | -0.02 (-0.03, -0.002) | 1224 | 1.08 |
|  | MECPP | PAEs | <0.01 | -0.03 (-0.05, -0.02) | 1224 | 1.40 |
|  | MEHHP | PAEs | <0.01 | -0.03 (-0.04, -0.01) | 1224 | 1.30 |
|  | MEHP | PAEs | 0.10 | -0.02 (-0.03, 0.003) | 1224 | 1.05 |
|  | MEOHP | PAEs | <0.01 | -0.03 (-0.05, -0.01) | 1224 | 1.37 |
|  | MEP | PAEs | 0.53 | -0.003 (-0.01, 0.01) | 1224 | 0.69 |
|  | MHBP | PAEs | 0.07 | -0.02 (-0.03, 0.001) | 1223 | 0.96 |
|  | MHiBP | PAEs | 0.06 | -0.02 (-0.03, 0.001) | 1223 | 1.14 |
|  | MHINCH | PAEs | 0.46 | 0.01 (-0.01, 0.02) | 1224 | 0.49 |
|  | MiBP | PAEs | 0.25 | -0.01 (-0.03, 0.01) | 1224 | 1.15 |
|  | MiNP | PAEs | 0.24 | -0.01 (-0.03, 0.01) | 1224 | 0.71 |
|  | BP | Parabens | 0.15 | 0.01 (-0.005, 0.03) | 1224 | 0.51 |
|  | EP | Parabens | <0.01 | 0.04 (0.03, 0.05) | 1224 | 1.94 |
|  | MP | Parabens | 0.42 | 0.004 (-0.01, 0.01) | 1224 | 0.54 |
|  | PP | Parabens | 0.31 | 0.004 (-0.004, 0.01) | 1224 | 0.39 |
|  | MPAH | PFASs | 0.01 | 0.03 (0.01, 0.05) | 1221 | 0.55 |
|  | n-PFOS | PFASs | <0.01 | 0.04 (0.02, 0.06) | 1220 | 1.01 |
|  | PFBA | PFASs | 0.50 | 0.02 (-0.03, 0.07) | 624 | 0.13 |
|  | PFDA | PFASs | <0.01 | 0.03 (0.01, 0.05) | 1221 | 0.82 |
|  | PFHpA | PFASs | 0.01 | 0.13 (0.04, 0.22) | 625 | 0.40 |
|  | PFHxA | PFASs | 0.09 | 0.03 (-0.004, 0.06) | 624 | 0.23 |
|  | PFHxS | PFASs | <0.01 | 0.03 (0.01, 0.05) | 1221 | 1.01 |
|  | PFNA | PFASs | 0.02 | 0.03 (0.005, 0.06) | 1221 | 0.82 |
|  | PFOA | PFASs | 0.01 | 0.04 (0.01, 0.06) | 1220 | 0.88 |
|  | PFUA | PFASs | <0.01 | 0.05 (0.03, 0.07) | 1221 | 0.91 |
|  | Sm-PFOS | PFASs | <0.01 | 0.03 (0.01, 0.06) | 1220 | 0.92 |
|  | BP-3 | Phenols | 0.61 | 0.002 (-0.01, 0.01) | 1224 | 0.65 |
|  | BPA | Phenols | 0.01 | -0.02 (-0.04, -0.01) | 1224 | 0.94 |
|  | BPF | Phenols | 0.83 | 0.001 (-0.01, 0.01) | 1223 | 1.05 |
|  | BPS | Phenols | 0.08 | -0.01 (-0.02, 0.001) | 1223 | 0.60 |
|  | TCC | Phenols | 0.76 | 0.001 (-0.01, 0.01) | 1224 | 0.18 |
|  | TCS | Phenols | 0.99 | 0.00003 (-0.01, 0.01) | 1224 | 0.27 |
| LDL-C | MBP | PAEs | <0.01 | -0.04 (-0.06, -0.02) | 574 | 1.26 |
|  | MBzP | PAEs | <0.01 | -0.04 (-0.06, -0.02) | 574 | 1.29 |
|  | MCNP | PAEs | <0.01 | -0.04 (-0.06, -0.02) | 574 | 1.04 |
|  | MCOCH | PAEs | 0.41 | -0.02 (-0.07, 0.03) | 261 | 0.42 |
|  | MCOP | PAEs | 0.03 | -0.02 (-0.04, -0.002) | 574 | 1.02 |
|  | MCPP | PAEs | <0.01 | -0.04 (-0.06, -0.02) | 574 | 1.25 |
|  | MECPP | PAEs | 0.17 | -0.02 (-0.04, 0.01) | 574 | 1.77 |
|  | MEHHP | PAEs | 0.09 | -0.02 (-0.04, 0.003) | 574 | 1.68 |
|  | MEHP | PAEs | 0.02 | -0.03 (-0.06, -0.01) | 574 | 1.06 |
|  | MEOHP | PAEs | 0.06 | -0.02 (-0.05, 0.001) | 574 | 1.63 |
|  | MEP | PAEs | 0.29 | -0.01 (-0.02, 0.01) | 574 | 0.74 |
|  | MHBP | PAEs | 0.07 | -0.02 (-0.05, 0.002) | 573 | 1.04 |
|  | MHiBP | PAEs | 0.04 | -0.03 (-0.05, -0.002) | 573 | 1.33 |
|  | MHINCH | PAEs | 0.04 | -0.03 (-0.06, -0.001) | 574 | 1.01 |
|  | MiBP | PAEs | 0.04 | -0.03 (-0.05, -0.002) | 574 | 1.50 |
|  | MiNP | PAEs | <0.01 | -0.04 (-0.07, -0.02) | 574 | 1.08 |
|  | BP | Parabens | 0.75 | 0.004 (-0.02, 0.03) | 574 | 1.13 |
|  | EP | Parabens | 0.06 | -0.02 (-0.03, 0.0004) | 574 | 1.01 |
|  | MP | Parabens | 0.15 | -0.01 (-0.03, 0.004) | 574 | 0.50 |
|  | PP | Parabens | 0.40 | -0.01 (-0.02, 0.01) | 574 | 0.69 |
|  | MPAH | PFASs | 0.75 | -0.01 (-0.04, 0.03) | 570 | 0.42 |
|  | n-PFOS | PFASs | 0.10 | 0.03 (-0.005, 0.06) | 569 | 0.82 |
|  | PFBA | PFASs | 0.56 | -0.03 (-0.11, 0.06) | 312 | 0.49 |
|  | PFDA | PFASs | 0.04 | 0.03 (0.001, 0.06) | 570 | 0.74 |
|  | PFHpA | PFASs | 0.96 | 0.004 (-0.15, 0.15) | 309 | 0.35 |
|  | PFHxA | PFASs | 0.64 | -0.01 (-0.06, 0.04) | 312 | 0.09 |
|  | PFHxS | PFASs | 0.62 | 0.01 (-0.02, 0.04) | 570 | 0.60 |
|  | PFNA | PFASs | 0.09 | 0.03 (-0.005, 0.07) | 570 | >1.00 |
|  | PFOA | PFASs | 0.14 | 0.03 (-0.01, 0.06) | 569 | 1.05 |
|  | PFUA | PFASs | 0.16 | 0.02 (-0.01, 0.06) | 570 | 0.62 |
|  | Sm-PFOS | PFASs | 0.10 | 0.03 (-0.01, 0.06) | 569 | 1.02 |
|  | BP-3 | Phenols | 0.09 | -0.01 (-0.02, 0.002) | 574 | 0.40 |
|  | BPA | Phenols | <0.01 | -0.03 (-0.05, -0.01) | 574 | 1.01 |
|  | BPF | Phenols | 0.15 | -0.01 (-0.03, 0.005) | 573 | 0.50 |
|  | BPS | Phenols | 0.02 | -0.02 (-0.04, -0.004) | 573 | 0.83 |
|  | TCC | Phenols | 0.11 | -0.01 (-0.02, 0.002) | 574 | 0.54 |
|  | TCS | Phenols | 0.09 | -0.01 (-0.02, 0.002) | 574 | 1.02 |
| SHBG | MBP | PAEs | 0.51 | -0.01 (-0.04, 0.02) | 1087 | 1.05 |
|  | MBzP | PAEs | 0.09 | -0.02 (-0.05, 0.003) | 1087 | 0.90 |
|  | MCNP | PAEs | <0.01 | -0.05 (-0.08, -0.02) | 1087 | 1.22 |
|  | MCOCH | PAEs | <0.01 | -0.08 (-0.13, -0.03) | 565 | 0.41 |
|  | MCOP | PAEs | <0.01 | -0.05 (-0.07, -0.03) | 1087 | 1.36 |
|  | MCPP | PAEs | <0.01 | -0.04 (-0.06, -0.01) | 1087 | 1.21 |
|  | MECPP | PAEs | 0.01 | -0.04 (-0.07, -0.01) | 1087 | 1.21 |
|  | MEHHP | PAEs | 0.19 | -0.02 (-0.05, 0.01) | 1087 | 1.16 |
|  | MEHP | PAEs | 0.23 | -0.02 (-0.05, 0.01) | 1087 | 1.02 |
|  | MEOHP | PAEs | 0.08 | -0.03 (-0.06, 0.003) | 1087 | 1.20 |
|  | MEP | PAEs | 0.69 | 0.004 (-0.02, 0.02) | 1087 | 0.75 |
|  | MHBP | PAEs | 0.15 | 0.02 (-0.01, 0.06) | 1086 | 1.35 |
|  | MHiBP | PAEs | 0.39 | -0.01 (-0.04, 0.02) | 1086 | 1.08 |
|  | MHINCH | PAEs | <0.01 | -0.06 (-0.09, -0.03) | 1087 | 0.75 |
|  | MiBP | PAEs | 0.02 | -0.04 (-0.07, -0.01) | 1087 | 1.00 |
|  | MiNP | PAEs | <0.01 | -0.06 (-0.09, -0.02) | 1087 | 1.16 |
|  | BP | Parabens | 0.29 | 0.02 (-0.01, 0.05) | 1087 | 0.22 |
|  | EP | Parabens | 0.65 | 0.005 (-0.02, 0.03) | 1087 | 0.23 |
|  | MP | Parabens | 0.22 | 0.01 (-0.01, 0.03) | 1087 | 0.66 |
|  | PP | Parabens | 0.57 | 0.004 (-0.01, 0.02) | 1087 | 0.47 |
|  | MPAH | PFASs | <0.01 | 0.10 (0.06, 0.14) | 1085 | 0.92 |
|  | n-PFOS | PFASs | <0.01 | 0.08 (0.04, 0.12) | 1084 | 1.15 |
|  | PFBA | PFASs | 0.40 | 0.05 (-0.06, 0.16) | 521 | 0.21 |
|  | PFDA | PFASs | 0.69 | 0.01 (-0.03, 0.05) | 1085 | 0.76 |
|  | PFHpA | PFASs | 0.05 | 0.18 (-0.001, 0.37) | 522 | 0.37 |
|  | PFHxA | PFASs | 0.14 | 0.05 (-0.02, 0.11) | 521 | 0.04 |
|  | PFHxS | PFASs | <0.01 | 0.06 (0.02, 0.10) | 1085 | 0.69 |
|  | PFNA | PFASs | 0.06 | 0.05 (-0.002, 0.09) | 1085 | 0.69 |
|  | PFOA | PFASs | 0.37 | 0.02 (-0.03, 0.07) | 1084 | 0.57 |
|  | PFUA | PFASs | 0.02 | 0.05 (0.01, 0.09) | 1085 | 0.78 |
|  | Sm-PFOS | PFASs | <0.01 | 0.10 (0.06, 0.15) | 1084 | 1.18 |
|  | BP-3 | Phenols | <0.01 | -0.04 (-0.06, -0.03) | 1087 | 0.87 |
|  | BPA | Phenols | 0.83 | -0.003 (-0.03, 0.03) | 1087 | 0.96 |
|  | BPF | Phenols | 0.26 | 0.01 (-0.01, 0.03) | 1086 | 0.38 |
|  | BPS | Phenols | 0.20 | -0.02 (-0.04, 0.01) | 1086 | 0.48 |
|  | TCC | Phenols | 0.01 | 0.02 (0.01, 0.04) | 1087 | 0.66 |
|  | TCS | Phenols | 0.59 | -0.004 (-0.02, 0.01) | 1087 | 0.37 |
| TC | MBP | PAEs | 0.01 | -0.01 (-0.03, -0.004) | 1224 | 1.14 |
|  | MBzP | PAEs | <0.01 | -0.02 (-0.03, -0.01) | 1224 | 1.03 |
|  | MCNP | PAEs | 0.01 | -0.01 (-0.02, -0.004) | 1224 | 1.00 |
|  | MCOCH | PAEs | 0.47 | 0.01 (-0.01, 0.03) | 599 | 0.39 |
|  | MCOP | PAEs | <0.05 | -0.01 (-0.02, -0.00003) | 1224 | 0.92 |
|  | MCPP | PAEs | <0.01 | -0.01 (-0.02, -0.004) | 1224 | 0.99 |
|  | MECPP | PAEs | 0.09 | -0.01 (-0.02, 0.002) | 1224 | 1.19 |
|  | MEHHP | PAEs | 0.21 | -0.01 (-0.02, 0.004) | 1224 | 1.39 |
|  | MEHP | PAEs | 0.02 | -0.01 (-0.03, -0.002) | 1224 | 1.03 |
|  | MEOHP | PAEs | <0.05 | -0.01 (-0.02, -0.00003) | 1224 | 1.23 |
|  | MEP | PAEs | 0.22 | -0.005 (-0.01, 0.003) | 1224 | 0.82 |
|  | MHBP | PAEs | 0.02 | -0.01 (-0.03, -0.002) | 1223 | 0.91 |
|  | MHiBP | PAEs | <0.01 | -0.02 (-0.03, -0.01) | 1223 | 1.05 |
|  | MHINCH | PAEs | 0.45 | -0.005 (-0.02, 0.01) | 1224 | 0.56 |
|  | MiBP | PAEs | 0.02 | -0.01 (-0.02, -0.002) | 1224 | 1.11 |
|  | MiNP | PAEs | 0.06 | -0.01 (-0.02, 0.0004) | 1224 | 0.68 |
|  | BP | Parabens | 0.56 | 0.004 (-0.01, 0.02) | 1224 | 0.47 |
|  | EP | Parabens | 0.84 | 0.001 (-0.01, 0.01) | 1224 | 0.32 |
|  | MP | Parabens | 0.08 | -0.01 (-0.01, 0.001) | 1224 | 0.46 |
|  | PP | Parabens | 0.21 | -0.004 (-0.01, 0.002) | 1224 | 0.37 |
|  | MPAH | PFASs | 0.43 | 0.01 (-0.01, 0.02) | 1221 | 0.90 |
|  | n-PFOS | PFASs | <0.01 | 0.03 (0.02, 0.04) | 1220 | 1.51 |
|  | PFBA | PFASs | 0.52 | 0.01 (-0.03, 0.05) | 624 | 0.08 |
|  | PFDA | PFASs | 0.01 | 0.02 (0.01, 0.03) | 1221 | 0.96 |
|  | PFHpA | PFASs | 0.04 | 0.07 (0.002, 0.14) | 625 | 0.56 |
|  | PFHxA | PFASs | 0.66 | -0.01 (-0.03, 0.02) | 624 | 0.29 |
|  | PFHxS | PFASs | <0.01 | 0.02 (0.01, 0.04) | 1221 | 1.06 |
|  | PFNA | PFASs | <0.01 | 0.03 (0.01, 0.05) | 1221 | 1.07 |
|  | PFOA | PFASs | <0.01 | 0.04 (0.02, 0.05) | 1220 | 1.34 |
|  | PFUA | PFASs | <0.01 | 0.03 (0.01, 0.04) | 1221 | 0.88 |
|  | Sm-PFOS | PFASs | <0.01 | 0.03 (0.02, 0.05) | 1220 | 1.56 |
|  | BP-3 | Phenols | 0.82 | -0.001 (-0.01, 0.01) | 1224 | 0.51 |
|  | BPA | Phenols | <0.01 | -0.02 (-0.03, -0.01) | 1224 | 1.14 |
|  | BPF | Phenols | 0.02 | -0.01 (-0.02, -0.002) | 1223 | 0.51 |
|  | BPS | Phenols | 0.23 | -0.01 (-0.01, 0.003) | 1223 | 0.52 |
|  | TCC | Phenols | 0.13 | -0.01 (-0.01, 0.001) | 1224 | 0.15 |
|  | TCS | Phenols | 0.02 | -0.01 (-0.01, -0.001) | 1224 | 1.33 |
| TG | MBP | PAEs | 0.07 | -0.04 (-0.09, 0.004) | 585 | 1.01 |
|  | MBzP | PAEs | 0.48 | -0.01 (-0.05, 0.03) | 585 | 0.52 |
|  | MCNP | PAEs | 0.40 | -0.02 (-0.07, 0.03) | 585 | 0.51 |
|  | MCOCH | PAEs | 0.53 | -0.03 (-0.14, 0.07) | 266 | 0.29 |
|  | MCOP | PAEs | 0.85 | -0.003 (-0.04, 0.03) | 585 | 0.67 |
|  | MCPP | PAEs | 0.07 | -0.04 (-0.08, 0.002) | 585 | 0.98 |
|  | MECPP | PAEs | 0.42 | 0.02 (-0.03, 0.07) | 585 | 1.04 |
|  | MEHHP | PAEs | 0.91 | 0.003 (-0.05, 0.05) | 585 | 0.80 |
|  | MEHP | PAEs | 0.03 | -0.06 (-0.12, -0.01) | 585 | 0.89 |
|  | MEOHP | PAEs | 0.82 | -0.01 (-0.05, 0.04) | 585 | 0.76 |
|  | MEP | PAEs | 0.36 | -0.02 (-0.05, 0.02) | 585 | 0.82 |
|  | MHBP | PAEs | 0.07 | -0.05 (-0.11, 0.005) | 584 | 0.75 |
|  | MHiBP | PAEs | 0.02 | -0.06 (-0.11, -0.01) | 584 | 0.66 |
|  | MHINCH | PAEs | 0.23 | -0.03 (-0.09, 0.02) | 585 | 0.21 |
|  | MiBP | PAEs | 0.11 | -0.04 (-0.09, 0.01) | 585 | 0.69 |
|  | MiNP | PAEs | 0.22 | -0.03 (-0.09, 0.02) | 585 | 0.79 |
|  | BP | Parabens | 0.97 | -0.001 (-0.05, 0.05) | 585 | 0.62 |
|  | EP | Parabens | 0.38 | -0.02 (-0.05, 0.02) | 585 | 0.80 |
|  | MP | Parabens | 0.20 | -0.02 (-0.05, 0.01) | 585 | 0.68 |
|  | PP | Parabens | 0.03 | -0.03 (-0.05, -0.003) | 585 | 0.86 |
|  | MPAH | PFASs | 0.23 | -0.04 (-0.11, 0.03) | 581 | 0.75 |
|  | n-PFOS | PFASs | 0.25 | -0.04 (-0.1, 0.03) | 580 | 1.03 |
|  | PFBA | PFASs | 0.90 | -0.01 (-0.18, 0.16) | 318 | 0.08 |
|  | PFDA | PFASs | 0.27 | -0.04 (-0.1, 0.03) | 581 | 1.39 |
|  | PFHpA | PFASs | 0.66 | 0.06 (-0.23, 0.35) | 315 | 0.58 |
|  | PFHxA | PFASs | 0.56 | -0.03 (-0.12, 0.07) | 318 | 0.18 |
|  | PFHxS | PFASs | 0.56 | -0.02 (-0.08, 0.05) | 581 | 0.86 |
|  | PFNA | PFASs | 0.86 | 0.01 (-0.07, 0.08) | 581 | 1.01 |
|  | PFOA | PFASs | 0.80 | 0.01 (-0.06, 0.08) | 580 | 0.90 |
|  | PFUA | PFASs | 0.13 | -0.05 (-0.12, 0.02) | 581 | 1.47 |
|  | Sm-PFOS | PFASs | 0.94 | -0.002 (-0.07, 0.06) | 580 | 0.69 |
|  | BP-3 | Phenols | 0.67 | 0.01 (-0.02, 0.03) | 585 | 0.85 |
|  | BPA | Phenols | 0.84 | -0.005 (-0.05, 0.04) | 585 | 0.80 |
|  | BPF | Phenols | 0.10 | -0.03 (-0.07, 0.01) | 584 | 0.82 |
|  | BPS | Phenols | 0.81 | 0.005 (-0.03, 0.04) | 584 | 0.50 |
|  | TCC | Phenols | 0.23 | -0.02 (-0.05, 0.01) | 585 | 0.70 |
|  | TCS | Phenols | 0.38 | -0.01 (-0.04, 0.01) | 585 | 0.30 |
| T | MBP | PAEs | 0.02 | -0.03 (-0.06, -0.01) | 1200 | 1.33 |
|  | MBzP | PAEs | 0.10 | -0.02 (-0.04, 0.003) | 1200 | 1.30 |
|  | MCNP | PAEs | 0.03 | -0.03 (-0.05, -0.003) | 1200 | 1.13 |
|  | MCOCH | PAEs | 0.99 | 0.0003 (-0.05, 0.05) | 588 | 0.29 |
|  | MCOP | PAEs | 0.01 | -0.02 (-0.04, -0.01) | 1200 | 1.11 |
|  | MCPP | PAEs | 0.01 | -0.03 (-0.05, -0.01) | 1200 | 1.18 |
|  | MECPP | PAEs | <0.01 | -0.06 (-0.08, -0.03) | 1200 | 1.33 |
|  | MEHHP | PAEs | <0.01 | -0.05 (-0.08, -0.03) | 1200 | 1.35 |
|  | MEHP | PAEs | 0.02 | -0.04 (-0.07, -0.01) | 1200 | 1.08 |
|  | MEOHP | PAEs | <0.01 | -0.06 (-0.08, -0.03) | 1200 | 1.35 |
|  | MEP | PAEs | 0.01 | -0.02 (-0.04, -0.005) | 1200 | 0.76 |
|  | MHBP | PAEs | 0.51 | -0.01 (-0.04, 0.02) | 1199 | 1.53 |
|  | MHiBP | PAEs | 0.20 | -0.02 (-0.04, 0.01) | 1199 | 1.41 |
|  | MHINCH | PAEs | 0.90 | 0.002 (-0.03, 0.03) | 1200 | 0.48 |
|  | MiBP | PAEs | 0.01 | -0.03 (-0.06, -0.01) | 1200 | 1.19 |
|  | MiNP | PAEs | 0.01 | -0.04 (-0.06, -0.01) | 1200 | 0.85 |
|  | BP | Parabens | 0.11 | 0.02 (-0.005, 0.05) | 1200 | 0.33 |
|  | EP | Parabens | 0.40 | -0.01 (-0.03, 0.01) | 1200 | 0.32 |
|  | MP | Parabens | 0.24 | -0.01 (-0.03, 0.01) | 1200 | 0.75 |
|  | PP | Parabens | 0.31 | -0.01 (-0.02, 0.01) | 1200 | 0.46 |
|  | MPAH | PFASs | 0.70 | 0.01 (-0.03, 0.04) | 1200 | 0.15 |
|  | n-PFOS | PFASs | 0.12 | 0.03 (-0.01, 0.06) | 1199 | 0.20 |
|  | PFBA | PFASs | 0.90 | 0.01 (-0.08, 0.09) | 611 | 0.09 |
|  | PFDA | PFASs | 0.70 | 0.01 (-0.03, 0.04) | 1200 | 0.20 |
|  | PFHpA | PFASs | 0.98 | 0.002 (-0.16, 0.16) | 614 | 0.46 |
|  | PFHxA | PFASs | 0.12 | 0.04 (-0.01, 0.10) | 611 | 0.18 |
|  | PFHxS | PFASs | 0.22 | 0.02 (-0.01, 0.06) | 1200 | 0.10 |
|  | PFNA | PFASs | 0.90 | -0.003 (-0.04, 0.04) | 1200 | 0.20 |
|  | PFOA | PFASs | 0.94 | 0.002 (-0.04, 0.04) | 1199 | 0.11 |
|  | PFUA | PFASs | 0.13 | 0.03 (-0.01, 0.06) | 1200 | 0.45 |
|  | Sm-PFOS | PFASs | 0.77 | 0.01 (-0.03, 0.04) | 1199 | 0.36 |
|  | BP-3 | Phenols | <0.01 | -0.02 (-0.04, -0.01) | 1200 | 0.63 |
|  | BPA | Phenols | 0.02 | -0.03 (-0.05, -0.005) | 1200 | 0.97 |
|  | BPF | Phenols | 0.53 | -0.01 (-0.03, 0.01) | 1199 | 0.73 |
|  | BPS | Phenols | 0.10 | -0.02 (-0.04, 0.003) | 1199 | 0.73 |
|  | TCC | Phenols | 0.56 | 0.005 (-0.01, 0.02) | 1200 | 0.23 |
|  | TCS | Phenols | 0.13 | -0.01 (-0.03, 0.003) | 1200 | 0.58 |

Table S8. Associations between chemicals and molecules in the gonadal steroidogenic metabolic pathway among female adults

| **Endogenous chemicals** | **Exogenous chemicals** | **Classification** | **Premenopausal women** | | | | **Postmenopausal women** | | | |
| --- | --- | --- | --- | --- | --- | --- | --- | --- | --- | --- |
|  |  |  | ***P*** | ***β* (95%CI)** | **Number** | **VIP** | ***P*** | ***β* (95%CI)** | **Number** | **VIP** |
| E2 | MBP | PAEs | 0.42 | -0.03 (-0.10, 0.04) | 624 | 1.18 | 0.24 | 0.05 (-0.04, 0.14) | 533 | 1.26 |
|  | MBzP | PAEs | 0.99 | -0.0005 (-0.07, 0.06) | 624 | 1.31 | <0.01 | 0.12 (0.04, 0.20) | 533 | 1.07 |
|  | MCNP | PAEs | 0.81 | 0.01 (-0.07, 0.09) | 624 | 1.27 | 0.03 | 0.11 (0.01, 0.21) | 533 | 1.06 |
|  | MCOCH | PAEs | 0.71 | -0.03 (-0.21, 0.14) | 310 | 0.36 | 0.98 | 0.002 (-0.17, 0.17) | 270 | 0.26 |
|  | MCOP | PAEs | 0.43 | 0.03 (-0.04, 0.09) | 624 | 1.21 | 0.03 | 0.08 (0.01, 0.16) | 533 | 0.99 |
|  | MCPP | PAEs | 0.56 | 0.02 (-0.05, 0.09) | 624 | 1.27 | 0.09 | 0.07 (-0.01, 0.16) | 533 | 1.08 |
|  | MECPP | PAEs | 0.76 | -0.01 (-0.10, 0.08) | 624 | 1.34 | 0.61 | -0.03 (-0.13, 0.08) | 533 | 1.38 |
|  | MEHHP | PAEs | 0.95 | -0.003 (-0.09, 0.08) | 624 | 1.54 | 0.93 | 0.004 (-0.10, 0.10) | 533 | 1.31 |
|  | MEHP | PAEs | 0.36 | -0.04 (-0.13, 0.05) | 624 | 1.12 | 0.57 | 0.04 (-0.09, 0.16) | 533 | 1.30 |
|  | MEOHP | PAEs | 0.72 | -0.02 (-0.10, 0.07) | 624 | 1.37 | 0.52 | 0.03 (-0.07, 0.13) | 533 | 1.28 |
|  | MEP | PAEs | 0.65 | -0.01 (-0.07, 0.05) | 624 | 0.90 | 0.54 | 0.02 (-0.05, 0.09) | 533 | 0.65 |
|  | MHBP | PAEs | 0.61 | -0.02 (-0.12, 0.07) | 623 | 1.10 | 0.55 | 0.03 (-0.07, 0.14) | 533 | 1.37 |
|  | MHiBP | PAEs | 0.96 | 0.002 (-0.08, 0.08) | 623 | 1.10 | 0.11 | 0.08 (-0.02, 0.17) | 533 | 1.18 |
|  | MHINCH | PAEs | 0.57 | 0.03 (-0.06, 0.12) | 624 | 0.59 | 0.14 | 0.08 (-0.03, 0.19) | 533 | 0.56 |
|  | MiBP | PAEs | 0.94 | -0.003 (-0.08, 0.08) | 624 | 1.29 | 0.43 | 0.04 (-0.06, 0.13) | 533 | 1.24 |
|  | MiNP | PAEs | 0.83 | 0.01 (-0.08, 0.10) | 624 | 1.07 | 0.01 | 0.19 (0.06, 0.32) | 533 | 0.73 |
|  | BP | Parabens | 0.11 | -0.04 (-0.09, 0.01) | 625 | 1.89 | 0.36 | 0.03 (-0.03, 0.09) | 533 | 0.16 |
|  | EP | Parabens | 0.58 | 0.01 (-0.04, 0.06) | 625 | 0.74 | 0.28 | -0.03 (-0.09, 0.02) | 533 | 0.94 |
|  | MP | Parabens | 0.44 | -0.02 (-0.07, 0.03) | 625 | 0.65 | 0.56 | 0.02 (-0.04, 0.08) | 533 | 0.69 |
|  | PP | Parabens | 0.72 | 0.01 (-0.04, 0.05) | 625 | 1.84 | 0.05 | 0.05 (-0.00009, 0.10) | 533 | 0.48 |
|  | MPAH | PFASs | 0.36 | 0.06 (-0.07, 0.20) | 625 | 1.59 | 0.01 | -0.18 (-0.32, -0.04) | 532 | 0.51 |
|  | n-PFOS | PFASs | 0.33 | -0.05 (-0.15, 0.05) | 624 | 0.33 | 0.02 | -0.14 (-0.26, -0.02) | 532 | 0.68 |
|  | PFBA | PFASs | 0.46 | 0.11 (-0.19, 0.42) | 314 | 0.07 | 0.62 | -0.06 (-0.32, 0.19) | 263 | 0.28 |
|  | PFDA | PFASs | 0.23 | -0.07 (-0.17, 0.04) | 625 | 0.22 | 0.89 | -0.01 (-0.13, 0.11) | 532 | 0.59 |
|  | PFHpA | PFASs | 0.21 | -0.25 (-0.64, 0.14) | 313 | 0.10 | 0.65 | -0.11 (-0.57, 0.36) | 260 | 0.32 |
|  | PFHxA | PFASs | 0.28 | 0.09 (-0.07, 0.25) | 314 | 0.41 | 0.62 | -0.05 (-0.26, 0.15) | 263 | 0.62 |
|  | PFHxS | PFASs | 0.08 | 0.09 (-0.01, 0.20) | 625 | 0.26 | <0.01 | -0.21 (-0.34, -0.09) | 532 | 0.86 |
|  | PFNA | PFASs | 0.02 | -0.15 (-0.27, -0.02) | 625 | 0.90 | 0.02 | -0.18 (-0.32, -0.03) | 532 | 0.67 |
|  | PFOA | PFASs | 0.20 | -0.08 (-0.19, 0.04) | 624 | 0.73 | 0.02 | -0.16 (-0.30, -0.03) | 532 | 0.77 |
|  | PFUA | PFASs | 0.30 | -0.06 (-0.19, 0.06) | 625 | 0.28 | 0.57 | -0.04 (-0.18, 0.10) | 532 | 0.50 |
|  | Sm-PFOS | PFASs | 0.43 | -0.05 (-0.16, 0.07) | 624 | 0.31 | <0.01 | -0.24 (-0.36, -0.11) | 532 | 0.69 |
|  | BP-3 | Phenols | 0.12 | 0.03 (-0.01, 0.08) | 625 | 0.85 | 0.60 | 0.01 (-0.03, 0.06) | 533 | 0.23 |
|  | BPA | Phenols | 0.40 | 0.03 (-0.04, 0.11) | 625 | 1.39 | 0.05 | 0.10 (-0.001, 0.20) | 533 | 0.95 |
|  | BPF | Phenols | 0.60 | 0.02 (-0.05, 0.08) | 624 | 0.96 | 0.08 | 0.07 (-0.01, 0.15) | 533 | 0.66 |
|  | BPS | Phenols | 0.52 | -0.02 (-0.09, 0.05) | 624 | 0.89 | 0.17 | -0.05 (-0.13, 0.02) | 533 | 1.20 |
|  | TCC | Phenols | 0.60 | -0.02 (-0.07, 0.04) | 625 | 0.82 | 0.45 | 0.02 (-0.04, 0.08) | 533 | 0.37 |
|  | TCS | Phenols | 0.55 | -0.01 (-0.06, 0.03) | 625 | 1.24 | 0.14 | -0.04 (-0.10, 0.01) | 533 | 0.75 |
| HDL-C | MBP | PAEs | <0.01 | -0.03 (-0.05, -0.01) | 717 | 1.03 | <0.01 | -0.03 (-0.05, -0.01) | 582 | 1.23 |
|  | MBzP | PAEs | <0.01 | -0.03 (-0.05, -0.02) | 717 | 1.15 | <0.01 | -0.03 (-0.05, -0.01) | 582 | 1.16 |
|  | MCNP | PAEs | <0.05 | -0.02 (-0.03, -0.000009) | 717 | 0.88 | 0.24 | -0.01 (-0.04, 0.01) | 582 | 0.98 |
|  | MCOCH | PAEs | 0.82 | -0.004 (-0.04, 0.03) | 349 | 0.25 | 0.32 | -0.02 (-0.06, 0.02) | 286 | 0.46 |
|  | MCOP | PAEs | 0.11 | -0.01 (-0.03, 0.003) | 717 | 0.78 | <0.01 | -0.03 (-0.04, -0.01) | 582 | 0.94 |
|  | MCPP | PAEs | 0.11 | -0.01 (-0.03, 0.003) | 717 | 0.91 | 0.02 | -0.02 (-0.04, -0.005) | 582 | 1.01 |
|  | MECPP | PAEs | <0.01 | -0.04 (-0.06, -0.02) | 717 | 1.12 | <0.01 | -0.04 (-0.06, -0.02) | 582 | 1.19 |
|  | MEHHP | PAEs | <0.01 | -0.03 (-0.05, -0.01) | 717 | 1.11 | <0.01 | -0.04 (-0.06, -0.02) | 582 | 1.24 |
|  | MEHP | PAEs | 0.03 | -0.02 (-0.04, -0.003) | 717 | 0.82 | 0.58 | -0.01 (-0.04, 0.02) | 582 | 1.36 |
|  | MEOHP | PAEs | <0.01 | -0.03 (-0.05, -0.01) | 717 | 1.14 | <0.01 | -0.04 (-0.06, -0.02) | 582 | 1.26 |
|  | MEP | PAEs | 0.68 | -0.003 (-0.02, 0.01) | 717 | 1.09 | 0.05 | -0.01 (-0.03, 0.0001) | 582 | 0.67 |
|  | MHBP | PAEs | 0.01 | -0.03 (-0.05, -0.01) | 715 | 1.01 | 0.04 | -0.02 (-0.05, -0.001) | 582 | 1.10 |
|  | MHiBP | PAEs | <0.01 | -0.04 (-0.05, -0.02) | 715 | 1.04 | 0.01 | -0.03 (-0.05, -0.01) | 582 | 1.14 |
|  | MHINCH | PAEs | 0.57 | -0.01 (-0.03, 0.02) | 717 | 0.51 | 0.66 | -0.01 (-0.03, 0.02) | 582 | 0.91 |
|  | MiBP | PAEs | <0.01 | -0.03 (-0.05, -0.02) | 717 | 1.10 | 0.01 | -0.03 (-0.05, -0.01) | 582 | 1.18 |
|  | MiNP | PAEs | 0.57 | -0.01 (-0.03, 0.01) | 717 | 0.58 | 0.27 | -0.02 (-0.05, 0.01) | 582 | 0.49 |
|  | BP | Parabens | 0.08 | 0.01 (-0.001, 0.02) | 718 | 0.32 | 0.04 | 0.01 (0.001, 0.03) | 582 | 0.51 |
|  | EP | Parabens | <0.01 | 0.02 (0.01, 0.03) | 718 | 0.83 | <0.01 | 0.02 (0.01, 0.03) | 582 | 1.32 |
|  | MP | Parabens | 0.89 | 0.001 (-0.01, 0.01) | 718 | 0.63 | 0.25 | 0.01 (-0.01, 0.02) | 582 | 1.16 |
|  | PP | Parabens | 0.30 | 0.01 (-0.005, 0.02) | 718 | 0.52 | 0.84 | 0.001 (-0.01, 0.01) | 582 | 0.42 |
|  | MPAH | PFASs | 0.45 | 0.01 (-0.02, 0.04) | 714 | 0.22 | 0.19 | 0.02 (-0.01, 0.05) | 584 | 0.27 |
|  | n-PFOS | PFASs | <0.01 | 0.05 (0.03, 0.07) | 713 | 1.21 | <0.01 | 0.04 (0.01, 0.07) | 584 | 0.84 |
|  | PFBA | PFASs | 0.60 | -0.02 (-0.09, 0.05) | 367 | 0.26 | 0.04 | 0.06 (0.001, 0.11) | 296 | 0.43 |
|  | PFDA | PFASs | <0.01 | 0.07 (0.04, 0.09) | 714 | 1.42 | <0.01 | 0.05 (0.02, 0.07) | 584 | 1.18 |
|  | PFHpA | PFASs | 0.63 | 0.03 (-0.08, 0.13) | 364 | 0.14 | <0.05 | 0.10 (0.002, 0.20) | 295 | 0.45 |
|  | PFHxA | PFASs | 0.55 | -0.01 (-0.06, 0.03) | 367 | 0.08 | 0.18 | -0.03 (-0.08, 0.01) | 296 | 0.52 |
|  | PFHxS | PFASs | <0.01 | 0.05 (0.02, 0.07) | 714 | 1.16 | 0.11 | 0.02 (-0.01, 0.05) | 584 | 0.53 |
|  | PFNA | PFASs | <0.01 | 0.07 (0.04, 0.09) | 714 | 1.21 | 0.16 | 0.02 (-0.01, 0.06) | 584 | 0.84 |
|  | PFOA | PFASs | <0.01 | 0.07 (0.04, 0.10) | 713 | 1.32 | 0.01 | 0.04 (0.01, 0.07) | 584 | 0.86 |
|  | PFUA | PFASs | <0.01 | 0.10 (0.08, 0.13) | 714 | 1.82 | <0.01 | 0.08 (0.05, 0.11) | 584 | 1.81 |
|  | Sm-PFOS | PFASs | <0.01 | 0.04 (0.01, 0.06) | 713 | 0.81 | 0.06 | 0.03 (-0.001, 0.06) | 584 | 0.61 |
|  | BP-3 | Phenols | 0.04 | 0.01 (0.0003, 0.02) | 718 | 0.85 | 0.02 | 0.01 (0.002, 0.02) | 582 | 0.85 |
|  | BPA | Phenols | <0.01 | -0.03 (-0.05, -0.01) | 718 | 0.92 | 0.03 | -0.02 (-0.05, -0.002) | 582 | 0.99 |
|  | BPF | Phenols | 0.03 | -0.02 (-0.03, -0.001) | 716 | 0.73 | 0.01 | -0.02 (-0.04, -0.01) | 582 | 0.71 |
|  | BPS | Phenols | <0.01 | -0.04 (-0.06, -0.03) | 716 | 1.44 | <0.01 | -0.03 (-0.04, -0.01) | 582 | 0.92 |
|  | TCC | Phenols | 0.06 | -0.01 (-0.03, 0.0003) | 718 | 0.42 | 0.17 | -0.01 (-0.02, 0.004) | 582 | 0.38 |
|  | TCS | Phenols | 0.01 | 0.01 (0.004, 0.03) | 718 | 0.80 | 0.55 | 0.004 (-0.01, 0.02) | 582 | 0.44 |
| LDL-C | MBP | PAEs | 0.96 | 0.001 (-0.03, 0.03) | 316 | 1.49 | 0.40 | -0.01 (-0.05, 0.02) | 278 | 1.02 |
|  | MBzP | PAEs | 0.44 | 0.01 (-0.02, 0.03) | 316 | 1.28 | 0.07 | -0.03 (-0.05, 0.002) | 278 | 1.64 |
|  | MCNP | PAEs | 0.87 | 0.002 (-0.03, 0.03) | 316 | 0.88 | 0.93 | -0.002 (-0.04, 0.03) | 278 | 0.70 |
|  | MCOCH | PAEs | 0.58 | -0.02 (-0.07, 0.04) | 150 | 0.52 | 0.80 | 0.01 (-0.05, 0.06) | 139 | 0.14 |
|  | MCOP | PAEs | 0.32 | 0.01 (-0.01, 0.03) | 316 | 0.70 | 0.92 | -0.001 (-0.03, 0.02) | 278 | 0.55 |
|  | MCPP | PAEs | 0.71 | 0.01 (-0.02, 0.03) | 316 | 0.96 | 0.67 | -0.01 (-0.04, 0.02) | 278 | 0.72 |
|  | MECPP | PAEs | 0.40 | 0.02 (-0.02, 0.05) | 316 | 1.28 | 0.38 | -0.02 (-0.05, 0.02) | 278 | 1.01 |
|  | MEHHP | PAEs | 0.49 | 0.01 (-0.02, 0.04) | 316 | 1.29 | 0.35 | -0.02 (-0.05, 0.02) | 278 | 1.05 |
|  | MEHP | PAEs | 0.49 | 0.01 (-0.02, 0.05) | 316 | 1.00 | 0.91 | -0.003 (-0.05, 0.04) | 278 | 0.87 |
|  | MEOHP | PAEs | 0.45 | 0.01 (-0.02, 0.04) | 316 | 1.31 | 0.43 | -0.01 (-0.05, 0.02) | 278 | 1.08 |
|  | MEP | PAEs | 0.53 | 0.01 (-0.02, 0.03) | 316 | 0.90 | 0.78 | 0.003 (-0.02, 0.03) | 278 | 0.48 |
|  | MHBP | PAEs | 0.47 | -0.01 (-0.05, 0.02) | 315 | 1.12 | 0.12 | -0.03 (-0.07, 0.01) | 278 | 0.82 |
|  | MHiBP | PAEs | 0.98 | 0.0003 (-0.03, 0.03) | 315 | 1.08 | 0.99 | 0.0002 (-0.03, 0.03) | 278 | 1.26 |
|  | MHINCH | PAEs | 0.47 | -0.01 (-0.05, 0.02) | 316 | 0.93 | 0.81 | 0.004 (-0.03, 0.04) | 278 | 0.84 |
|  | MiBP | PAEs | 0.99 | -0.0002 (-0.03, 0.03) | 316 | 1.37 | 0.60 | 0.01 (-0.02, 0.04) | 278 | 1.99 |
|  | MiNP | PAEs | 0.53 | 0.01 (-0.02, 0.05) | 316 | 0.65 | 0.88 | 0.004 (-0.04, 0.05) | 278 | 0.85 |
|  | BP | Parabens | 0.76 | 0.003 (-0.02, 0.02) | 316 | 0.53 | 0.78 | 0.003 (-0.02, 0.02) | 278 | 0.18 |
|  | EP | Parabens | 0.61 | -0.005 (-0.02, 0.01) | 316 | 0.59 | 0.86 | 0.002 (-0.02, 0.02) | 278 | 0.96 |
|  | MP | Parabens | 0.80 | -0.002 (-0.02, 0.02) | 316 | 0.58 | 0.09 | -0.02 (-0.04, 0.003) | 278 | 0.51 |
|  | PP | Parabens | 0.33 | -0.01 (-0.02, 0.01) | 316 | 0.57 | 0.92 | 0.001 (-0.02, 0.02) | 278 | 0.66 |
|  | MPAH | PFASs | 0.92 | 0.003 (-0.05, 0.06) | 310 | 0.37 | 0.39 | 0.02 (-0.03, 0.07) | 278 | 0.57 |
|  | n-PFOS | PFASs | 0.40 | -0.02 (-0.05, 0.02) | 309 | 0.70 | 0.02 | 0.05 (0.01, 0.09) | 278 | 1.13 |
|  | PFBA | PFASs | 0.13 | 0.09 (-0.03, 0.20) | 165 | 0.85 | 0.56 | 0.03 (-0.07, 0.12) | 139 | 0.18 |
|  | PFDA | PFASs | 0.97 | -0.001 (-0.05, 0.04) | 310 | 0.72 | 0.11 | 0.03 (-0.01, 0.07) | 278 | 1.00 |
|  | PFHpA | PFASs | 0.63 | 0.04 (-0.13, 0.21) | 162 | 0.14 | 0.48 | 0.05 (-0.09, 0.20) | 137 | 0.92 |
|  | PFHxA | PFASs | 0.39 | 0.03 (-0.04, 0.10) | 165 | 0.84 | 0.13 | 0.05 (-0.02, 0.13) | 139 | 0.99 |
|  | PFHxS | PFASs | <0.01 | -0.06 (-0.10, -0.02) | 310 | 1.31 | 0.01 | 0.06 (0.01, 0.10) | 278 | 1.35 |
|  | PFNA | PFASs | 0.21 | -0.03 (-0.08, 0.02) | 310 | 0.90 | 0.04 | 0.06 (0.003, 0.11) | 278 | 1.26 |
|  | PFOA | PFASs | 0.13 | -0.04 (-0.08, 0.01) | 309 | >1.00 | <0.01 | 0.08 (0.03, 0.12) | 278 | 1.46 |
|  | PFUA | PFASs | 0.26 | -0.03 (-0.08, 0.02) | 310 | 0.83 | 0.26 | 0.03 (-0.02, 0.07) | 278 | 0.84 |
|  | Sm-PFOS | PFASs | 0.72 | -0.01 (-0.05, 0.04) | 309 | 0.70 | 0.01 | 0.07 (0.02, 0.11) | 278 | 1.32 |
|  | BP-3 | Phenols | 0.97 | -0.0003 (-0.02, 0.02) | 316 | 0.02 | 0.18 | 0.01 (-0.01, 0.03) | 278 | 0.28 |
|  | BPA | Phenols | 0.79 | 0.004 (-0.03, 0.04) | 316 | 0.93 | 0.87 | -0.003 (-0.04, 0.03) | 278 | 0.77 |
|  | BPF | Phenols | 0.29 | 0.01 (-0.01, 0.04) | 315 | 0.64 | 0.86 | 0.003 (-0.03, 0.03) | 278 | 1.11 |
|  | BPS | Phenols | 0.49 | 0.01 (-0.02, 0.03) | 315 | 1.02 | 0.69 | -0.01 (-0.03, 0.02) | 278 | 0.55 |
|  | TCC | Phenols | 0.90 | -0.001 (-0.02, 0.02) | 316 | 0.40 | 0.58 | -0.01 (-0.03, 0.01) | 278 | 0.65 |
|  | TCS | Phenols | 0.73 | -0.003 (-0.02, 0.01) | 316 | 0.37 | 0.17 | 0.01 (-0.01, 0.03) | 278 | 1.60 |
| SHBG | MBP | PAEs | 0.09 | -0.04 (-0.08, 0.01) | 600 | 0.84 | 0.13 | -0.03 (-0.07, 0.01) | 504 | 0.94 |
|  | MBzP | PAEs | 0.10 | -0.03 (-0.07, 0.01) | 600 | 1.08 | 0.48 | -0.01 (-0.05, 0.02) | 504 | 0.95 |
|  | MCNP | PAEs | 0.08 | -0.04 (-0.09, 0.005) | 600 | 1.03 | 0.04 | -0.05 (-0.09, -0.002) | 504 | 1.10 |
|  | MCOCH | PAEs | 0.54 | -0.03 (-0.12, 0.06) | 303 | 0.26 | 0.16 | -0.05 (-0.13, 0.02) | 262 | 0.87 |
|  | MCOP | PAEs | 0.04 | -0.04 (-0.08, -0.002) | 600 | 1.34 | <0.01 | -0.06 (-0.10, -0.03) | 504 | 1.45 |
|  | MCPP | PAEs | 0.04 | -0.04 (-0.09, -0.001) | 600 | 1.10 | 0.03 | -0.04 (-0.08, -0.004) | 504 | 0.95 |
|  | MECPP | PAEs | 0.37 | -0.02 (-0.08, 0.03) | 600 | 1.03 | 0.09 | -0.04 (-0.09, 0.01) | 504 | 1.07 |
|  | MEHHP | PAEs | 0.59 | -0.01 (-0.06, 0.04) | 600 | 1.02 | 0.05 | -0.04 (-0.09, 0.0004) | 504 | 1.05 |
|  | MEHP | PAEs | 0.67 | 0.01 (-0.04, 0.07) | 600 | 1.16 | 0.62 | 0.01 (-0.04, 0.07) | 504 | 1.42 |
|  | MEOHP | PAEs | 0.89 | 0.004 (-0.05, 0.05) | 600 | 1.17 | 0.07 | -0.04 (-0.08, 0.003) | 504 | 1.07 |
|  | MEP | PAEs | 0.87 | -0.003 (-0.04, 0.03) | 600 | 0.59 | 0.10 | -0.03 (-0.06, 0.005) | 504 | 0.67 |
|  | MHBP | PAEs | 0.79 | -0.01 (-0.06, 0.05) | 599 | 0.77 | 0.26 | 0.03 (-0.02, 0.08) | 504 | 1.50 |
|  | MHiBP | PAEs | 0.57 | -0.01 (-0.06, 0.03) | 599 | 0.95 | 0.25 | -0.03 (-0.07, 0.02) | 504 | 1.08 |
|  | MHINCH | PAEs | 0.11 | -0.04 (-0.10, 0.01) | 600 | 0.56 | 0.04 | -0.05 (-0.10, -0.002) | 504 | 1.37 |
|  | MiBP | PAEs | 0.06 | -0.05 (-0.09, 0.001) | 600 | 0.98 | 0.03 | -0.05 (-0.09, -0.005) | 504 | 1.04 |
|  | MiNP | PAEs | 0.25 | -0.03 (-0.08, 0.02) | 600 | 0.96 | 0.46 | -0.02 (-0.08, 0.04) | 504 | 0.50 |
|  | BP | Parabens | 0.91 | 0.002 (-0.03, 0.03) | 601 | 0.89 | 0.06 | 0.03 (-0.001, 0.05) | 504 | 0.32 |
|  | EP | Parabens | 0.07 | 0.03 (-0.002, 0.06) | 601 | 1.20 | 0.96 | -0.001 (-0.03, 0.02) | 504 | 0.18 |
|  | MP | Parabens | 0.61 | 0.01 (-0.02, 0.04) | 601 | 1.39 | 0.36 | 0.01 (-0.01, 0.04) | 504 | 0.96 |
|  | PP | Parabens | 0.51 | 0.01 (-0.02, 0.03) | 601 | 1.02 | 0.94 | 0.001 (-0.02, 0.02) | 504 | 0.51 |
|  | MPAH | PFASs | 0.07 | 0.07 (-0.01, 0.15) | 603 | 1.05 | 0.04 | 0.07 (0.004, 0.13) | 504 | 0.57 |
|  | n-PFOS | PFASs | 0.13 | -0.05 (-0.10, 0.01) | 602 | 0.63 | 0.39 | 0.02 (-0.03, 0.08) | 504 | 0.71 |
|  | PFBA | PFASs | 0.40 | 0.09 (-0.12, 0.29) | 297 | 0.02 | 0.38 | 0.05 (-0.07, 0.17) | 242 | 0.06 |
|  | PFDA | PFASs | 0.98 | -0.001 (-0.06, 0.06) | 603 | 0.83 | 0.09 | 0.05 (-0.01, 0.10) | 504 | 0.52 |
|  | PFHpA | PFASs | 0.17 | -0.24 (-0.58, 0.10) | 298 | 0.77 | 0.02 | 0.25 (0.03, 0.46) | 240 | 0.39 |
|  | PFHxA | PFASs | 0.67 | 0.03 (-0.09, 0.14) | 297 | 0.06 | 0.51 | 0.03 (-0.06, 0.12) | 242 | 0.51 |
|  | PFHxS | PFASs | 0.70 | -0.01 (-0.07, 0.05) | 603 | 0.37 | 0.25 | 0.03 (-0.02, 0.09) | 504 | 0.83 |
|  | PFNA | PFASs | 0.47 | -0.03 (-0.10, 0.05) | 603 | 0.64 | 0.48 | -0.02 (-0.09, 0.04) | 504 | 0.97 |
|  | PFOA | PFASs | 0.62 | -0.02 (-0.09, 0.05) | 602 | 0.45 | 0.39 | 0.03 (-0.03, 0.09) | 504 | 0.75 |
|  | PFUA | PFASs | 0.47 | 0.03 (-0.05, 0.10) | 603 | 1.11 | 0.02 | 0.07 (0.01, 0.13) | 504 | 0.77 |
|  | Sm-PFOS | PFASs | 0.03 | -0.08 (-0.14, -0.01) | 602 | 1.01 | 0.38 | 0.03 (-0.03, 0.08) | 504 | 0.81 |
|  | BP-3 | Phenols | 0.06 | 0.02 (-0.001, 0.05) | 601 | 1.50 | 0.72 | -0.004 (-0.02, 0.02) | 504 | 0.25 |
|  | BPA | Phenols | 0.21 | -0.03 (-0.08, 0.02) | 601 | 0.76 | 0.36 | -0.02 (-0.07, 0.02) | 504 | 0.87 |
|  | BPF | Phenols | 0.11 | -0.03 (-0.07, 0.01) | 600 | 0.80 | 0.44 | -0.01 (-0.05, 0.02) | 504 | 0.55 |
|  | BPS | Phenols | <0.01 | -0.06 (-0.10, -0.02) | 600 | 1.50 | 0.18 | -0.02 (-0.06, 0.01) | 504 | 0.85 |
|  | TCC | Phenols | 0.76 | 0.01 (-0.03, 0.04) | 601 | 0.40 | 0.59 | -0.01 (-0.03, 0.02) | 504 | 0.53 |
|  | TCS | Phenols | 0.93 | -0.001 (-0.03, 0.03) | 601 | 0.49 | 0.48 | -0.01 (-0.03, 0.02) | 504 | 0.15 |
| TC | MBP | PAEs | 0.24 | -0.01 (-0.02, 0.005) | 717 | 1.33 | <0.01 | -0.02 (-0.03, -0.01) | 582 | 1.31 |
|  | MBzP | PAEs | 0.07 | -0.01 (-0.02, 0.001) | 717 | 1.20 | <0.01 | -0.03 (-0.04, -0.02) | 582 | 1.81 |
|  | MCNP | PAEs | 0.08 | -0.01 (-0.02, 0.001) | 717 | >1.00 | 0.23 | -0.01 (-0.02, 0.01) | 582 | 1.16 |
|  | MCOCH | PAEs | 0.35 | -0.01 (-0.04, 0.01) | 349 | 0.32 | 0.42 | -0.01 (-0.03, 0.01) | 286 | 0.24 |
|  | MCOP | PAEs | 0.47 | -0.003 (-0.01, 0.01) | 717 | 0.91 | 0.04 | -0.01 (-0.02, -0.0004) | 582 | 0.92 |
|  | MCPP | PAEs | 0.53 | -0.003 (-0.01, 0.01) | 717 | 1.21 | 0.02 | -0.01 (-0.03, -0.002) | 582 | 1.08 |
|  | MECPP | PAEs | 0.15 | -0.01 (-0.02, 0.004) | 717 | 1.29 | <0.01 | -0.02 (-0.04, -0.01) | 582 | 1.24 |
|  | MEHHP | PAEs | 0.46 | -0.005 (-0.02, 0.01) | 717 | 1.44 | 0.01 | -0.02 (-0.03, -0.01) | 582 | 1.31 |
|  | MEHP | PAEs | 0.45 | -0.01 (-0.02, 0.01) | 717 | 0.95 | 0.06 | -0.02 (-0.03, 0.001) | 582 | 1.11 |
|  | MEOHP | PAEs | 0.53 | -0.004 (-0.02, 0.01) | 717 | 1.55 | <0.01 | -0.02 (-0.04, -0.01) | 582 | 1.31 |
|  | MEP | PAEs | 0.72 | -0.002 (-0.01, 0.01) | 717 | 0.79 | 0.04 | -0.01 (-0.02, -0.0004) | 582 | 0.87 |
|  | MHBP | PAEs | 0.02 | -0.02 (-0.03, -0.003) | 715 | 1.08 | <0.01 | -0.03 (-0.04, -0.01) | 582 | 1.20 |
|  | MHiBP | PAEs | 0.01 | -0.02 (-0.03, -0.01) | 715 | 1.23 | 0.01 | -0.02 (-0.03, -0.01) | 582 | 1.24 |
|  | MHINCH | PAEs | 0.09 | -0.01 (-0.03, 0.002) | 717 | 0.60 | 0.63 | -0.004 (-0.02, 0.01) | 582 | 0.68 |
|  | MiBP | PAEs | 0.02 | -0.01 (-0.03, -0.002) | 717 | 1.24 | 0.01 | -0.02 (-0.03, -0.004) | 582 | 1.27 |
|  | MiNP | PAEs | 0.86 | -0.001 (-0.01, 0.01) | 717 | 0.67 | 0.10 | -0.02 (-0.03, 0.003) | 582 | 0.92 |
|  | BP | Parabens | 0.15 | 0.01 (-0.002, 0.01) | 718 | 0.52 | 0.74 | 0.001 (-0.01, 0.01) | 582 | 0.60 |
|  | EP | Parabens | 0.92 | -0.0004 (-0.01, 0.01) | 718 | 0.25 | 0.30 | 0.004 (-0.004, 0.01) | 582 | 0.35 |
|  | MP | Parabens | 0.42 | -0.003 (-0.01, 0.005) | 718 | 0.76 | 0.08 | -0.01 (-0.02, 0.001) | 582 | 0.59 |
|  | PP | Parabens | 0.39 | -0.003 (-0.01, 0.004) | 718 | 0.52 | 0.53 | -0.002 (-0.01, 0.005) | 582 | 0.55 |
|  | MPAH | PFASs | 0.63 | -0.01 (-0.03, 0.02) | 714 | 0.41 | 0.01 | 0.03 (0.01, 0.05) | 584 | 1.17 |
|  | n-PFOS | PFASs | 0.43 | 0.01 (-0.01, 0.02) | 713 | 0.58 | <0.01 | 0.04 (0.02, 0.05) | 584 | 1.10 |
|  | PFBA | PFASs | 0.02 | 0.05 (0.01, 0.10) | 367 | 0.85 | 0.43 | 0.02 (-0.02, 0.05) | 296 | 0.31 |
|  | PFDA | PFASs | 0.02 | 0.02 (0.004, 0.04) | 714 | 0.69 | <0.01 | 0.03 (0.01, 0.05) | 584 | 0.68 |
|  | PFHpA | PFASs | 0.64 | 0.02 (-0.05, 0.09) | 364 | 0.13 | 0.08 | 0.06 (-0.01, 0.13) | 295 | 0.35 |
|  | PFHxA | PFASs | 0.30 | 0.01 (-0.01, 0.04) | 367 | 0.59 | 0.67 | -0.01 (-0.04, 0.02) | 296 | 0.52 |
|  | PFHxS | PFASs | 0.79 | 0.002 (-0.01, 0.02) | 714 | 0.52 | <0.01 | 0.04 (0.02, 0.05) | 584 | 1.30 |
|  | PFNA | PFASs | 0.60 | 0.01 (-0.01, 0.03) | 714 | 0.59 | <0.01 | 0.04 (0.02, 0.06) | 584 | 1.23 |
|  | PFOA | PFASs | 0.26 | 0.01 (-0.01, 0.03) | 713 | 0.51 | <0.01 | 0.05 (0.03, 0.07) | 584 | 1.17 |
|  | PFUA | PFASs | 0.03 | 0.02 (0.002, 0.04) | 714 | 0.80 | <0.01 | 0.04 (0.02, 0.06) | 584 | 0.93 |
|  | Sm-PFOS | PFASs | 0.19 | 0.01 (-0.01, 0.03) | 713 | 0.34 | <0.01 | 0.04 (0.02, 0.06) | 584 | 1.48 |
|  | BP-3 | Phenols | 0.12 | 0.01 (-0.001, 0.01) | 718 | 1.68 | 0.07 | 0.01 (-0.001, 0.01) | 582 | 1.10 |
|  | BPA | Phenols | 0.23 | -0.01 (-0.02, 0.005) | 718 | 1.03 | 0.17 | -0.01 (-0.02, 0.004) | 582 | 1.13 |
|  | BPF | Phenols | 0.19 | -0.01 (-0.02, 0.003) | 716 | 0.72 | 0.93 | 0.0004 (-0.01, 0.01) | 582 | 1.05 |
|  | BPS | Phenols | 0.35 | -0.01 (-0.02, 0.01) | 716 | 0.93 | 0.03 | -0.01 (-0.02, -0.001) | 582 | 0.73 |
|  | TCC | Phenols | 0.22 | -0.01 (-0.01, 0.003) | 718 | 0.61 | 0.19 | -0.01 (-0.01, 0.003) | 582 | 0.30 |
|  | TCS | Phenols | 0.17 | 0.01 (-0.002, 0.01) | 718 | 1.02 | 0.20 | 0.005 (-0.003, 0.01) | 582 | 0.70 |
| TG | MBP | PAEs | 0.55 | -0.02 (-0.07, 0.04) | 318 | 0.54 | 0.67 | -0.01 (-0.07, 0.05) | 279 | 1.19 |
|  | MBzP | PAEs | 0.49 | 0.02 (-0.03, 0.06) | 318 | 0.77 | 0.86 | 0.004 (-0.04, 0.05) | 279 | 0.86 |
|  | MCNP | PAEs | 0.10 | -0.05 (-0.10, 0.01) | 318 | 1.04 | 0.22 | -0.04 (-0.10, 0.02) | 279 | 1.03 |
|  | MCOCH | PAEs | 0.76 | -0.02 (-0.12, 0.08) | 151 | 0.37 | 0.97 | 0.002 (-0.09, 0.09) | 139 | 0.52 |
|  | MCOP | PAEs | 0.33 | -0.02 (-0.06, 0.02) | 318 | 0.76 | 0.34 | -0.02 (-0.07, 0.02) | 279 | 1.14 |
|  | MCPP | PAEs | 0.28 | -0.03 (-0.08, 0.02) | 318 | 0.83 | 0.33 | -0.03 (-0.08, 0.03) | 279 | 1.19 |
|  | MECPP | PAEs | 0.61 | -0.02 (-0.08, 0.05) | 318 | 0.68 | 0.46 | -0.02 (-0.09, 0.04) | 279 | 1.17 |
|  | MEHHP | PAEs | 0.89 | -0.004 (-0.07, 0.06) | 318 | 1.07 | 0.72 | -0.01 (-0.08, 0.05) | 279 | 1.30 |
|  | MEHP | PAEs | 0.22 | -0.04 (-0.12, 0.03) | 318 | 0.35 | 0.26 | -0.05 (-0.13, 0.03) | 279 | 1.14 |
|  | MEOHP | PAEs | 0.91 | 0.004 (-0.06, 0.06) | 318 | 1.26 | 0.25 | -0.04 (-0.10, 0.03) | 279 | 1.17 |
|  | MEP | PAEs | 0.09 | -0.04 (-0.08, 0.01) | 318 | 0.76 | 0.40 | -0.02 (-0.06, 0.02) | 279 | 0.82 |
|  | MHBP | PAEs | 0.17 | -0.05 (-0.11, 0.02) | 317 | 0.80 | 0.16 | -0.05 (-0.12, 0.02) | 279 | 0.97 |
|  | MHiBP | PAEs | 0.26 | -0.03 (-0.09, 0.02) | 317 | 0.53 | 0.59 | -0.02 (-0.08, 0.04) | 279 | 1.28 |
|  | MHINCH | PAEs | 0.06 | -0.06 (-0.12, 0.004) | 318 | 1.05 | 0.57 | -0.02 (-0.08, 0.05) | 279 | 0.63 |
|  | MiBP | PAEs | 0.20 | -0.04 (-0.09, 0.02) | 318 | 0.46 | 0.99 | 0.0004 (-0.06, 0.06) | 279 | 1.32 |
|  | MiNP | PAEs | 0.03 | -0.07 (-0.14, -0.01) | 318 | 1.06 | 0.13 | -0.06 (-0.15, 0.02) | 279 | 0.93 |
|  | BP | Parabens | 0.46 | -0.01 (-0.05, 0.02) | 318 | 0.81 | 0.52 | -0.01 (-0.05, 0.03) | 279 | 0.59 |
|  | EP | Parabens | 0.01 | -0.04 (-0.08, -0.01) | 318 | 0.80 | 0.13 | -0.03 (-0.06, 0.01) | 279 | 1.34 |
|  | MP | Parabens | 0.01 | -0.05 (-0.08, -0.01) | 318 | 0.98 | 0.09 | -0.03 (-0.07, 0.01) | 279 | 1.53 |
|  | PP | Parabens | <0.01 | -0.06 (-0.09, -0.03) | 318 | 1.41 | 0.48 | -0.01 (-0.04, 0.02) | 279 | 1.13 |
|  | MPAH | PFASs | 0.94 | 0.004 (-0.10, 0.11) | 312 | 0.26 | 0.34 | 0.04 (-0.04, 0.13) | 279 | 0.54 |
|  | n-PFOS | PFASs | <0.01 | -0.1 (-0.17, -0.03) | 311 | 1.38 | 0.77 | 0.01 (-0.06, 0.09) | 279 | 0.49 |
|  | PFBA | PFASs | 0.01 | 0.32 (0.09, 0.54) | 166 | 1.13 | 0.37 | -0.08 (-0.26, 0.10) | 140 | 0.33 |
|  | PFDA | PFASs | 0.01 | -0.11 (-0.20, -0.03) | 312 | 1.61 | 0.96 | 0.002 (-0.07, 0.07) | 279 | 0.29 |
|  | PFHpA | PFASs | 0.76 | 0.05 (-0.28, 0.38) | 163 | 0.51 | 0.62 | 0.07 (-0.20, 0.34) | 138 | 0.26 |
|  | PFHxA | PFASs | 0.50 | 0.04 (-0.09, 0.18) | 166 | 0.28 | 0.75 | -0.02 (-0.15, 0.11) | 140 | 0.57 |
|  | PFHxS | PFASs | 0.04 | -0.08 (-0.16, -0.01) | 312 | 0.83 | 0.64 | 0.02 (-0.06, 0.1) | 279 | 0.44 |
|  | PFNA | PFASs | <0.01 | -0.19 (-0.28, -0.1) | 312 | 1.77 | 0.73 | 0.02 (-0.08, 0.11) | 279 | 0.82 |
|  | PFOA | PFASs | 0.01 | -0.12 (-0.21, -0.04) | 311 | 1.23 | 0.92 | -0.005 (-0.09, 0.08) | 279 | 0.46 |
|  | PFUA | PFASs | <0.01 | -0.18 (-0.28, -0.09) | 312 | 2.46 | 0.88 | -0.01 (-0.09, 0.08) | 279 | 0.62 |
|  | Sm-PFOS | PFASs | 0.08 | -0.07 (-0.15, 0.01) | 311 | 1.21 | 0.29 | 0.04 (-0.04, 0.13) | 279 | 0.89 |
|  | BP-3 | Phenols | 0.52 | 0.01 (-0.02, 0.04) | 318 | 1.49 | 0.27 | -0.02 (-0.04, 0.01) | 279 | 0.54 |
|  | BPA | Phenols | 0.69 | 0.01 (-0.05, 0.08) | 318 | 1.15 | 0.58 | -0.02 (-0.08, 0.05) | 279 | 0.94 |
|  | BPF | Phenols | 0.85 | -0.005 (-0.05, 0.04) | 317 | 0.24 | 0.28 | -0.03 (-0.08, 0.02) | 279 | 0.44 |
|  | BPS | Phenols | 0.87 | 0.004 (-0.04, 0.05) | 317 | 0.61 | 0.90 | -0.003 (-0.05, 0.05) | 279 | 0.72 |
|  | TCC | Phenols | 0.76 | -0.01 (-0.05, 0.03) | 318 | 0.08 | 0.34 | 0.02 (-0.02, 0.05) | 279 | 0.94 |
|  | TCS | Phenols | 0.14 | -0.02 (-0.06, 0.01) | 318 | 1.14 | 0.94 | 0.001 (-0.03, 0.03) | 279 | 0.88 |
| T | MBP | PAEs | 0.28 | 0.02 (-0.02, 0.06) | 645 | 0.67 | 0.54 | 0.01 (-0.03, 0.06) | 545 | 0.68 |
|  | MBzP | PAEs | 0.08 | 0.03 (-0.003, 0.06) | 645 | 0.82 | 0.54 | 0.01 (-0.03, 0.05) | 545 | 0.71 |
|  | MCNP | PAEs | 0.22 | 0.02 (-0.01, 0.06) | 645 | 0.80 | 0.06 | 0.05 (-0.003, 0.09) | 545 | 1.03 |
|  | MCOCH | PAEs | 0.72 | -0.01 (-0.09, 0.06) | 315 | 0.47 | 0.90 | 0.005 (-0.07, 0.08) | 271 | 0.02 |
|  | MCOP | PAEs | 0.45 | 0.01 (-0.02, 0.04) | 645 | 0.75 | 0.84 | -0.004 (-0.04, 0.03) | 545 | 0.81 |
|  | MCPP | PAEs | 0.78 | 0.01 (-0.03, 0.04) | 645 | 0.80 | 0.56 | -0.01 (-0.05, 0.03) | 545 | 0.84 |
|  | MECPP | PAEs | 0.96 | 0.001 (-0.04, 0.05) | 645 | 0.65 | 0.77 | 0.01 (-0.04, 0.06) | 545 | 0.87 |
|  | MEHHP | PAEs | 0.92 | -0.002 (-0.04, 0.04) | 645 | 0.64 | 0.88 | 0.004 (-0.04, 0.05) | 545 | 0.87 |
|  | MEHP | PAEs | 0.93 | -0.002 (-0.05, 0.04) | 645 | 0.38 | 0.41 | -0.03 (-0.09, 0.04) | 545 | 1.22 |
|  | MEOHP | PAEs | 0.74 | 0.01 (-0.03, 0.05) | 645 | 0.72 | 0.78 | 0.01 (-0.04, 0.05) | 545 | 0.85 |
|  | MEP | PAEs | 0.17 | 0.02 (-0.01, 0.05) | 645 | 0.36 | 0.63 | 0.01 (-0.02, 0.04) | 545 | 0.83 |
|  | MHBP | PAEs | 0.84 | 0.005 (-0.04, 0.05) | 644 | 0.68 | 0.80 | -0.01 (-0.06, 0.04) | 545 | 0.66 |
|  | MHiBP | PAEs | 0.36 | 0.02 (-0.02, 0.06) | 644 | 0.56 | 0.45 | -0.02 (-0.06, 0.03) | 545 | 1.05 |
|  | MHINCH | PAEs | 0.48 | -0.02 (-0.06, 0.03) | 645 | 0.76 | 0.62 | 0.01 (-0.04, 0.06) | 545 | 0.33 |
|  | MiBP | PAEs | 0.25 | 0.02 (-0.02, 0.06) | 645 | 0.55 | 0.45 | -0.02 (-0.06, 0.03) | 545 | 1.44 |
|  | MiNP | PAEs | 0.96 | 0.001 (-0.04, 0.05) | 645 | 0.36 | 0.78 | -0.01 (-0.07, 0.05) | 545 | 0.86 |
|  | BP | Parabens | 0.18 | 0.02 (-0.01, 0.04) | 646 | 0.33 | 0.25 | 0.02 (-0.01, 0.05) | 545 | 1.33 |
|  | EP | Parabens | 0.08 | 0.02 (-0.003, 0.05) | 646 | 0.65 | 0.89 | -0.002 (-0.03, 0.03) | 545 | 0.24 |
|  | MP | Parabens | 0.55 | 0.01 (-0.02, 0.03) | 646 | 0.40 | 0.32 | 0.02 (-0.01, 0.04) | 545 | 0.30 |
|  | PP | Parabens | 0.54 | 0.01 (-0.02, 0.03) | 646 | 0.65 | 0.37 | 0.01 (-0.01, 0.03) | 545 | 0.17 |
|  | MPAH | PFASs | 0.31 | 0.04 (-0.03, 0.10) | 647 | 0.32 | 0.24 | -0.04 (-0.11, 0.03) | 545 | 1.02 |
|  | n-PFOS | PFASs | 0.98 | 0.001 (-0.05, 0.05) | 646 | 0.33 | 0.81 | -0.01 (-0.06, 0.05) | 545 | 0.23 |
|  | PFBA | PFASs | 0.84 | -0.02 (-0.19, 0.16) | 330 | 0.08 | 0.71 | 0.02 (-0.10, 0.15) | 274 | 0.61 |
|  | PFDA | PFASs | 0.17 | -0.04 (-0.09, 0.02) | 647 | 0.81 | 0.71 | -0.01 (-0.07, 0.05) | 545 | 0.28 |
|  | PFHpA | PFASs | 0.85 | -0.02 (-0.26, 0.22) | 330 | 0.25 | 0.40 | 0.10 (-0.14, 0.34) | 272 | 0.52 |
|  | PFHxA | PFASs | 0.96 | 0.002 (-0.09, 0.10) | 330 | 0.43 | 0.38 | 0.05 (-0.06, 0.15) | 274 | 0.84 |
|  | PFHxS | PFASs | <0.01 | 0.10 (0.05, 0.15) | 647 | 2.37 | 0.19 | 0.04 (-0.02, 0.10) | 545 | 0.98 |
|  | PFNA | PFASs | 0.53 | -0.02 (-0.08, 0.04) | 647 | 0.30 | 0.34 | -0.03 (-0.11, 0.04) | 545 | 0.63 |
|  | PFOA | PFASs | 0.03 | 0.07 (0.01, 0.13) | 646 | 1.31 | 0.67 | 0.01 (-0.05, 0.08) | 545 | 0.48 |
|  | PFUA | PFASs | 0.12 | -0.05 (-0.11, 0.01) | 647 | 1.04 | 0.75 | -0.01 (-0.08, 0.06) | 545 | 0.24 |
|  | Sm-PFOS | PFASs | 0.14 | 0.04 (-0.01, 0.10) | 646 | 1.12 | 0.85 | 0.01 (-0.05, 0.07) | 545 | 0.13 |
|  | BP-3 | Phenols | 0.22 | 0.01 (-0.01, 0.04) | 646 | 0.59 | 0.55 | -0.01 (-0.03, 0.02) | 545 | 0.27 |
|  | BPA | Phenols | 0.04 | 0.04 (0.001, 0.08) | 646 | 1.03 | 0.27 | 0.03 (-0.02, 0.08) | 545 | 1.24 |
|  | BPF | Phenols | 0.32 | 0.02 (-0.02, 0.05) | 645 | 0.57 | 0.85 | -0.004 (-0.04, 0.03) | 545 | 0.27 |
|  | BPS | Phenols | 0.95 | -0.001 (-0.04, 0.03) | 645 | 0.70 | 0.07 | 0.04 (-0.002, 0.07) | 545 | 0.05 |
|  | TCC | Phenols | 0.36 | 0.01 (-0.02, 0.04) | 646 | 0.11 | 0.07 | 0.03 (-0.002, 0.06) | 545 | 2.17 |
|  | TCS | Phenols | 0.08 | 0.02 (-0.002, 0.05) | 646 | 0.32 | 0.20 | -0.02 (-0.04, 0.01) | 545 | 1.52 |

Table S9. Associations between EDCs and gonadal hormone abnormalities

| **Gonadal hormone abnormality** | **Exogenous chemicals** | **Classification** | **Male adults** | | | |
| --- | --- | --- | --- | --- | --- | --- |
|  |  |  | ***P*** | **OR (95%CI)** | **Number** | **VIP** |
| Abnormally high E2 | MBP | PAEs | 0.68 | 0.93 (0.68, 1.29) | 1160 | 0.60 |
|  | MBzP | PAEs | 0.70 | 0.95 (0.72, 1.25) | 1160 | 0.41 |
|  | MCNP | PAEs | 0.76 | 0.95 (0.69, 1.31) | 1160 | 0.98 |
|  | MCOCH | PAEs | 0.71 | 1.11 (0.63, 1.97) | 581 | 0.32 |
|  | MCOP | PAEs | 0.17 | 0.84 (0.65, 1.08) | 1160 | 1.46 |
|  | MCPP | PAEs | 0.07 | 0.75 (0.54, 1.02) | 1160 | 1.69 |
|  | MECPP | PAEs | 0.42 | 0.87 (0.61, 1.23) | 1160 | 0.96 |
|  | MEHHP | PAEs | 0.84 | 0.97 (0.69, 1.36) | 1160 | 0.81 |
|  | MEHP | PAEs | 0.85 | 0.96 (0.65, 1.43) | 1160 | 0.69 |
|  | MEOHP | PAEs | 0.59 | 0.91 (0.66, 1.27) | 1160 | 0.83 |
|  | MEP | PAEs | 0.50 | 0.92 (0.73, 1.17) | 1160 | 0.57 |
|  | MHBP | PAEs | 0.55 | 0.89 (0.60, 1.31) | 1159 | 0.60 |
|  | MHiBP | PAEs | 0.94 | 1.01 (0.72, 1.42) | 1159 | 0.48 |
|  | MHINCH | PAEs | 0.92 | 1.02 (0.71, 1.47) | 1160 | 0.23 |
|  | MiBP | PAEs | 0.67 | 1.08 (0.77, 1.51) | 1160 | 0.73 |
|  | MiNP | PAEs | 0.23 | 0.75 (0.46, 1.21) | 1160 | 1.30 |
|  | BP | Parabens | 0.41 | 1.13 (0.85, 1.50) | 1160 | 0.71 |
|  | EP | Parabens | 0.36 | 1.11 (0.89, 1.38) | 1160 | 0.79 |
|  | MP | Parabens | 0.84 | 1.02 (0.83, 1.26) | 1160 | 0.18 |
|  | PP | Parabens | 0.36 | 1.08 (0.92, 1.27) | 1160 | 0.78 |
|  | MPAH | PFASs | 0.75 | 1.08 (0.68, 1.71) | 1158 | 0.27 |
|  | n-PFOS | PFASs | 0.81 | 1.06 (0.68, 1.64) | 1157 | 0.20 |
|  | PFBA | PFASs | 0.92 | 0.94 (0.28, 3.19) | 578 | 0.06 |
|  | PFDA | PFASs | 0.62 | 0.89 (0.56, 1.41) | 1158 | 0.41 |
|  | PFHpA | PFASs | 0.12 | 2.72 (0.77, 9.58) | 579 | 1.03 |
|  | PFHxA | PFASs | <0.01 | 2.05 (1.26, 3.32) | 578 | 1.98 |
|  | PFHxS | PFASs | 0.30 | 1.30 (0.80, 2.11) | 1158 | 0.91 |
|  | PFNA | PFASs | 0.23 | 0.72 (0.42, 1.23) | 1158 | 0.99 |
|  | PFOA | PFASs | 0.61 | 0.87 (0.52, 1.47) | 1157 | 0.43 |
|  | PFUA | PFASs | 0.90 | 0.97 (0.60, 1.58) | 1158 | 0.10 |
|  | Sm-PFOS | PFASs | 0.82 | 1.06 (0.65, 1.73) | 1157 | 0.27 |
|  | BP-3 | Phenols | <0.01 | 0.74 (0.60, 0.91) | 1160 | 2.39 |
|  | BPA | Phenols | 0.02 | 1.42 (1.05, 1.93) | 1160 | 2.08 |
|  | BPF | Phenols | 0.41 | 0.89 (0.67, 1.18) | 1159 | 0.70 |
|  | BPS | Phenols | 0.71 | 0.95 (0.72, 1.25) | 1159 | 0.37 |
|  | TCC | Phenols | 0.01 | 1.21 (1.05, 1.40) | 1160 | 2.25 |
|  | TCS | Phenols | 0.26 | 1.10 (0.93, 1.31) | 1160 | 1.03 |
| Abnormally high T | MBP | PAEs | 0.15 | 1.34 (0.90, 2.01) | 930 | 1.50 |
|  | MBzP | PAEs | 0.01 | 1.57 (1.11, 2.23) | 930 | 1.34 |
|  | MCNP | PAEs | 0.58 | 1.12 (0.75, 1.67) | 930 | 0.97 |
|  | MCOCH | PAEs | 0.07 | 1.64 (0.96, 2.79) | 457 | 0.68 |
|  | MCOP | PAEs | 0.56 | 0.91 (0.66, 1.25) | 930 | 1.31 |
|  | MCPP | PAEs | 0.54 | 0.89 (0.61, 1.29) | 930 | 1.62 |
|  | MECPP | PAEs | 0.07 | 1.53 (0.97, 2.40) | 930 | 1.32 |
|  | MEHHP | PAEs | 0.03 | 1.65 (1.06, 2.55) | 930 | 1.39 |
|  | MEHP | PAEs | 0.08 | 1.49 (0.95, 2.33) | 930 | 1.14 |
|  | MEOHP | PAEs | 0.10 | 1.45 (0.93, 2.26) | 930 | 1.47 |
|  | MEP | PAEs | 0.32 | 1.15 (0.87, 1.50) | 930 | 0.92 |
|  | MHBP | PAEs | 0.20 | 1.30 (0.87, 1.96) | 930 | 1.24 |
|  | MHiBP | PAEs | 0.35 | 1.23 (0.80, 1.88) | 930 | 1.45 |
|  | MHINCH | PAEs | 0.04 | 1.43 (1.01, 2.02) | 930 | 1.04 |
|  | MiBP | PAEs | 0.11 | 1.42 (0.93, 2.17) | 930 | 1.32 |
|  | MiNP | PAEs | 0.37 | 0.75 (0.4, 1.41) | 930 | 1.17 |
|  | BP | Parabens | 0.31 | 1.18 (0.86, 1.61) | 930 | 0.59 |
|  | EP | Parabens | 0.74 | 0.94 (0.67, 1.33) | 930 | 0.55 |
|  | MP | Parabens | 0.44 | 0.90 (0.68, 1.18) | 930 | 1.18 |
|  | PP | Parabens | 0.93 | 0.99 (0.80, 1.23) | 930 | 0.56 |
|  | MPAH | PFASs | 0.30 | 1.32 (0.78, 2.23) | 927 | 0.53 |
|  | n-PFOS | PFASs | 0.91 | 1.03 (0.58, 1.83) | 927 | 0.36 |
|  | PFBA | PFASs | 0.99 | NA | 473 | 0.44 |
|  | PFDA | PFASs | 0.51 | 0.82 (0.45, 1.50) | 927 | 0.32 |
|  | PFHpA | PFASs | 0.99 | NA | 472 | 0.35 |
|  | PFHxA | PFASs | 0.89 | 0.93 (0.36, 2.45) | 473 | 0.07 |
|  | PFHxS | PFASs | 0.55 | 0.84 (0.48, 1.48) | 927 | 0.29 |
|  | PFNA | PFASs | 0.38 | 0.73 (0.37, 1.45) | 927 | 0.44 |
|  | PFOA | PFASs | 0.39 | 0.76 (0.41, 1.42) | 927 | 0.44 |
|  | PFUA | PFASs | 0.93 | 1.03 (0.57, 1.87) | 927 | 0.43 |
|  | Sm-PFOS | PFASs | 0.93 | 0.97 (0.53, 1.80) | 927 | 0.21 |
|  | BP-3 | Phenols | 0.41 | 0.90 (0.71, 1.15) | 930 | 0.87 |
|  | BPA | Phenols | <0.01 | 1.78 (1.23, 2.58) | 930 | 1.50 |
|  | BPF | Phenols | 0.66 | 1.07 (0.79, 1.44) | 930 | 0.54 |
|  | BPS | Phenols | 0.57 | 1.10 (0.79, 1.55) | 930 | 0.78 |
|  | TCC | Phenols | 0.04 | 1.21 (1.01, 1.46) | 930 | 1.15 |
|  | TCS | Phenols | 0.61 | 0.94 (0.73, 1.20) | 930 | 0.62 |
| E2 deficiency | MBP | PAEs | 0.38 | 0.84 (0.58, 1.23) | 1150 | 1.43 |
|  | MBzP | PAEs | 0.12 | 0.77 (0.55, 1.07) | 1150 | 1.33 |
|  | MCNP | PAEs | 0.84 | 0.96 (0.65, 1.41) | 1150 | 1.03 |
|  | MCOCH | PAEs | 0.33 | 0.41 (0.07, 2.44) | 572 | 0.45 |
|  | MCOP | PAEs | 0.57 | 0.92 (0.68, 1.24) | 1150 | 0.95 |
|  | MCPP | PAEs | 0.31 | 0.83 (0.57, 1.19) | 1150 | 1.09 |
|  | MECPP | PAEs | 0.94 | 0.98 (0.64, 1.51) | 1150 | 1.42 |
|  | MEHHP | PAEs | 0.78 | 0.94 (0.63, 1.42) | 1150 | 1.33 |
|  | MEHP | PAEs | 0.94 | 0.98 (0.61, 1.57) | 1150 | 1.11 |
|  | MEOHP | PAEs | 0.75 | 0.94 (0.63, 1.40) | 1150 | 1.35 |
|  | MEP | PAEs | 0.84 | 1.03 (0.79, 1.35) | 1150 | 1.03 |
|  | MHBP | PAEs | 0.35 | 0.79 (0.48, 1.29) | 1149 | 1.22 |
|  | MHiBP | PAEs | 0.27 | 0.80 (0.53, 1.20) | 1149 | 1.35 |
|  | MHINCH | PAEs | 0.42 | 0.79 (0.45, 1.40) | 1150 | 0.73 |
|  | MiBP | PAEs | 0.09 | 0.71 (0.47, 1.06) | 1150 | 1.56 |
|  | MiNP | PAEs | 0.82 | 0.95 (0.59, 1.51) | 1150 | 0.78 |
|  | BP | Parabens | 0.94 | 1.02 (0.66, 1.57) | 1150 | 0.10 |
|  | EP | Parabens | 0.59 | 0.91 (0.65, 1.28) | 1150 | 0.45 |
|  | MP | Parabens | 0.83 | 1.03 (0.80, 1.32) | 1150 | 0.65 |
|  | PP | Parabens | 0.34 | 1.10 (0.90, 1.34) | 1150 | 1.18 |
|  | MPAH | PFASs | 0.44 | 1.21 (0.74, 1.97) | 1152 | 0.73 |
|  | n-PFOS | PFASs | 0.88 | 0.96 (0.59, 1.57) | 1151 | 0.13 |
|  | PFBA | PFASs | 0.99 | NA | 577 | 0.82 |
|  | PFDA | PFASs | 0.85 | 0.95 (0.57, 1.59) | 1152 | 0.27 |
|  | PFHpA | PFASs | 0.76 | 0.66 (0.05, 9.27) | 580 | 0.25 |
|  | PFHxA | PFASs | 0.41 | 0.65 (0.24, 1.80) | 577 | 0.60 |
|  | PFHxS | PFASs | 0.61 | 1.15 (0.67, 1.97) | 1152 | 0.43 |
|  | PFNA | PFASs | 0.74 | 0.90 (0.49, 1.67) | 1152 | 0.29 |
|  | PFOA | PFASs | 0.33 | 1.40 (0.72, 2.72) | 1151 | 0.96 |
|  | PFUA | PFASs | 0.11 | 0.52 (0.24, 1.15) | 1152 | 1.54 |
|  | Sm-PFOS | PFASs | 0.58 | 1.18 (0.66, 2.09) | 1151 | 0.52 |
|  | BP-3 | Phenols | 0.41 | 1.09 (0.88, 1.36) | 1150 | 1.13 |
|  | BPA | Phenols | 0.33 | 1.21 (0.83, 1.76) | 1150 | 1.66 |
|  | BPF | Phenols | 0.61 | 1.07 (0.81, 1.42) | 1149 | 0.66 |
|  | BPS | Phenols | 0.69 | 0.93 (0.67, 1.31) | 1149 | 0.74 |
|  | TCC | Phenols | 0.45 | 0.89 (0.65, 1.21) | 1150 | 0.63 |
|  | TCS | Phenols | 0.51 | 0.92 (0.72, 1.17) | 1150 | 0.56 |
| T deficiency | MBP | PAEs | 0.02 | 1.17 (1.03, 1.33) | 1181 | 1.38 |
|  | MBzP | PAEs | 0.12 | 1.09 (0.98, 1.21) | 1181 | 1.27 |
|  | MCNP | PAEs | 0.07 | 1.12 (0.99, 1.27) | 1181 | 1.16 |
|  | MCOCH | PAEs | 0.72 | 1.04 (0.83, 1.32) | 578 | 0.27 |
|  | MCOP | PAEs | 0.16 | 1.07 (0.97, 1.17) | 1181 | 1.20 |
|  | MCPP | PAEs | 0.02 | 1.13 (1.02, 1.26) | 1181 | 1.14 |
|  | MECPP | PAEs | <0.01 | 1.42 (1.23, 1.64) | 1181 | 1.41 |
|  | MEHHP | PAEs | <0.01 | 1.37 (1.20, 1.57) | 1181 | 1.39 |
|  | MEHP | PAEs | 0.01 | 1.21 (1.04, 1.39) | 1181 | 1.16 |
|  | MEOHP | PAEs | <0.01 | 1.38 (1.21, 1.58) | 1181 | 1.41 |
|  | MEP | PAEs | <0.01 | 1.17 (1.07, 1.27) | 1181 | 1.02 |
|  | MHBP | PAEs | 0.57 | 1.04 (0.90, 1.20) | 1180 | 1.55 |
|  | MHiBP | PAEs | 0.08 | 1.12 (0.99, 1.28) | 1180 | 1.37 |
|  | MHINCH | PAEs | 0.89 | 1.01 (0.87, 1.17) | 1181 | 0.64 |
|  | MiBP | PAEs | <0.01 | 1.23 (1.08, 1.40) | 1181 | 1.19 |
|  | MiNP | PAEs | 0.14 | 1.10 (0.97, 1.26) | 1181 | 0.77 |
|  | BP | Parabens | 0.17 | 0.89 (0.75, 1.05) | 1181 | 0.75 |
|  | EP | Parabens | 0.97 | <1.00 (0.91, 1.10) | 1181 | 0.41 |
|  | MP | Parabens | 0.58 | 1.02 (0.94, 1.11) | 1181 | 0.74 |
|  | PP | Parabens | 0.43 | 1.03 (0.96, 1.09) | 1181 | 0.44 |
|  | MPAH | PFASs | 0.68 | 1.04 (0.87, 1.24) | 1182 | 0.11 |
|  | n-PFOS | PFASs | 0.03 | 0.84 (0.71, 0.98) | 1181 | 0.69 |
|  | PFBA | PFASs | 0.59 | 0.88 (0.55, 1.40) | 602 | 0.21 |
|  | PFDA | PFASs | 0.63 | 0.96 (0.81, 1.13) | 1182 | 0.38 |
|  | PFHpA | PFASs | 0.72 | 1.14 (0.55, 2.40) | 605 | 0.20 |
|  | PFHxA | PFASs | 0.42 | 0.89 (0.67, 1.18) | 602 | 0.31 |
|  | PFHxS | PFASs | 0.16 | 0.89 (0.75, 1.05) | 1182 | 0.42 |
|  | PFNA | PFASs | 0.75 | 0.97 (0.79, 1.18) | 1182 | 0.30 |
|  | PFOA | PFASs | 0.50 | 0.93 (0.77, 1.14) | 1181 | 0.19 |
|  | PFUA | PFASs | 0.33 | 0.91 (0.76, 1.10) | 1182 | 0.33 |
|  | Sm-PFOS | PFASs | 0.21 | 0.90 (0.76, 1.06) | 1181 | 0.36 |
|  | BP-3 | Phenols | 0.39 | 1.03 (0.96, 1.11) | 1181 | 0.46 |
|  | BPA | Phenols | 0.03 | 1.15 (1.01, 1.30) | 1181 | 1.00 |
|  | BPF | Phenols | 0.63 | 1.02 (0.93, 1.12) | 1180 | 0.56 |
|  | BPS | Phenols | 0.04 | 1.12 (1.01, 1.24) | 1180 | 0.63 |
|  | TCC | Phenols | 0.22 | 0.95 (0.88, 1.03) | 1181 | 0.85 |
|  | TCS | Phenols | 0.30 | 1.04 (0.97, 1.11) | 1181 | 0.30 |

NA: Data for this categorization are not shown due to a large confidence interval or an inestimable result.

Table S10. Sensitivity analysis of linear regression analysis on the key chemicals and T, LDL-C among male adults

| **Endogenous chemicals** | **Exogenous chemicals** | ***P**** | ***β* (95%CI)** |
| --- | --- | --- | --- |
| LDL-C | MBP | <0.01 | -0.04 (-0.06, -0.02) |
|  | MCPP | <0.01 | -0.04 (-0.06, -0.02) |
|  | MEHP | 0.02 | -0.03 (-0.06, -0.006) |
|  | MiBP | 0.03 | -0.03 (-0.05, -0.003) |
| T | MBP | 0.02 | -0.03 (-0.06, -0.01) |
|  | MCPP | 0.01 | -0.03 (-0.05, -0.01) |
|  | MEHP | 0.02 | -0.03 (-0.06, -0.005) |
|  | MiBP | 0.02 | -0.03 (-0.06, -0.01) |

*: adjusted for sample weights.

Table S11. Sensitivity analysis of logistic regression analysis on the key chemicals and T deficiency among male adults

| **Exogenous chemicals** | ***P**** | **OR (95%CI)** |
| --- | --- | --- |
| MCPP | 0.02 | 1.13 (1.02, 1.26) |
| MBP | 0.02 | 1.16 (1.02, 1.31) |
| MEHP | 0.02 | 1.19 (1.03, 1.38) |
| MiBP | <0.01 | 1.21 (1.06, 1.38) |

*: adjusted for sample weights.

Table S12. Information of potential MIEs in males

| **Genes** | **Position** | **Median TPM** | **Rank^a^** |
| --- | --- | --- | --- |
| CYP11A1 | chr15:74337759-74367740 | 47.47 | 3 |
| CYP17A1 | chr10:102830531-102837533 | 14.57 | 2 |
| CYP19A1 | chr15:51208057-51338610 | 2.67 | 3 |
| CYP3A4 | chr7:99756960-99784265 | 1.033 | 6 |
| FGF8 | chr10:101770130-101780369 | 1.204 | 1 |
| FSHB | chr11:30231016-30235261 | 0.04357 | 4 |
| FSHR | chr2:48962157-49154537 | 3.08 | 1 |
| GH1 | chr17:63917200-63918838 | 2.843 | 5 |
| INS | chr11:2159779-2161341 | 0.2665 | 8 |
| LHCGR | chr2:48686775-48755730 | 0.8713 | 5 |
| NR0B1 | chrX:30304206-30309598 | 39.07 | 2 |
| NR5A1 | chr9:124481236-124507430 | 31.18 | 5 |
| SHBG | chr17:7613946-7633383 | 12.3 | 1 |
| StAR | chr8:38143649-38151265 | 73.51 | 3 |

a: the expression of genes in testis tissue compared with other tissues. MIEs, molecular initiating events; TPM, transcripts per kilobase million.

Table S13. Classification of phenotypes

| **Classification** | **Phenotypes/Ancestor terms** | **Phenotypes** | **Related genes** |
| --- | --- | --- | --- |
| KE1 | lipid metabolic process | cholesterol metabolic process | StAR/CYP3A4/CYP11A1 |
|  | lipid metabolic process | lipid catabolic process | CYP3A4/CYP19A1/INS |
|  | lipid metabolic process | negative regulation of lipid metabolic process | NR0B1/INS |
|  | lipid metabolic process | positive regulation of lipid biosynthetic process | StAR/FSHB/INS |
|  | lipid metabolic process | positive regulation of lipid metabolic process | StAR/FSHB/INS/INS |
|  | lipid metabolic process | regulation of lipid biosynthetic process | StAR/NR0B1/FSHB/LHCGR/NR5A1/INS |
|  | lipid metabolic process | regulation of lipid metabolic process | StAR/NR0B1/FSHB/LHCGR/NR5A1/INS |
|  | lipid metabolic process | steroid metabolic process | StAR/CYP3A4/CYP11A1/CYP17A1/NR0B1/FSHB/LHCGR/NR5A1/CYP19A1 |
|  | regulation of signaling | positive regulation of ERK1 and ERK2 cascade | FSHR/FGF8 |
| KE2 | cell differentiation | Leydig cell differentiation | NR0B1/NR5A1 |
|  | macromolecule localization | lipid localization | StAR/LHCGR/CYP19A1 |
|  | macromolecule localization | regulation of lipid localization | CYP19A1 |
|  | molecular function | hormone binding | SHBG/INS |
|  | molecular function | oxidoreductase activity, acting on paired donors, with incorporation or reduction of molecular oxygen | CYP3A4/CYP11A1/CYP17A1/CYP19A1 |
|  | molecular function | oxidoreductase activity, acting on paired donors, with incorporation or reduction of molecular oxygen, reduced flavin or flavoprotein as one donor, and incorporation of one atom of oxygen | CYP19A1 |
|  | molecular function | oxygen binding | CYP17A1/CYP19A1 |
|  | molecular function | receptor ligand activity | FSHB/GH1/INS |
|  | molecular function | signaling receptor activator activity | FSHB/GH1/INS |
|  | molecular function | steroid binding | StAR/CYP3A4 |
|  | molecular function | steroid hydroxylase activity | CYP3A4/CYP11A1/CYP17A1/CYP19A1 |
|  | regulation of cellular process | ERK1 and ERK2 cascade | FSHR/FGF8 |
|  | regulation of cellular process | hormone-mediated signaling pathway | NR0B1 |
|  | regulation of cellular process | inositol lipid-mediated signaling | FSHR/GH1/INS |
|  | regulation of cellular process | phosphatidylinositol 3-kinase signaling | FSHR/GH1/INS |
|  | regulation of cellular process | phosphatidylinositol-mediated signaling | FSHR/GH1/INS |
|  | regulation of cellular process | positive regulation of nuclear division | FGF8/INS |
|  | regulation of cellular process | positive regulation of phosphatidylinositol 3-kinase signaling | FSHR/GH1/INS |
|  | regulation of cellular process | regulation of nuclear division | FGF8/INS |
|  | regulation of cellular process | regulation of phosphatidylinositol 3-kinase signaling | FSHR/GH1/INS |
|  | regulation of cellular process | second-messenger-mediated signaling | LHCGR/INS |
|  | regulation of steroid metabolic process | positive regulation of steroid biosynthetic process | StAR/FSHB |
|  | regulation of steroid metabolic process | positive regulation of steroid metabolic process | StAR/FSHB |
|  | regulation of steroid metabolic process | regulation of steroid biosynthetic process | StAR/NR0B1/FSHB/LHCGR/NR5A1 |
|  | response to steroid hormone | cellular response to steroid hormone stimulus | NR0B1 |
|  | response to steroid hormone | cellular response to steroid hormone stimulus | NR0B1 |
|  | steroid metabolic process | androgen metabolic process | CYP3A4/CYP17A1/CYP19A1 |
|  | steroid metabolic process | regulation of steroid metabolic process | StAR/NR0B1/FSHB/LHCGR/NR5A1 |
|  | steroid metabolic process | steroid biosynthetic process | StAR/CYP3A4/CYP11A1/CYP17A1/NR0B1/FSHB/LHCGR/NR5A1/CYP19A1 |
|  | steroid metabolic process | steroid hormone biosynthetic process | CYP11A1/CYP17A1/FSHB/CYP19A1 |
| KE3 | developmental process | endocrine system development | NR0B1/FGF8/NR5A1/INS |
|  | developmental process | gonad development | NR0B1/FSHB/LHCGR/FSHR/FGF8/NR5A1/CYP19A1/INS |
|  | developmental process | male sex differentiation | NR0B1/FSHB/LHCGR/FSHR/FGF8/NR5A1/CYP19A1/INS |
|  | developmental process | reproductive structure development | NR0B1/FSHB/LHCGR/FSHR/FGF8/NR5A1/CYP19A1/INS |
|  | developmental process | reproductive system development | NR0B1/FSHB/LHCGR/FSHR/FGF8/NR5A1/CYP19A1/INS |
|  | regulation of hormone levels | hormone biosynthetic process | CYP11A1/CYP17A1/FSHB/LHCGR/CYP19A1 |
|  | regulation of hormone levels | hormone metabolic process | CYP3A1/CYP11A1/CYP17A1/FSHB/LHCGR/NR5A1/CYP19A1 |
|  | regulation of reproductive process | positive regulation of reproductive process | NR5A1/INS |
|  | reproductive process | development of primary male sexual characteristics | NR0B1/FSHB/LHCGR/FSHR/NR5A1/CYP19A1/INS |
|  | reproductive process | development of primary sexual characteristics | NR0B1/FSHB/LHCGR/FSHR/FGF8/NR5A1/CYP19A1/INS |
|  | reproductive process | male gonad development | NR0B1/FSHB/LHCGR/FSHR/NR5A1/CYP19A1/INS |
|  | reproductive process | regulation of reproductive process | NR5A1/INS |
|  | reproductive process | sex differentiation | CYP17A1/NR0B1/NR0B1/FSHB/LHCGR/FSHR/FGF8/NR5A1/CYP19A1/INS |
| KEGG pathways | cAMP signaling pathway | cAMP signaling pathway | FSHB/FSHR |
|  | steroid hormone biosynthesis | steroid hormone biosynthesis | CYP11A1/CYP17A1/CYP19A1/CYP3A4 |

Table S14. Energy analysis using the MM/PBSA method

| Frame | Binding (with DH) | MM (with DH) | PB | SA | COU (with DH) | VDW |
| --- | --- | --- | --- | --- | --- | --- |
| 90ns | -101.639(-96.764) | -186.670(-181.795) | 101.922 | -16.891 | -40.352(-35.476) | -146.318 |
| 91ns | -101.296(-94.634) | -185.313(-178.651) | 101.542 | -17.525 | -43.409(-36.747) | -141.903 |
| 92ns | -87.224(-82.653) | -175.702(-171.132) | 105.326 | -16.848 | -47.009(-42.439) | -128.693 |
| 90ns | -101.639(-96.764) | -186.670(-181.795) | 101.922 | -16.891 | -40.352(-35.476) | -146.318 |
| 91ns | -101.296(-94.634) | -185.313(-178.651) | 101.542 | -17.525 | -43.409(-36.747) | -141.903 |
| 92ns | -87.224(-82.653) | -175.702(-171.132) | 105.326 | -16.848 | -47.009(-42.439) | -128.693 |
| 93ns | -92.560(-88.208) | -170.396(-166.045) | 94.692 | -16.855 | -42.671(-38.319) | -127.725 |
| 94ns | -87.872(-84.972) | -167.721(-164.822) | 96.572 | -16.723 | -31.501(-28.601) | -136.221 |
| 95ns | -100.790(-96.109) | -188.092(-183.410) | 104.335 | -17.034 | -47.833(-43.152) | -140.258 |
| 96ns | -100.577(-95.438) | -177.274(-172.134) | 93.675 | -16.979 | -43.391(-38.251) | -133.883 |
| 97ns | -95.643(-90.372) | -183.592(-178.321) | 104.91 | -16.961 | -50.528(-45.257) | -133.064 |
| 98ns | -113.378(-109.066) | -194.538(-190.226) | 98.29 | -17.129 | -42.691(-38.379) | -151.846 |
| 99ns | -105.830(-103.448) | -186.878(-184.495) | 97.922 | -16.874 | -35.784(-33.401) | -151.094 |
| 100ns | -88.170(-84.812) | -177.443(-174.086) | 105.956 | -16.682 | -36.489(-33.132) | -140.953 |
| ΔH | -97.725(-93.316) | -181.238(-176.829) | 100.467 | -16.955 | -41.969(-37.560) | -139.269 |
| −TΔS | 8.450(7.486) | | | | | |
| ΔG | -89.276(-85.830) kJ/mol | | | | | |
| Ki | 2.289×10^-7^(9.187×10^-7^) nM | | | | | |

The unit of data in the table is kJ/mol.

ΔG = -RT ln(KA) = RT ln(Kd) ≈ RT ln(Ki).

Ki is not directly equivalent to IC50 or EC50 and should not be interpreted as an absolute pharmacological parameter.

Table S15. Assessment of the essentiality of KEs

| **Events** | **Evidence** | **PMID** | **WoE** |
| --- | --- | --- | --- |
| MIE | | | |
| Decrease, CYP11A1, CYP17A1, NR0B1, NR5A1, StAR | Direct evidence: Decreased CYP11A1, CYP17A1, NR0B1, NR5A1, StAR transcription leads to reduced T levels in mice and human Leydig cells, nuclear receptor transcription factors (NR0B1, NR5A1), converting cholesterol to T (CYP11A1, CYP17A1, StAR) regulate steroidogenic gene expression in Leydig cells, at the transcriptional level, steroidogenic enzymes are regulated by binding of NR5A1 to gene promoters. Knockdown of NR5A1 induces significant accumulation of neutral lipids and cholesterol while reducing androgen levels in the mouse Leydig cell lines. | 35177090[6], 22138857[7], 23480967[8], 34808194[9],  27455990[10] | High |
| KE1 | | | |
| Suppress, cholesterol metabolic process | Direct evidence: Cholesterol is the raw material for the synthesis of T by Leydig cells. | 34808194[9] | High |
| KE2 | | | |
| Suppress, steroid metabolic process | Direct evidence: T is synthesized by the steroid synthesis route. | 35595638[11], 34453734[12] | High |
| KE3 | | | |
| Reproductive dysfunction | Direct evidence: Testicular dysplasia leading to decreased T levels | 30821590[13] | High |

KE, key event; MIE, molecular initiating event; WoE, weight of evidenc

Table S16. Assessment of the evidence supporting KERs

| **KERs** | **Evidence** | **PMID** | **WoE** |
| --- | --- | --- | --- |
| MIE to KE1 | | | |
| Decreased CYP11A1, CYP17A1, NR0B1, NR5A1 and StAR, led to the suppression of cholesterol metabolic process | Biological plausibility: NR0B1, NR5A1 and CYP11A1, CYP17A1, StAR control steroid synthesis at transcriptional and synthetic levels, respectively. | 34808194[9] | High |
| KE1 to KE2 | | | |
| Cholesterol metabolism suppression led to steroid metabolism suppression | Biological plausibility: Cholesterol metabolism is upstream of steroid metabolism. | 27181934[14] | High |
| KE2 to KE3 | | | |
| Steroid metabolism suppression decreased hormone levels | Biological plausibility: Steroid synthesis pathway to synthesize T. | 27181934[14] | High |
| Steroid metabolism suppression delayed developmental and reproductive process | Biological plausibility: Blockage of steroid synthesis leads to disorders of sex development. | 20541150[15] | High |
| KE3 to AO | | | |
| Delayed developmental and reproductive process led to T deficiency | Biological plausibility: The first step in T production is the differentiation of Leydig cells from mesenchymal cells | 20541150[15] | High |
| Disturbed regulation of hormone levels led to T deficiency | Biological plausibility: Disturbances in the synthetic pathways of gonadal hormones lead to decreased T synthesis | 30205828[16] | High |

KER, key events relationship; KE, key event; MIE, molecular initiating event; AO, adverse outcome; WoE, weight of evidence

Table S17. Analysis of the association between molecular descriptors and disruptive effects on T biosynthesis

| **Descriptors^a^** | ***β* (95%CI)** | ***P*** |
| --- | --- | --- |
| BalabanJ | 0.02 (-0.03, 0.07) | 0.43 |
| BertzCT | 0.0001 (-0.00005, 0.0003) | 0.16 |
| EState_VSA1 | -0.001 (-0.004, 0.001) | 0.27 |
| EState_VSA10 | 0.002 (-0.002, 0.01) | 0.35 |
| EState_VSA2 | 0.002 (0.0005, 0.003) | 0.01 |
| EState_VSA3 | 0.0004 (-0.002, 0.002) | 0.65 |
| EState_VSA4 | -0.0003 (-0.001, 0.001) | 0.65 |
| EState_VSA5 | -0.0005 (-0.002, 0.001) | 0.55 |
| EState_VSA6 | 0.001 (-0.001, 0.003) | 0.33 |
| EState_VSA7 | 0.0002 (-0.001, 0.002) | 0.81 |
| EState_VSA9 | 0.002 (-0.003, 0.01) | 0.49 |
| ExactMolWt | 0.0001 (-0.0002, 0.0004) | 0.42 |
| HeavyAtomCount | 0.002 (-0.002, 0.01) | 0.39 |
| MolLogP | 0.003 (-0.01, 0.02) | 0.68 |
| MolMR | 0.0004 (-0.001, 0.001) | 0.43 |
| MolWt | 0.0001 (-0.0002, 0.0004) | 0.42 |
| NumHeteroatoms | 0.01 (-0.01, 0.02) | 0.41 |
| NumRotatableBonds | 0.003 (-0.002, 0.01) | 0.30 |
| NumValenceElectrons | 0.0002 (-0.0005, 0.001) | 0.49 |

a: BalabanJ: Balaban's J index; BertzCT:A topological index meant to quantify "complexity" of molecules; EState_VSA: MOE-type descriptors using EState indices and surface area contributions; MolLogP: Wildman-Crippen LogP value; MolMR: Wildman-Crippen MR value; MolWt: The average molecular weight of the molecule; NumHeteroatoms: Number of Heteroatoms; HeavyAtomCount: Number of heavy atoms of the molecule; NumRotatableBonds: Number of Rotatable Bonds; NumValenceElectrons: The number of valence electrons of the molecule.

Table S18. Screening of CYP17A1 inhibitors

| **Inhibitors** | **Specificity** | ***E* (kcal/mol)** | **SAR** | **RMSD(**Å**)** |
| --- | --- | --- | --- | --- |
| CFG920 | Low | NA | NA | NA |
| Seviteronel | Low | NA | NA | NA |
| Galeterone | Low | NA | NA | NA |
| ODM-204 | Low | NA | NA | NA |
| Abiraterone | Low | NA | NA | NA |
| BMS-737 | High | –7.6 | Miss | 0.31 |
| Orteronel | High | –7.6 | Match | 0.28 |
| SU-10603 | High | –7.3 | Miss | 0.36 |

*E*: Energy of binding with CYP17A1; SAR: structure–activity relationship with MCPP; RMSD: The average distance between atomic coordinates after alignment with MCPP.Table S19. PAEs, PFASs, Parabens, and Phenols included in the analysis

| **Classification** | **Name** | **Abbreviations** | **Formula** | **Structural formula** |
| --- | --- | --- | --- | --- |
| PFASs | Perfluorodecanoic acid | PFDA | C10HF19O2 | 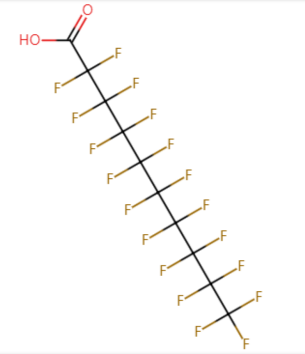 |
| PFASs | Perfluorohexane sulfonic acid | PFHxS | C6F13SO3H | 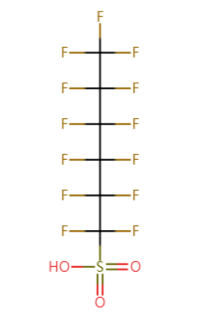 |
| PFASs | 2-(N-Methyl-perfluorooctane sulfonamido) acetic acid | MPAH | C11H6F17NO4S | 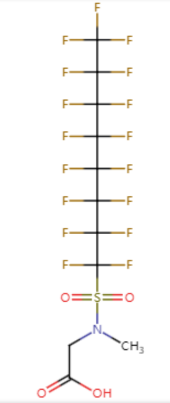 |
| PFASs | Perfluoroheptanoic acid | PFHpA | C7HF13O2 | 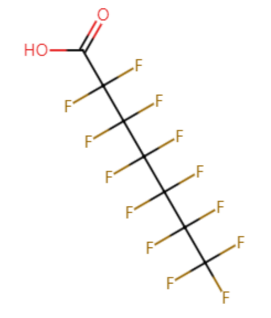 |
| PFASs | Perfluorononanoic acid | PFNA | C8F17COOH | 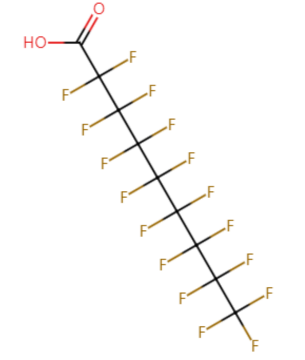 |
| PFASs | Perfluoroundecanoic acid | PFUA | C10F21COOH | 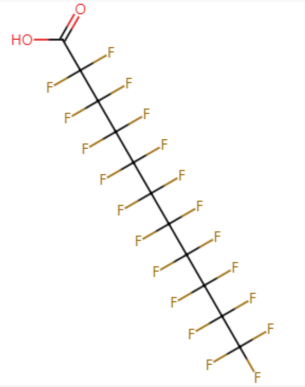 |
| PFASs | Perfluorobutanoic acid | PFBA | C4HF7O2 | 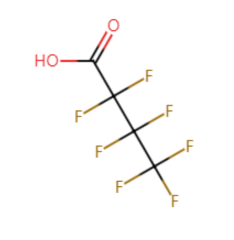 |
| PFASs | Perfluorohexanoic acid | PFHxA | C6HF11O2 | 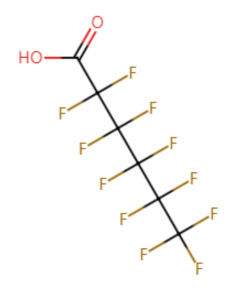 |
| PFASs | n-perfluorooctanoic acid | PFOA | C8HF15O2 | 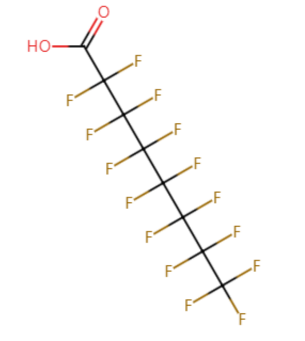 |
| PFASs | n-perfluorooctane sulfonic acid | n-PFOS | C8F17SO3H | 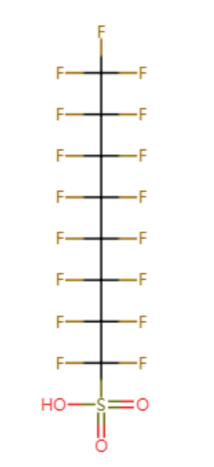 |
| PFASs | Perfluoromethylheptane sulfonic acid isomers | Sm-PFOS | - | - |
| Phenols | Benzophenone-3 | BP-3 | C14H12O3 | 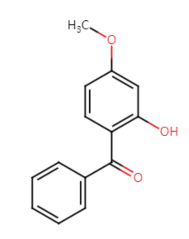 |
| Phenols | Bisphenol A | BPA | C15H16O2 | 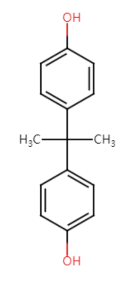 |
| Phenols | Bisphenol F | BPF | C13H12O2 | 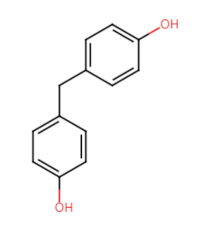 |
| Phenols | Bisphenol S | BPS | C12H10O4S | 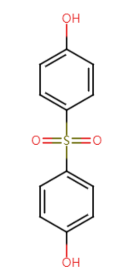 |
| Phenols | Triclocarban | TCC | C13H9Cl3N2O | 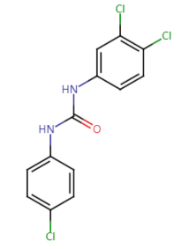 |
| Phenols | Triclosan | TCS | C12H7Cl3O2 | 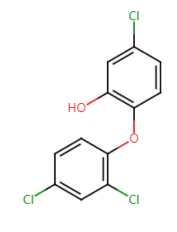 |
| Parabens | Butyl paraben | BP | C11H14O3 | 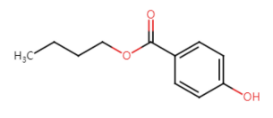 |
| Parabens | Ethyl paraben | EP | C9H10O3 | 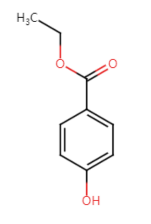 |
| Parabens | Methyl paraben | MP | C8H8O3 | 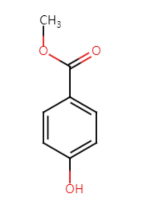 |
| Parabens | Propyl paraben | PP | C10H12O3 | 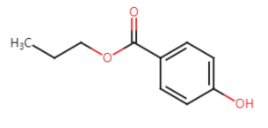 |
| PAEs | Mono(carboxynonyl) phthalate | MCNP | C18H24O6 | 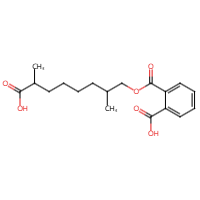 |
| PAEs | Mono(carboxyoctyl) phthalate | MCOP | C17H22O6 | 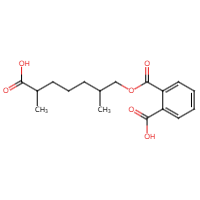 |
| PAEs | Mono-2-ethyl-5-carboxypentyl phthalate | MECPP | C16H20O6 | 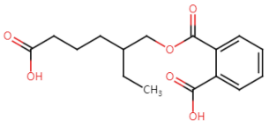 |
| PAEs | Mono-n-butyl phthalate | MBP | C12H14O4 | 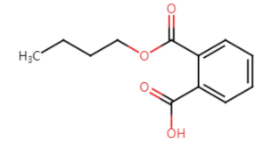 |
| PAEs | Mono-(3-carboxypropyl) phthalate | MCPP | C12H12O6 | 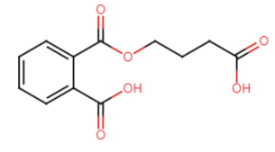 |
| PAEs | Mono-ethyl phthalate | MEP | C10H10O4 | 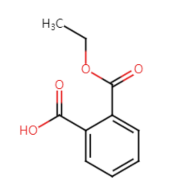 |
| PAEs | Mono-(2-ethyl-5-hydroxyhexyl) phthalate | MEHHP | C16H22O5 | 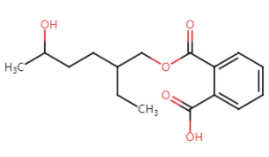 |
| PAEs | Cyclohexane-1,2-dicarboxylic acid monohydroxy isononyl ester | MHINCH | C17H30O5 | 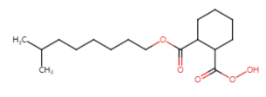 |
| PAEs | Mono-(2-ethyl)-hexyl phthalate | MEHP | C16H22O4 | 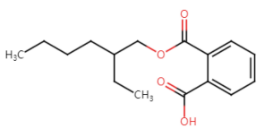 |
| PAEs | Mono-isobutyl phthalate | MiBP | C12H14O4 | 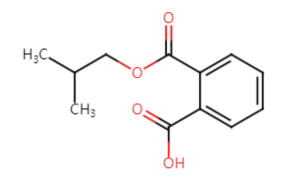 |
| PAEs | Mono-isononyl phthalate | MiNP | C17H24O4 | 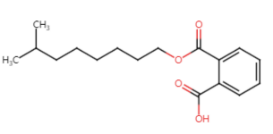 |
| PAEs | Mono-(2-ethyl-5-oxohexyl) phthalate | MEOHP | C16H20O5 | 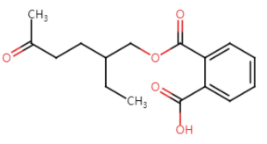 |
| PAEs | Mono-benzyl phthalate | MBzP | C15H12O4 | 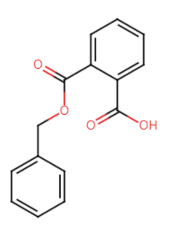 |
| PAEs | Mono-2-hydroxy-iso-butyl phthalate | MHiBP | C12H14O5 | 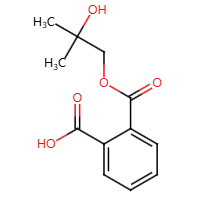 |
| PAEs | Mono-3-hydroxy-n-butyl phthalate | MHBP | C12H14O5 | 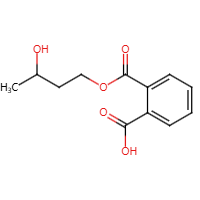 |
| PAEs | Cyclohexane-1,2-dicarboxylic acid-mono(carboxyoctyl) ester phthalate | MCOCH | C17H28O6 | 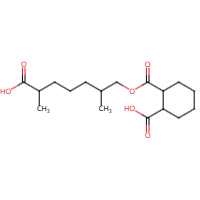 |

Table S20. Primer sequence

| **Gene** |  |  |
| --- | --- | --- |
| *Gapdh* | Forward | *5’-AGGTCGGTGTGAACGGATTTG-3’* |
|  | Reverse | *5’-TGTAGACCATGTAGTTGAGGTCA-3’* |
| *Cyp17a1* | Forward | *5’-CCAGAGAAGTGCTCGTGAAGAAGG-3’* |
|  | Reverse | *5’- CACAGTGAGTTGGCTTCCTGACAT -3’* |
| *Cyp11a1* | Forward | *5’- GCCAGCATCAAGGAGACACTGAG -3’* |
|  | Reverse | *5’- ACGAAGCACCAGGTCATTCACAG -3’* |
| *Star* | Forward | *5’- GGCATACTCAACAACCAGGAAGGC -3’* |
|  | Reverse | *5’-CTCCATGCGGTCCACAAGTTCTTC -3’* |

Movie S1 (separate file). The motion trajectory of MCPP and CYP17A1

**References**

[1] Li, H, M Yang, J Zhao, et al., Association of Per- and Polyfluoroalkyl Substance Exposure with Coronary Stenosis and Prognosis in Acute Coronary Syndrome. Environ Health (Wash), 2025. **3**(3): p. 291-307. <https://doi.org/10.1021/envhealth.4c00166>

[2] Kotsopoulos, J, AH Eliassen, SA Missmer, et al., Relationship between caffeine intake and plasma sex hormone concentrations in premenopausal and postmenopausal women. Cancer, 2009. **115**(12): p. 2765-74. <https://doi.org/10.1002/cncr.24328>

[3] Kuo, HK, SG Leveille, CJ Yen, et al., Exploring how peak leg power and usual gait speed are linked to late-life disability: data from the National Health and Nutrition Examination Survey (NHANES), 1999-2002. Am J Phys Med Rehabil, 2006. **85**(8): p. 650-8. <https://doi.org/10.1097/01.phm.0000228527.34158.ed>

[4] Wu, B, Y Jiang, X Jin, et al., Using three statistical methods to analyze the association between exposure to 9 compounds and obesity in children and adolescents: NHANES 2005-2010. Environ Health, 2020. **19**(1): p. 94. <https://doi.org/10.1186/s12940-020-00642-6>

[5] Machado, CM, AT Freitas, and FMJJobs Couto, Enrichment analysis applied to disease prognosis. 2013. **4**: p. 1-17.

[6] Jin, H, M Yan, C Pan, et al., Chronic exposure to polystyrene microplastics induced male reproductive toxicity and decreased testosterone levels via the LH-mediated LHR/cAMP/PKA/StAR pathway. Part Fibre Toxicol, 2022. **19**(1): p. 13. <https://doi.org/10.1186/s12989-022-00453-2>

[7] Ye, L, ZJ Su, and RS Ge, Inhibitors of testosterone biosynthetic and metabolic activation enzymes. Molecules, 2011. **16**(12): p. 9983-10001. <https://doi.org/10.3390/molecules16129983>

[8] Matzkin, ME, S Yamashita, and M Ascoli, The ERK1/2 pathway regulates testosterone synthesis by coordinately regulating the expression of steroidogenic genes in Leydig cells. Mol Cell Endocrinol, 2013. **370**(1-2): p. 130-7. <https://doi.org/10.1016/j.mce.2013.02.017>

[9] Choi, Y, EG Lee, G Lee, et al., Amodiaquine promotes testosterone production and de novo synthesis of cholesterol and triglycerides in Leydig cells. J Lipid Res, 2021. **62**: p. 100152. <https://doi.org/10.1016/j.jlr.2021.100152>

[10] Hatano, M, T Migita, T Ohishi, et al., SF-1 deficiency causes lipid accumulation in Leydig cells via suppression of STAR and CYP11A1. Endocrine, 2016. **54**(2): p. 484-496. <https://doi.org/10.1007/s12020-016-1043-1>

[11] Naamneh Elzenaty, R, T du Toit, and CE Flück, Basics of androgen synthesis and action. Best Pract Res Clin Endocrinol Metab, 2022. **36**(4): p. 101665. <https://doi.org/10.1016/j.beem.2022.101665>

[12] Ge, RS, X Li, and Y Wang, Leydig Cell and Spermatogenesis. Adv Exp Med Biol, 2021. **1288**: p. 111-129. <https://doi.org/10.1007/978-3-030-77779-1_6>

[13] Liu, M, Q Zhang, L Pei, et al., Corticosterone rather than ethanol epigenetic programmed testicular dysplasia caused by prenatal ethanol exposure in male offspring rats. Epigenetics, 2019. **14**(3): p. 245-259. <https://doi.org/10.1080/15592294.2019.1581595>

[14] Jones, S, A Boisvert, A Naghi, et al., Stimulatory effects of combined endocrine disruptors on MA-10 Leydig cell steroid production and lipid homeostasis. Toxicology, 2016. **355-356**: p. 21-30. <https://doi.org/10.1016/j.tox.2016.05.008>

[15] Mendonca, BB, EM Costa, A Belgorosky, et al., 46,XY DSD due to impaired androgen production. Best Pract Res Clin Endocrinol Metab, 2010. **24**(2): p. 243-62. <https://doi.org/10.1016/j.beem.2009.11.003>

[16] Darbandi, M, S Darbandi, A Agarwal, et al., Reactive oxygen species and male reproductive hormones. Reprod Biol Endocrinol, 2018. **16**(1): p. 87. <https://doi.org/10.1186/s12958-018-0406-2>
